# Disentangling the Effects of Craniotomy and Traumatic Brain Injury in Experimental Models: A Narrative Review

**DOI:** 10.3390/ijms27209186

**Published:** 2026-10-16

**Authors:** Viktoria Gribovskaya, Dianna Adzhemian, Pavel Rogozin, Veronika Nesterova, Artem Gureev, Denis Silachev

**Affiliations:** 1 Department of Genetics, Cytology, and Bioengineering, Voronezh State University, Voronezh 394018, Russia; vikagr04@gmail.com (V.G.); deanaajemyan@yandex.ru (D.A.); n3sterova.vero@yandex.ru (V.N.); 2 Brain Science Institute, Russian Center of Neurology and Neurosciences, Moscow 125367, Russia; parogozin@yandex.ru; 3 Kulakov National Medical Research Center of Obstetrics, Gynecology and Perinatology, 4 Akademika Oparina Str., Moscow 117997, Russia; 4 Department of Functional Biochemistry of Biopolymers, A.N. Belozersky Research Institute of Physico-Chemical Biology, Moscow State University, Moscow 119992, Russia

**Keywords:** traumatic brain injury, craniotomy, sham, naïve, cognitive impairment, biochemical markers, inflammation

## Abstract

Many widely used open-head models of traumatic brain injury (TBI) require craniotomy before injury, while sham-operated animals undergo surgery without impact and serve as controls. However, sham surgery itself may alter physiological, molecular, and behavioral parameters, complicating interpretation of TBI-specific effects. This narrative review, based on a structured PubMed search, synthesizes studies employing a three-group design to distinguish injury-related changes from those associated with craniotomy and other perioperative procedures. Evidence indicates that sham surgery produces outcome-, region-, and time-dependent effects that are generally milder, more localized, and more transient than TBI. These include neuroinflammatory responses, localized neuronal alterations, temporary sensorimotor and cognitive deficits, nociceptive changes, and shifts in gut microbiota. In contrast, TBI causes more severe abnormalities, including extensive neuronal damage, sustained astrogliosis, microglial activation, neutrophilic infiltration, mitochondrial dysfunction, and long-term dysbiosis. Most electrophysiological parameters are relatively unaffected by sham surgery, although selected changes have been reported. Importantly, differences between naïve and sham animals cannot be attributed exclusively to craniotomy, as anesthesia, analgesia, and surgical technique may contribute. Thus, sham surgery should not be considered a biologically neutral control. Inclusion of both naïve and sham groups is particularly important when distinguishing surgery-associated from injury-specific effects in preclinical studies.

## 1. Introduction

Currently, the treatment and diagnosis of traumatic brain injury (TBI) remain among the most significant challenges in modern medicine and neurobiology. TBI is a serious type of injury characterized by a high mortality rate and long-term post-traumatic complications [1]. Such injuries occur as a result of a blow to the head by an object or a collision of the head with a stationary obstacle, leading to the displacement of the brain inside the skull. These injuries often cause persistent cognitive and behavioral impairments, including memory, sleep, and attention disorders, slowed thinking, and chronic fatigue. TBI occurs across all age groups; however, the highest incidence is recorded in children aged 0–4 years, young adults aged 15–24 years, and the elderly over 65 years [2]. The average incidence rate of brain injury is approximately 939 per 100,000 people, with fatal outcomes occurring in 7–23% of cases [3]. The high mortality rate and the risk of developing neurodegenerative diseases highlight the need for an in-depth study of TBI pathogenesis to develop effective neurosurgical and pharmacological therapeutic strategies.

Following the primary mechanical injury that occurs at the moment of impact, a cascade of secondary processes develops, including inflammation, oxidative stress, mitochondrial dysfunction, and neuronal death. Despite a large number of studies, many mechanisms of secondary damage remain poorly understood. Therefore, modeling TBI in laboratory animals remains a crucial tool for reproducing clinically relevant patterns of injury and testing potential neuroprotective approaches.

**Figure 1:**
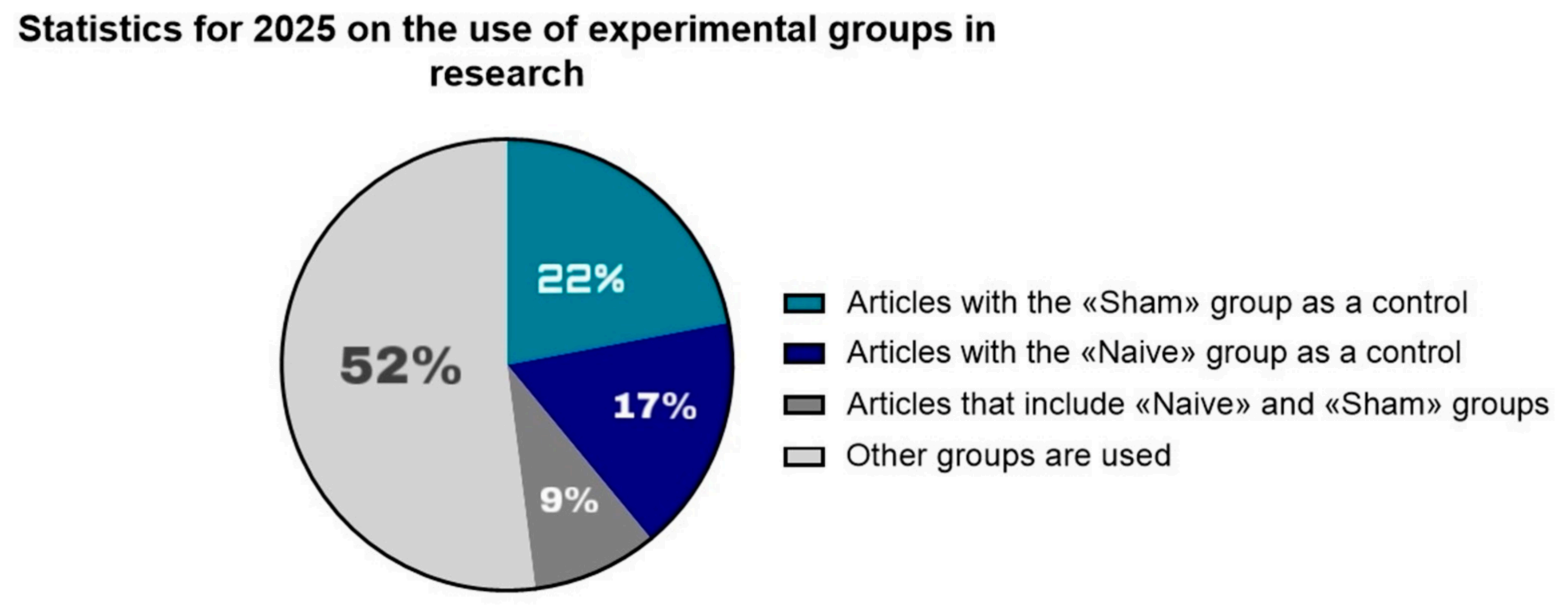
Statistics for 2025 on the use of experimental groups in research.

When creating experimental models of TBI, it is essential to consider a wide range of biological parameters, such as age, sex, body weight, and genetic characteristics of the animals, as well as the accuracy of localization and severity of the injury. When designing an experiment, it is also important to consider the choice of anesthetic agent and strict adherence to the protocol for its use to minimize possible effects that distort the results. Proper reproducibility of the model is a key factor in ensuring the reliability of the obtained data, as even minor deviations from the protocol can significantly distort the results [4]. A key component of experimental design is the proper selection of groups of laboratory animals. Currently, a tendency to select incomplete experimental groups is increasingly observed in some studies. In many studies devoted to the study of TBI, comparisons are made only between the injured (TBI) and sham-operated (sham) groups, which complicates the interpretation of the data (Figure 1).

With this approach, the effects caused by the trepanation procedure and anesthesia may be erroneously attributed to the consequences of traumatic exposure. One of the most pressing methodological issues in TBI research is the insufficient consideration of the impact of surgical intervention on physiological, metabolic, and other parameters. A significant number of studies clearly demonstrate the role of the skull opening procedure itself. It has been noted that changes in these parameters may be induced not by the impact itself, but by the surgical intervention itself [5,6,7]. This indicates the need for an additional control group. The inclusion of intact (naive) animals as an additional control group allows us to distinguish between the effects associated with the injury and those due to surgical intervention.

The aim of this narrative review is to critically examine studies that employed a three-group design (naïve/sham/TBI) to determine which behavioral, physiological, and molecular-biological changes result specifically from traumatic injury and which are due to surgical manipulations.

## 2. Cranial Trepanation Procedure and TBI Induction

### 2.1. Trepanation of the Skull: Craniotomy and Craniectomy

When simulating TBI in experimental animals, a preliminary craniotomy is necessary to access the site of the impact. Craniotomy is a surgical procedure that involves creating an opening in the skull to manipulate the brain. Trepanation is divided into two main types: craniotomy and craniectomy. Craniotomy involves removing a bone flap and then replacing it with a bone segment or artificial implant. When simulating TBI in animals, dental acrylic or bone wax is used as a replacement for the skull fragment [8]. Craniectomy, on the other hand, involves removing a bone flap from the skull to reduce intracranial pressure and improve cerebral circulation [9]. This type of trepanation has also yielded controversial data, which could be associated with cranial deformation. Mice that underwent craniectomy exhibited neurological impairment and pathophysiological dysfunctions [10].

**Figure 2:**
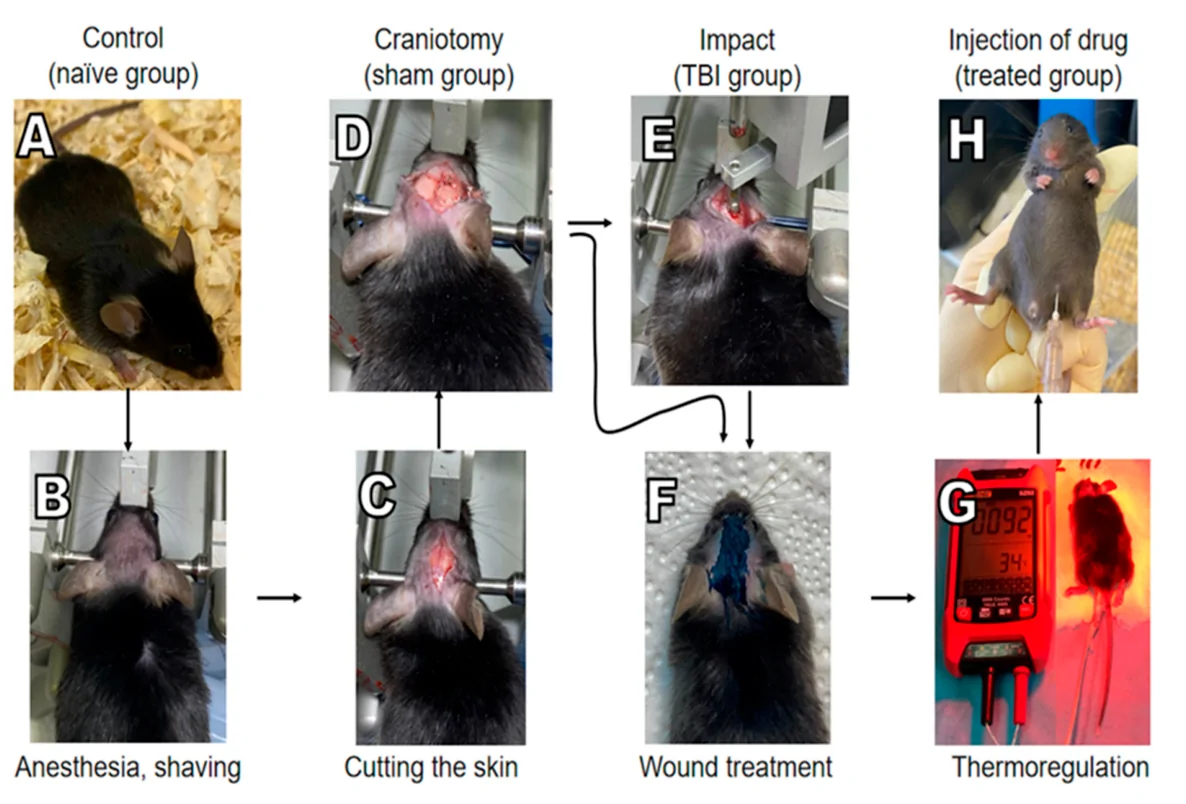
Stages of the craniotomy procedure in sham-operated and TBI groups. (**A**) The animal before the start of the procedure; (**B**) administration of an anesthetic, securing the animal in a stereotaxic apparatus, and removing fur from the head; (**C**) incision of soft tissues with a scalpel and retraction of the soft tissues away from the skull bones; (**D**) performance of a craniotomy; (**E**) induction of a traumatic brain injury (TBI); (**F**) closure of the wound; (**G**) monitoring of the animal’s body temperature; (**H**) “therapeutic window”: implementation of experimental procedures aimed at reducing the severity of primary and secondary injuries associated with the TBI.

Craniotomy allows researchers to target specific brain regions and precisely control impact parameters, such as location and force. Following numerous experiments, Swedish scientists in the 1980s described a protocol for a simple and safe method of performing craniotomy in white rabbits [11]. Since then, this procedure has been widely used in modeling TBI in various studies. The standard craniotomy procedure involved several stages. Initially, an anesthetic is administered to a fully intact animal (Figure 2A) to ensure analgesia and immobilization. Next, the animal is secured in a stereotaxic apparatus to ensure accurate injury induction. Subsequently, the fur on the animal’s head is removed (Figure 2B). A scalp incision is made along the midline using a scalpel to expose the skull. Soft tissues are carefully retracted from the bone (Figure 2C). Then, the craniotomy procedure itself is performed. A dental drill or a special trephine is used to create an opening of the required size and shape in the skull [12]. At this stage, it is crucial to control the drilling depth to avoid damaging the brain. The diameter of the opening depends on various factors, such as the impactor model used, the type of organism, etc. Typically, the opening diameter ranges from 2 mm to 8 mm [13,14] (Figure 2D). Subsequently, TBI is induced in the animal (Figure 2E), with various types discussed below. This stage is accordingly skipped for animals in the sham group. Next, wound closure is performed. After injury induction, the removed bone fragment is replaced, or a special material is used to cover the skull defect. Soft tissues are sutured, and the skin is closed with sutures or staples (Figure 2F) [12]. The main methods used for measuring body temperature in rodents include rectal thermometry, wireless temperature monitoring using implantable sensors, and infrared thermography [15]. Strict monitoring of the animal’s body temperature is necessary to prevent further brain damage and improve neurological outcomes [16]. Deviation from normothermic parameters can significantly affect the therapeutic outcome and distort the final results of the experiment; therefore, body temperature control is a mandatory element in experimental modeling of TBI (Figure 2G). Subsequently, during the therapeutic window, experimental procedures are usually carried out to reduce the severity of primary and secondary TBI-related injuries (Figure 2H). However, in this review, we focused specifically on the effects of TBI and trepanation on the characteristics of experimental animals and did not discuss the protective effects of therapeutic approaches described in the analyzed manuscripts.

### 2.2. Open Head Injury Models of TBI

Experimental models of TBI simulation can be divided into two types: open-head models and closed-head models. When reproducing open-head trauma in animals, researchers use craniotomy to gain access to the dura mater and deliver the desired impulse. This model allows for the study of focal impact, that is, the effect of physical force on a specific region of the brain. The main types of open-head models include controlled cortical impact (CCI) and fluid percussion injury (FPI) [17]. The advantages of open-head models include precise control of injury parameters such as force, velocity, depth, and pressure; high reproducibility of results; the ability to simulate varying degrees of injury severity; and the absence of a secondary impact that causes additional damage. However, this model of TBI simulation has several drawbacks. The need for surgical intervention in the form of craniotomy, the simulation of a minimal number of clinical cases of TBI, the inability to study behavior immediately after injury, and the lack of brainstem deformation are the main limitations of open-head models [18].

#### 2.2.1. The Controlled Cortical Impact Model

CCI model is a widely used model of TBI, developed almost 40 years ago to create a testing platform for determining various biomechanical properties of brain tissue subjected to direct mechanical deformation [19]. Initially used to model TBI caused by car accidents, CCI quickly evolved into a standardized method for studying TBI mechanisms and evaluating therapies. This model allows for precise control of injury parameters and effectively reproduces focal brain damage.

**Figure 3:**
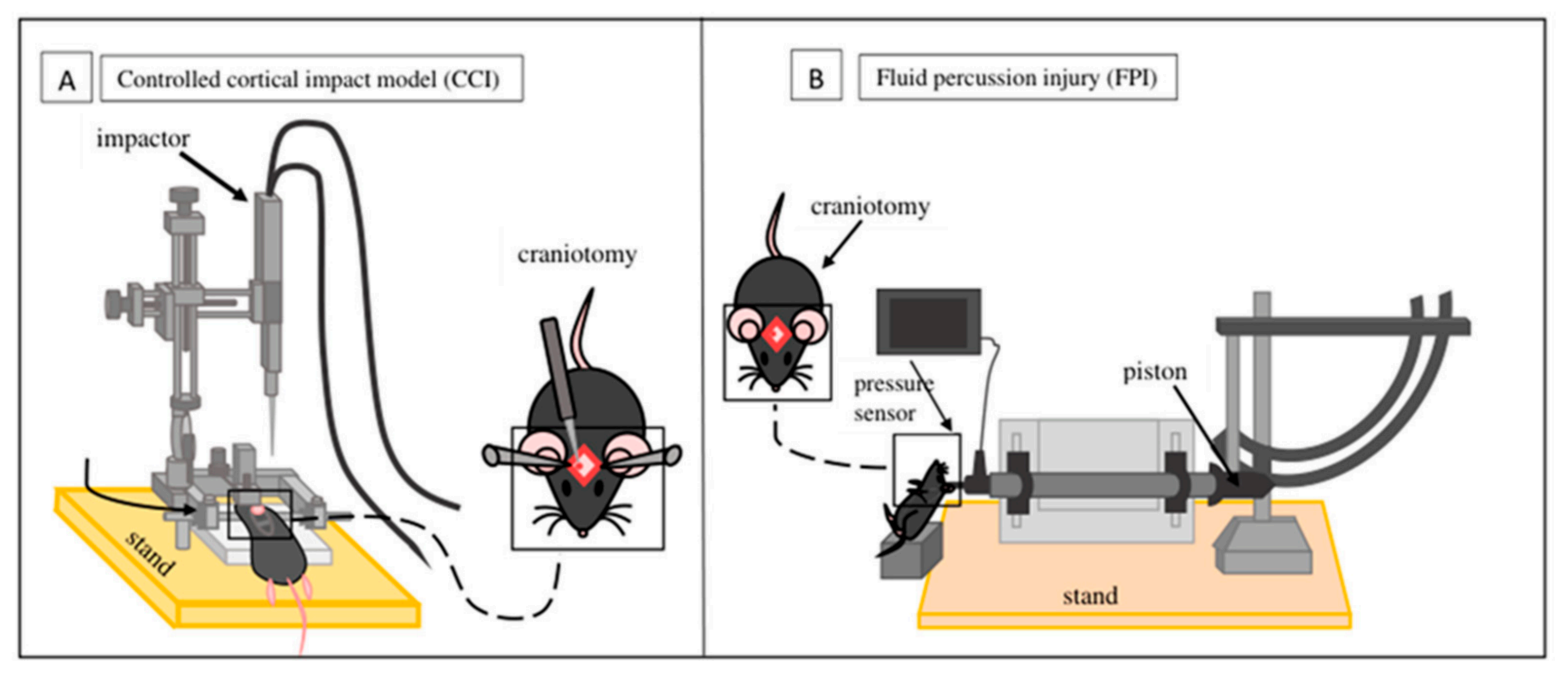
Types of open head injury models of TBI used. (**A**) The controlled cortical impact model (CCI); (**B**) fluid percussion injury (FPI).

A controlled-force and depth impact is applied to the exposed area of the skull using a specialized device—a stereotaxic impactor (Figure 3A). The stereotaxic impactor is a system designed to simulate TBI. It is a highly convenient tool for assessing the mechanisms of TBI in fundamental medical research. This system is a popular method for modeling TBI, controlled by pneumatic and electrical devices, which increases the accuracy and reproducibility of TBI modeling. A sensor is also used to automatically, accurately, and reliably determine the zero-interface reference point at the impact site before the start of the impact procedure. Parameters such as impact depth, velocity, and dwell time can be precisely controlled to ensure accuracy in the level of injury [20].

#### 2.2.2. Fluid Percussion Injury

FPI is a widely used model of brain injury. In this model, the injury is induced by the impact force of a fluid pressure pulse on the intact dura mater through a trepanation opening. The FPI procedure is performed by rapidly decompressing fluid, which strikes the brain through an opening in the parietal region of the skull (Figure 3B) [21]. FPI is typically applied either midline or parasagittally. Midline FPI induces diffuse TBI, whereas parasagittal FPI causes both focal cortical damage in the ipsilateral hemisphere and diffuse injury that also affects the contralateral hemisphere [22].

### 2.3. Closed Head Injury Models of TBI

The closed-head TBI model enables the simulation of impact without disrupting the tissue integrity of the animal’s head [8]. Traumatic impact on the intact skull may be induced either by direct impact (weight drop or piston impact), non-contact exposure (blast wave exposure), or inertial loading (head rotation in different planes) [18]. This model is more commonly used to reproduce clinical cases of TBI in humans. It induces diffuse injury similar to that occurring after naturally acquired TBI resulting from falls or road traffic accidents. Such brain injuries are manifested as diffuse vascular injury, diffuse axonal injury, hypoxic–ischemic encephalopathy, and generalized cerebral edema [23]. In addition, the closed-head TBI method most closely resembles the actual clinical presentation of human TBI, as it eliminates the stage of preliminary craniotomy. This model reproduces the mechanism of injury on the intact skull. This corresponds to the natural conditions under which TBI is sustained, where traumatic impact is not preceded by surgical preparation. However, closed-head TBI models do not provide the possibility of controlling injury parameters and are characterized by the development of diffuse axonal injury and a high risk of skull fracture [24]. The presence of a combination of various injuries and pathological alterations does not allow objective conclusions to be drawn.

**Figure 4:**
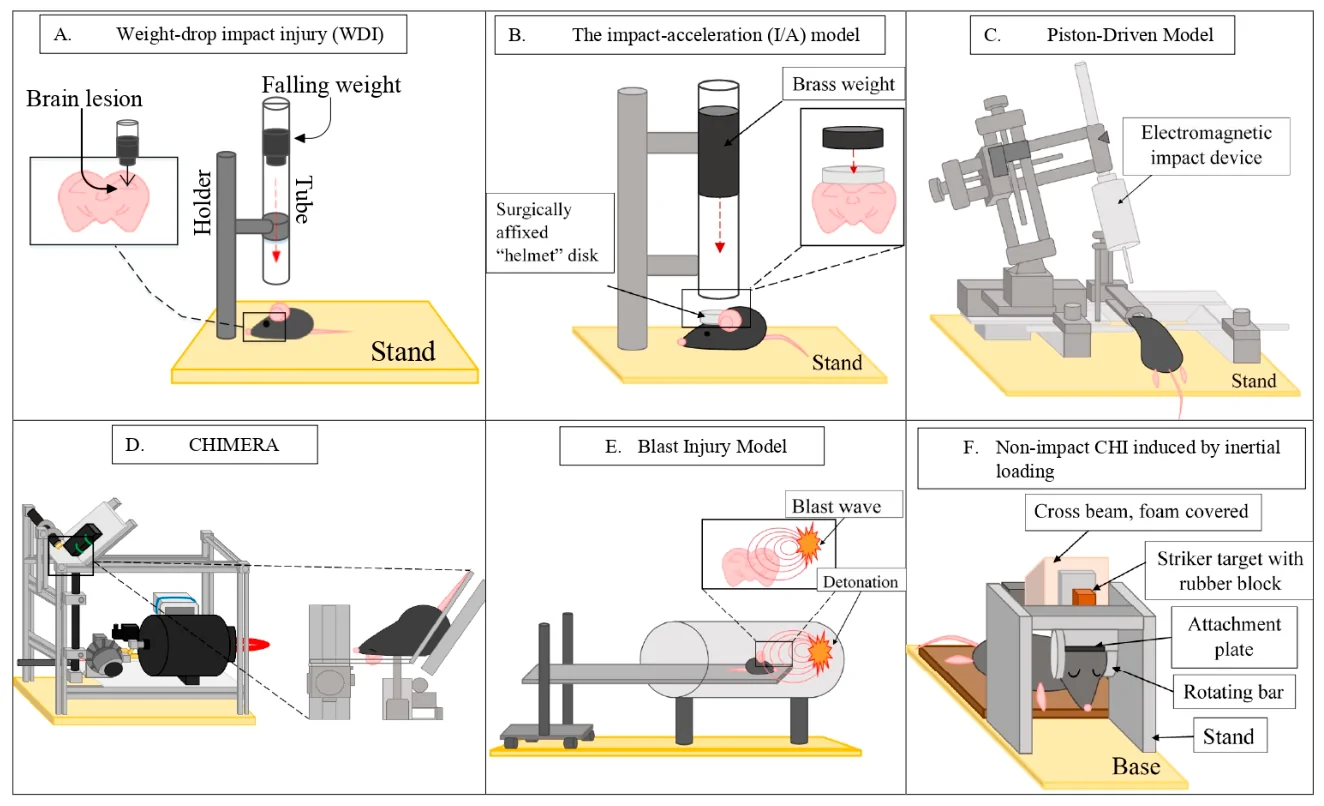
Types of closed head injury models of TBI used. (**A**) Weight drop-impact injury (WDI); (**B**) the impact-acceleration model (I/A); (**C**) piston impact model; (**D**) closed-head impact model of engineered rotational acceleration (CHIMERA); (**E**) the blast injury model; (**F**) non-impact TBI model.

To study closed-head TBI, models based on different methods of injury induction are used. The weight drop-impact acceleration injury (WDI) model is commonly used as a mechanical model of traumatic brain injury (Figure 4A). Injury is induced by allowing a weight of a specified mass to fall from a predetermined height onto the animal’s head [25]. The impact-acceleration (I/A) model is used to study diffuse axonal injury by inducing trauma with a weight freely falling from a predetermined height through a tube onto a metal disc fixed to the skull of a rodent (Figure 4B) [26,27]. Modeling injury by piston impact involves positioning the piston on the target area of the skull surface followed by injury induction (Figure 4C) [28]. To simulate clinical cases of mild TBI, the closed-head impact model of engineered rotational acceleration (CHIMERA) was developed (Figure 4D). This device simulates both impact and inertial loading of the head and thoracic region using a metal piston propelled by compressed air to deliver the impact [29]. The blast injury model is used to study injuries resulting from blast wave exposure. TBI is simulated using a device capable of generating a shock wave (Figure 4E) [30]. In the non-impact TBI model, diffuse brain injury is achieved by accelerating backward rotation of the head in the sagittal plane (Figure 4F). For this purpose, the rodent’s skull is fixed to a rotating bar that is deflected backward together with the head upon impact by a striker [31].

## 3. Comparison of the Effect of Trepanation and TBI on Physiological Parameters

### 3.1. Effect of Perioperative Factors on Body Weight of Experimental Animals in TBI Models

Longitudinal studies have revealed certain patterns concerning body weight changes in people who had suffered head injuries. Generally, phases of weight loss were observed, followed by stabilization and then an increase. A paradoxical observation was that patients with a low body mass index prior to injury tended to gain the most weight after trauma, whereas patients with a high body mass index before injury, on the contrary, lost weight following trauma [32]. In all the studies we analyzed, there was a decrease in both body weight and weight gain dynamics after the injury compared to the naïve mice group [14,33,34,35,36]. No reduction in weight gain was observed between the sham and TBI groups in the study by [36], and no difference was found 12 months after injury in the study by [34]. Interestingly, that study reported a decrease in body weight in the sham group compared to the naïve mice group [34]. Taken together, these data indicate that changes in body weight largely reflect the consequences of brain injury itself and, to a lesser extent, those of the surgical intervention (Table S1).

### 3.2. Effect of Perioperative Factors and TBI on Morphological Changes in the Brain

In most studies, TBI caused significant changes in the volume of the ipsilateral hemisphere [14] and in the volume of the dorsal cortex [37]. However, in the study by [38], no TBI-induced reduction in cortical tissue volume was found, although cortical layer thinning was observed compared to naïve mice. No structural changes in the cortex were observed in the sham group in the aforementioned studies. The absence of structural alterations in the sham group in these works confirms that, in some models, the contribution of the surgical intervention to large-scale cortical outcomes is minimal—at least within the observation periods and measurement methods used [38].

It is worth noting that cortical injuries can trigger a cascade of changes that also affect deeper brain regions. For example, in the TBI group compared to naïve mice, the volume of the corpus callosum above the lateral amygdala capsule decreased. At the same time, the volume of the amygdala was already reduced in sham mice compared to naïve mice [37]. The amygdala is of particular interest in the context of TBI, as there is evidence indicating that a reduction in amygdala volume is associated with greater impulsivity and other post-traumatic stress disorder (PTSD) symptoms in combat veterans who experienced mild TBI [39]. Meanwhile, no changes between the three groups were detected in hippocampal volume [37], as well as in ventricular volume [35]. However, in another study, the percentage of lateral ventricle volume relative to the total volume of the contralateral hemisphere was significantly increased in TBI mice compared to both sham and naïve mice. Moreover, the increase in ventricular volume correlated with the severity of brain injury. This may likely be due to both neuroinflammation and neurodegeneration in the ipsilateral hemisphere, leading to compensatory ventricular dilation [14]. In addition, TBI was accompanied by significant brain edema, reflected in an increase in brain water content; however, these differences were observed only between the TBI and naïve groups [35]. This pattern indicates that the surgical procedure itself does not reproduce a pronounced edematous response but rather reduces the contrast between TBI and sham. Therefore, interpreting edema-related metrics without including an intact control group may underestimate the true effect of injury.

Taken together, these data suggest that the sham group, in most cases, does not demonstrate large-scale morphological shifts relative to naïve animals. However, localized changes, such as those in the amygdala, do occur and may be clinically significant. In several metrics, differences between TBI and sham do not reach statistical significance, whereas the contrast between TBI and naïve reveals consistent effects (Table S1). This indicates that trepanation and associated factors may contribute moderately to structural and functional changes, which must be quantitatively controlled.

### 3.3. Neuronal Changes Induced by Perioperative Factors and TBI

Pathological neuronal damage is observed within minutes after TBI. Diffuse axonal injury (DAI), which occurs following TBI, leads to disruption of axonal integrity. Molecular processes of secondary injury, such as oxidative stress, excitotoxicity, inflammation, and edema, result in the persistence of axonal pathology over extended periods of time [40]. In this study, histological analysis of tissues was performed to assess the inflammatory response. Tissue disruption was observed only in TBI animals. However, microglial proliferation and immune cell infiltration near the lesion site were detected in both the TBI and sham groups. Moreover, in these groups, immunofluorescent microscopy revealed astrogliosis near the injury or trepanation site. Thus, TBI resulted in severe brain damage, while trepanation caused mild lesions that also led to the development of an inflammatory response similar to that seen with TBI [41].

FJC staining was performed to identify neuronal damage. In the naïve group, no neuronal staining was observed. In animals with TBI, intensely stained neurons were found in the cerebral cortex, dentate gyrus, and CA3 region of the hippocampus. Additionally, in this group, beaded deformation of neuronal dendritic processes was detected. Dendritic deformation may occur as a result of mechanical impact on tissue [42]. Also in TBI, signs of neuronal pathology were observed: compaction of neuronal cell bodies and accumulation of perivascular structures in the white matter. In the sham group, in the trepanation area, axonal terminals were detected in layers 1–2 and single axonal blebs in layers 3–4, as well as perivascular structures in the gray matter. Gallyas staining with neurosilver revealed axonal plaques and dendritic deformations. These changes indicate DAI in TBI, while similar but less pronounced morphological changes in sham suggest that trepanation itself may locally disrupt neuronal structure and affect the conductivity of axons and dendrites [43].

Using silver stain and MAP-2 immunoreactivity, neuronal damage was assessed. In the TBI group, the number of dead neurons in the ipsilateral cortex was significantly higher than in the other groups. In the sham group, an increase in dead neurons was also observed compared to the control. In the contralateral cortex, no significant differences were detected. Thus, TBI induces pronounced neuronal death in the injury area, while trepanation also affects their integrity, but to a lesser extent [6].

Microglia play an essential role as an immune component of the CNS. In turn, astrocytes participate in maintaining the brain’s ionic and water homeostasis, as well as in the functioning of the blood–brain barrier (BBB). However, following TBI, these structures undergo changes. Microglia begin to secrete proinflammatory and anti-inflammatory cytokines, while astrocytes alter the expression levels of specific proteins [44]. In the analysis of microglial cell and activated astrocyte fractions, this proportion was found to be increased in the TBI group compared to naïve and sham. Thus, the inflammatory process induced by TBI significantly affected the activation of astrocytes and microglia [45].

Thus, TBI induces pronounced neuronal degeneration with axonal–dendritic pathology and an active glial response. Trepanation without brain injury leads to local changes (astrogliosis, axonal swellings, perivascular structures) in the area of intervention, indicating the contribution of the surgical factor as a source of background damage. The three-group design (naïve, sham, TBI) is optimal for correct interpretation and should be used as a standard when analyzing neuronal outcomes (Table S1).

### 3.4. Effect of Perioperative Factors on Electrophysiological Parameters of Various Brain Regions in TBI Models

The study of neuronal activity using electrophysiological methods in vitro and in vivo complements morphological, behavioral, and biochemical approaches in the analysis of the consequences of TBI. The main methods include the recording of local field potentials (LFP), which allows assessment of the total extracellular activity of neurons and basic synaptic transmission, for example in the hippocampus, where plasticity (LTP, LTD) underlying memory is also studied. The second method is patch-clamp, which allows detailed investigation of the electrophysiological properties of an individual neuron and ionic currents across the membrane. For a review of these methods, see [46].

A considerable number of studies have been devoted to investigating the consequences of TBI using its various models and employing LFP or patch-clamp techniques. For a detailed review of electrophysiological parameter alterations after TBI modeling in different brain regions, see [47]. Most authors report a pronounced suppressive effect of TBI on synaptic plasticity in the CA1 and DG fields of the hippocampus [48,49,50,51]. Changes, often bidirectional, have been observed in baseline synaptic transmission in CA1 [49] and DG [49,51]. A shift in the excitability of CA1 pyramidal cells was detected [52], as well as impaired functioning of inhibitory interneurons in DG [49,53]. Electrophysiological recordings of neurons in other cortical areas also revealed post-TBI alterations: in the visual cortex in vivo [38], and in the sensorimotor and prefrontal cortices in vitro [54,55]. When evaluating various excitability parameters of layer 5 neurons in the somatosensory cortex of mice 1–2 days after injury, bidirectional changes were found between axotomized and non-axotomized neurons, suggesting a complex dynamic of post-injury alterations [56].

Against the background of intensive research on electrophysiological changes after TBI compared with sham animals, we found only six studies that included naïve controls. In the first in vitro study of TBI consequences, no differences in baseline synaptic transmission were observed between sham and naïve animals [48]; however, the same work showed a small (15 ± 4% sham, *n* = 8, vs. 30 ± 4%, *n* = 10 in control), but statistically significant difference in the magnitude of LTP in the CA1 region of the hippocampus between the naïve and sham groups. In the following study using the same FPI model in the same CA1 region in mice, no difference in LTP between naïve and sham groups was found [50]. In another study employing an FPI model in rats, no changes in LTP were detected in the DG of the hippocampus [57]. In a patch-clamp study of spontaneous inhibitory postsynaptic currents (sIPSCs) in granule and pyramidal cells in the DG and CA1 respectively, no differences were found between naïve and sham animals in various sIPSC parameters [49]. In the above-mentioned study [56], the authors reported no difference in 45 measured parameters of intrinsic excitability of layer 5 neurons in the somatosensory cortex between naïve and sham-operated animals on days 1 and 2 after FPI in ex vivo brain slices. In in vivo recordings of evoked synaptic responses and LTP in CA1 of rats, no differences between naïve (surgical control) and sham animals were observed [58]. Thus, it can be concluded that the influence of trepanation on electrophysiological parameters of hippocampal neurons is minimal or absent. Why does the sham–naïve LTP difference appear in one study but not in others? Several procedural variables plausibly account for this. First, the anesthetic and perioperative regimen: in [48], even animals receiving anesthesia and mechanical ventilation without craniotomy showed elevated hippocampal iNOS immunoreactivity, and the authors attributed the partial LTP reduction to pathological NO production secondary to the anesthetic/intubation procedure rather than to the craniotomy itself. Indeed, in this configuration (in vitro rat slices) halothane was used in only this one study. Second, the modest effect size (halving of LTP, still well within the inducible range) and small sample sizes (*n* = 3 vs. 5 rats, 15 ± 4% vs. 30 ± 4%, *p* = 0.035) place the comparison near the detection limit of in-vitro field-potential studies, so neither a positive nor a negative result should be treated as definitive.

Electrophysiological studies show that TBI induces pronounced disturbances in neuronal activity, including suppression of LTP, alterations in basal synaptic transmission, and neuronal excitability. The impact of perioperative factors as a separate factor is minimal and does not lead to significant changes in these parameters. Thus, the observed neurophysiological deviations are mainly attributable to traumatic injury rather than surgical intervention.

The assessment of motor evoked potentials (MEP) in the limb during transcranial electrical stimulation of the motor cortex can be interpreted as an indicator of the preservation of nerve tissue at the site of surgery. In the study by [59], MEPs were recorded from limb muscles before, during, and after simulated TBI using a proprietary method with an electronic cortical confusion impactor. No significant differences in MEP amplitude and latency were found between the sham and control groups. In addition, these parameters did not differ from the preoperative assessment. Thus, trepanation had no effect on the excitability of the motor cortex [59].

Post-traumatic epilepsy is a common consequence of TBI. In approximately half of patients with post-traumatic epilepsy, the first seizure occurs within the first year. And in 80% of patients with post-traumatic epilepsy, seizures begin within two years after the injury [60]. Electrocorticography (ECoG) or electroencephalography (EEG) studies are often used to search for biomarkers of this condition. In a study by [61], various ECoG parameters were evaluated in CD1 mice in a CCI model in the parieto-temporal cortex after injury in an attempt to find correlates of future epilepsy development. Mice from the control group had electrodes implanted but did not undergo craniotomy: 2 ipsilateral to the injury and 2 contralateral. Analysis of the nonlinear dynamics of the ECoG signal (Est-ED, Estimated Embedding Dimension) showed that craniotomy alone can cause a decrease in dimensionality.

Est-ED values in the sham group were consistently and significantly lower (~20%) than in the naïve group on all electrodes throughout the 96 h of observation after trauma. Est-ED is inversely proportional to the tendency of neurons to synchronize. Low dimensionality indicates a more synchronized, “predictable” state associated with epileptogenesis. However, the main predictor of developing epilepsy, the daily number of spikes/sharp waves, did not differ between the control and sham groups. Thus, trepanation is a significant intervention that does not affect long-term spike activity compared to naive control, but causes a persistent decrease in the dimensionality (Est-ED) of the cortical signal, indicating a change in the functional state of the cortex. This highlights the importance of using sham controls in such studies, as naïve mice do not fully reflect the electrophysiological consequences of the surgical procedure [61].

In summary, the electrophysiological parameters of brain cells in deep (hippocampus) and even superficial (various areas of the cortex) regions remained largely unchanged after trepanation. In the sham-control groups, animals with dura mater damage were excluded from the final sample, which means that inflammation and mechanical damage to brain tissue were minimal. This probably explains the absence of gross disturbances in cell function when studied in vitro. However, in the study by Di Sapia et al., 2023, a decrease in the dimensionality of the ECoG signal was found even in the craniotomy control [61]. The authors themselves do not devote a separate section to this issue in their discussion. However, they note that the biological causes affecting dimensionality are not yet known. The authors’ assumptions regarding the causes of dimensionality reduction include changes in connectivity or neuron death. Some of the studies we found do indeed indicate neuronal death and neuronal changes even after craniotomy (Table S2). Such subtle changes in the dynamics of the entire brain’s neural networks during craniotomy deserve attention in further research. The commonplace effects of cerebral edema, microdisturbances in the BBB, and inflammation associated with extensive perioperative factors cannot be ruled out either.

### 3.5. The Effect of Perioperative Factors and TBI on Peripheral Targets

A series of measurements of external urethral sphincter activity demonstrates differences between the groups already within the first day. In TBI, a pronounced decrease in amplitude of baseline, amplitude of bursting, firing rate of baseline, and firing rate of bursting is observed compared to naïve and sham, persisting for 1–2 weeks and corresponding to the acute and subacute phases of secondary injury. In sham, short-term fluctuations of the parameters are observed, reflecting a transient effect of surgical intervention and stress. Cystometry confirms these differences: within the first day in sham, non-voiding contractions increase, and their frequency and duration are elevated, whereas in TBI these parameters are lower relative to sham. Both conditions are accompanied by a reduction in peak voiding pressure compared to naïve, an increase in voiding duration in sham, and a shortening of the voiding interval in both groups. After one week, shortening of intervals persists in sham, and by the end of the month in TBI, voiding duration increases and bladder weight rises compared to naïve and sham, indicating persistent dysfunction in TBI and a transient nature of changes after trepanation [62].

These results may indicate that trepanation exerts a predominantly temporary influence on bladder function. FPI in rats can cause transient electroencephalographic suppression and loss of muscle tone, as well as inhibition of many reflexes and complex organized behaviors [63]. Micturition is associated with activation of the motor cortex, somatosensory cortex, cingulate gyrus, retrosplenial cortex, thalamus, putamen, insula, and septal nucleus [64,65,66]. After TBI in this study, these areas were likely affected by diffuse injury, contrecoup damage from the impact, and transmission of cerebrospinal fluid pressure into the ventricles, since the FPI site was located between the cerebral hemispheres and likely influenced spinal fluid pressure in the pons. However, an indirect effect of the injury on bladder function is also possible. For example, urinary incontinence is a common symptom in normal pressure hydrocephalus, which is believed to be caused by ventricular enlargement that distorts the fibers of the corona radiata [67]. These assumptions require further investigation to identify the underlying mechanisms of bladder dysfunction following TBI [62].

There are also data showing that TBI may cause noticeable damage to the ultrastructure of the tracheobronchial epithelium. Ultrastructural damage is obvious at 2 h, deteriorating with time. No statistically significant differences were observed between the control and sham groups [68]. The combination of urodynamic and respiratory parameters indicates different resistance of systems to surgical and traumatic impacts (Table S3). Trepanation induces short-term functional shifts in the micturition circuit but does not lead to persistent organ remodeling. In the airways, significant ultrastructural damage is observed after TBI and absent in sham animals, emphasizing the systemic nature of trauma consequences.

## 4. Comparison of the Effects of Craniotomy and TBI on Animal Behavioral Characteristics

Behavior after TBI cannot be described by a single deficit; its profile is largely determined by the topography and network architecture of the lesion. The hippocampus and adjacent medial temporal structures determine spatial learning and episodic memory [69]; the prefrontal–striatal circuits are responsible for rule flexibility, strategy, and impulsivity [70]; the amygdaloid complex—for fear and anxiety [71]; the sensorimotor cortex, cerebellum, and vestibular nuclei—for balance, gait, and precise motor control [72]; and the hypothalamo-thalamo-cortical loops—for sleep and circadian rhythms [73].

### 4.1. The Effect of Perioperative Factors on Cognitive Functions in TBI

TBI is characterized by the occurrence of cognitive impairments. The resulting damage may subsequently lead to altered mental states, memory dysfunction, and pathological processes disrupting normal nervous system function [74]. Memory is a cognitive function that reflects the ability to acquire, store, and retrieve information obtained through sensory experience. It consists of a set of interconnected processes, including encoding, storage, and retrieval [47]. The hippocampus plays a key role in memory formation. Together with adjacent structures such as the entorhinal cortex, perirhinal cortex, and parahippocampal cortex, it forms the hippocampal complex [75].

Many secondary effects of TBI cause both structural and functional damage to the hippocampus. This leads not only to long-term memory impairments but may also affect other types of memory. The CA1 and CA3 regions of the hippocampus are especially sensitive to the effects of physical force. These regions are involved in learning, memory formation, and recall [76]. The effects of TBI on learning and memory in rodents are commonly assessed using the Barnes maze (BM), the eight-arm radial maze or radial water maze (RWM), and the Morris water maze (MWM).

In the MWM, the main indicator for assessing spatial memory performance is the latency to find the platform after the training phase. Depending on the experimental protocol, researchers can assess spatial learning and memory, working memory, and episodic-like memory [77]. Studies of latency to find the platform have shown that working memory performance worsens in TBI animals compared to naïve and sham groups [34,78,79]. In other experiments, a decline in spatial learning and reference memory was observed only in the TBI group [35,80], though this effect appeared to be transient [35]. Similarly, an increase in PC scores, which reflect memory impairment, was found six months after injury [81]. The number of points indicator reflects the use of spatially dependent platform search strategies. A higher score corresponds to intact spatial memory. A decrease in this indicator in the TBI group relative to the control indicates a violation of spatial learning and memory in injured animals [78].

As additional parameters, researchers often examine time spent in the platform quadrant and number of passes through the platform area. The first parameter reflects the ability of animals to remember the platform location, while the second indicates their exploratory activity in the target zone. Analysis of these indicators revealed spatial learning and memory impairments in rodents from the sham group [35,80]. The obtained results of spatial reference memory (latency to find platform and time spent in the area around the new position of the platform) indicate that trepanation in sham induces moderate impairments in spatial orientation without a pronounced effect on memory. At the same time, TBI leads to more pronounced and persistent deficits affecting episodic-like, working, and spatial memory [80].

RWM assesses spatial learning and memory. Cognitive deficits (latency to find platform, number of errors, and errors to find the previous trial’s platform) increased only in TBI animals. In sham vs. naïve, an increase in number of errors to find cued or hidden platform and latency to the cued or hidden platform (averaged over 2 days of training) was observed. This indicates that trepanation induces temporary difficulties in task acquisition at the early stage, which are subsequently compensated. A possible effect of surgical intervention on motivation and the speed of sensory information processing is also reflected in changes in decision speed in sham [80].

BM test assesses spatial learning and memory based on rodents’ natural tendency to seek shelter and avoid open spaces. Its main advantages include lower stress levels compared to water mazes and greater suitability for studying terrestrial rodents due to its dry nature [82]. Using this test, it was shown that TBI mice, compared to naïve ones, made significantly more errors prior to target during both the learning and reversal learning phases. The sham group, in turn, occupied an intermediate position in terms of cognitive dysfunction severity between intact and TBI animals [37].

The novel object recognition test (NORT) is based on rodents’ instinctive preference for exploring novel objects. If the animal remembers a previously explored object, it will spend more time examining a new one [83]. When studying short-term recognition memory using the discrimination ratio and time spent exploring the novel object, no significant differences were observed among groups, suggesting that TBI and trepanation did not affect short-term recognition memory [6]. In another study, discrimination index was used to assess retention memory. Rabbits in the TBI group spent less time exploring the novel object, indicating retention memory impairment [14]. In another study, duration of objects’ investigation in Session 1 and Session 2 showed that TBI and sham did not demonstrate a preference between the novel and the familiar object, unlike naïve, indicating impairment of recognition memory. At the same time, comparison of the distance covered revealed increased locomotor activity in TBI and sham in Session 2. This indicates preservation of the response to novelty alongside a deficit in recognition memory [80]. Overall, hippocampal damage resulting from TBI leads to impairments in retention and recognition memory but does not affect short-term recognition memory. Damage to the dura mater during trepanation may also contribute to recognition memory disturbances [84]. The results of the NORT in another study revealed a decrease in the discriminatory ratio in the TBI group compared to the sham and naïve groups. This indicates an impairment of short-term memory resulting from damage to the hippocampal region caused by TBI [85].

The T-maze spontaneous alternation test assesses spatial working memory based on rodents’ natural preference for novelty. To explore a new arm of the maze, the rodent must remember which arm it has previously visited [86]. Analysis of correct alternation showed that TBI-related damage leads to cognitive impairments, particularly in spatial working memory [14].

The attentional set-shifting test (AST) is a behavioral assay used to measure cognitive flexibility. The obtained data indicate that the test may be insufficiently sensitive to detect cognitive deficits after TBI. In TBI compared to naïve, an increase in total response error rates in AST was observed, especially at the R1 stage, which indicates impairment of reversal learning. At the same time, the effect was observed only at an impact depth of 2.8 mm, but not at 2.6 mm. This indicates that neither trepanation nor less severe injury substantially affects adaptability, or that AST has limited sensitivity [87].

To study the learning process in animals, a test for identifying the conditioned passive avoidance reflex was used. When entering the dark compartment, the animal receives an electric shock, which induces an avoidance reflex. The assessment of the preservation of this reflex over time serves to evaluate learning ability and memory [88]. This test revealed an increase in the latent time of transition in the TBI group compared to sham and naïve, indicating motor impairments as a result of head injury. On the 30th day, a decrease in the latent time of transition was observed in the TBI group compared to the naïve group, which indicates long-term memory impairment caused by TBI [85] (Table S4).

### 4.2. The Role of Perioperative Factors in Stress-Related and Exploratory Behavior of Rodents in TBI Models

After TBI, the most frequently occurring anxiety disorders are generalized anxiety disorder (GAD) and PTSD [89]. In addition, individuals who have suffered brain injuries often exhibit impulsive and risk-taking behavior [90,91]. The open field (OF) test is often used to assess anxiety in rodents, based on the conflict between exploration of a novel environment and avoidance of open spaces. Low anxiety is manifested by frequent пребывание in the central zone, active rearing in the center, exploration of burrows, as well as a low frequency of defecation and grooming. High anxiety is characterized by opposite parameters [92].

During this test, naïve rodents exhibited natural anxiety, avoiding open spaces. Animals with TBI demonstrated more risk-taking behavior, whereas sham occupied an intermediate position. Trepanation exerts a lesser effect on behavior than TBI; therefore, sham parameters are closer to naïve than to TBI, with a possible moderate increase in risk-taking after surgery [37]. Assessment of locomotor activity in the OF test revealed no significant behavioral alterations resulting from either TBI or trepanation [14]. The OF test conducted in the following study revealed a decrease in locomotor activity speed in the TBI group compared to the sham and naïve groups. Thus, the obtained data indicate a disruption of exploratory behavior as a result of TBI [85].

When assessing distance traveled, it was established that TBI increases locomotor activity, likely due to damage to structures responsible for motor control. An increase in number of wall rearing in TBI reflects a propensity for risk-taking behavior. In the OF test, a decrease in time spent in center per entry was also shown in sham vs. naïve and TBI vs. naïve, indicating the formation of basal anxiety both in TBI and after trepanation [6].

Similar findings were obtained using the elevated plus maze (EPM) test, which evaluates avoidance behavior. Like the OF, it is based on an approach–avoidance conflict, where the animal must choose between avoiding open arms and exploring new areas [93]. The number of entries into open arms was higher in TBI animals compared with naïve ones, while mice with trepanation again demonstrated intermediate results. These consistent outcomes support the conclusions drawn from the OF test [37].

The multivariate concentric square field (MCSF) test is similar to other behavioral paradigms described above. Its high level of novelty stimulates exploratory behavior, and the variety of shelter and exploration zones provides a more comprehensive behavioral profile than traditional tests [94]. In the MCSF test, the naïve and sham groups were not combined due to higher exploratory activity in naïve. When comparing TBI vs. sham, TBI showed an increase in exploratory behavior (trial 2) and risk-taking (trials 1 and 2), as well as a decrease in shelter seeking (trial 2), indicating a propensity for risk-taking behavior after head injury. These results are consistent with the data from OF and EPM [95].

The light–dark box test is used to detect anxiety-like behavior in animals. This test is based on rodents’ aversion to brightly illuminated areas and their exploratory behavior [96]. During the study, it was noted that TBI animals spent less time exploring the light box compared to naïve ones. This indicates the formation of anxiety-like behavior in TBI. In turn, the sham group occupied an intermediate position, which suggests their tendency toward anxiety-like behavior [97]. In behavioral testing: light–dark box task, freezing during tone (%) and extinction trial number (within session) did not show notable differences between groups, suggesting that this aspect might not have been affected by TBI. Compared to Sham, TBI showed an increased number of extinction trials within a single session. Mice after trepanation demonstrated the same number of extinction trials as naïve mice, which may indicate that surgical intervention does not impair fear extinction [98].

The study of the behavioral indicator nest building is used to assess the condition of rodents. The quality and complexity of the nest serve as indicators of pain and stress experienced by the animals—the better the nest, the higher the score [99]. When analyzing the test score, it was concluded that TBI, as well as trepanation, resulted in difficulties in nest construction. This indicates the presence of stress and pain sensations in rodents. However, these effects were neutralized over time during the recovery period after injury [100].

Thus, the TBI group more often shows increased exploratory activity and a tendency toward risk-taking behavior. These results may be associated with increased catecholaminergic activity as a consequence of TBI. Brain injury can affect the regulatory functions of catecholamines, leading to behavioral changes and a propensity for risk-taking [101]. In turn, sham animals may show a slight increase in either risk-taking or anxiety-like behavior due to the surgical intervention that induces a stress response. As a result, the hypothalamic–pituitary–adrenal (HPA) axis is activated [102]. The hypothalamus produces corticotropin-releasing hormone (CRH), which stimulates the secretion of adrenocorticotropic hormone (ACTH). ACTH promotes the synthesis and release of cortisol. Prolonged cortisol release leads to disturbances in stable psychological states [103].

TBI in humans is often accompanied by the development of various forms of fear, including PTSD [34]. Regarding fear conditioning and fear extinction in TBI, a lower level of freezing is observed at the 7th CS, and after 6 weeks, an increase in freezing compared to sham and naïve. This may indicate impairments in the consolidation, storage, and retrieval of fear, likely associated with damage to the amygdala. No differences are observed between sham and naïve; however, after 6 weeks, a moderate decrease in freezing is noted in the sham group, which may indicate a weak delayed effect of perioperative factors. [37].

Post-traumatic headache acquired as a result of TBI is a secondary headache that develops within a week after injury [104]. As a result of TBI, periorbital allodynia may develop, associated with increased pain sensitivity. To assess it, the Periorbital von Frey sensory test was used: a head withdrawal was considered a response, and absence of reaction was considered absence of movement. A decrease in the allodynia threshold was observed in TBI for up to 3 months and in sham up to day 35 compared to naïve. This indicates that both head injury and trepanation reduce the pain threshold, while sham shows a more pronounced positive dynamics of recovery of sensitivity [105] (Table S5).

### 4.3. The Effect of Craniotomy and TBI on Social Behavior

TBI can lead to the development of behavioral and social impairments. Social dysfunction represents a decrease in the ability to socialize and the emergence of difficulties in communication and interaction with others. TBI sustained in childhood later leads to a decreased quality of life due to difficulties integrating into society [106]. The Three-chamber social motivation task is used to assess social memory [107]. The analysis of preferences in enclosure investigation did not reveal differences between the groups under consideration [80].

The social recognition task is a test aimed at studying social behavior and assessing social recognition memory. It is based on the preference of rodents to investigate a new conspecific rather than a familiar one [108]. When studying the latency to 1st social encounter, it was noted that rats after TBI spent significantly more time initiating the first social contact than naïve animals. The duration of social interactions was reduced in the TBI group compared to sham [80]. Impairment of social recognition in TBI is consistent with the memory deficits identified in other tests and is associated with damage to white matter in diffuse axonal injury, disrupting connections between regions processing social information. In sham, no signs of social dysfunction are observed, indicating that trepanation alone is insufficient to induce impairments in social interaction [109] (Table S6).

### 4.4. The Effect of Perioperative Factors and TBI on Activity Cycles

One of the most common consequences of TBI is sleep–wake disturbances, manifesting as insomnia and hypersomnia. Forty to eighty percent of people who have sustained head injuries report difficulties falling or staying asleep, lack of energy after sleep, fatigue, and sleepiness. These disturbances can lead to failures in cognitive function [110].

In another study, EEG analysis of sleep and wakefulness was conducted over 1 week and 1–2 months after TBI. During the first week, an increase in REM was observed in sham compared to TBI and naïve. After 1–2 months, wakefulness time decreased in sham, while after trepanation the duration of NREM increased. Analysis of circadian rhythms (6 intervals of 4 h) showed that during the first week, wake duration increased in naïve (18:30–22:30), NREM duration increased in TBI (18:30–22:30), and REM duration increased in sham almost throughout the entire day, except for 22:30–02:30. After 1–2 months, wake duration increased in TBI (18:30–22:30), in naïve (22:30–02:30), whereas in sham NREM duration increased (18:30–02:30), and in TBI REM duration decreased (18:30–22:30) [111]. Although no pronounced overall changes in the sleep–wake cycle were observed after TBI, time-resolved analysis revealed alterations in wakefulness, NREM, and REM sleep during specific circadian intervals. In turn, an increase in REM sleep phase during the first week in rodents after trepanation may indicate the presence of active neuroplasticity processes. This is necessary for the recovery of the brain area damaged by surgical intervention [112]. The increase in slow-wave sleep during 1–2 months after trepanation reflects long-term restorative processes in brain tissue [113]. During the analysis of sleep spindles, a decrease in indicators such as average spindle duration and NREM spindle power was found both during the first week and at 1–2 months in the TBI and sham groups compared to the naïve group. The reduction in spindle activity indicates structural brain impairments resulting from both TBI and sham conditions. However, the increase in spindle amplitude during the first week in the sham group [111] serves as an indicator of the recovery of cognitive functions impaired due to surgical intervention [114].

In another study, delta activity was examined throughout the day. It was shown that the mean values for 1 week and for 1 or 2 months were increased in the TBI and sham groups compared to the naïve group [115]. During the night, the delta range is characterized by a low amplitude level of 0.5–3 Hz. Therefore, elevated delta rhythm values serve as a sign of pathological development [116]. In the study under consideration, the results of average delta activity measurements indicated impairments resulting from both TBI and trepanation [115].

On one hand, excessive alcohol consumption is a risk factor for TBI due to decreased coordination and delayed reaction under intoxication. On the other hand, as a result of head injury, many negative factors arise: worsening psychological state, pain, and anxiety, and a person may increase alcohol consumption in an attempt to cope with these problems [117]. In turn, alcohol consumption after TBI slows recovery processes and aggravates pathological changes [118].

Post-TBI operant drinking test showed an increase in the sum change from baseline after TBI in the TBI vs. naïve group. Animals divided into Quartiles 1–4 based on baseline alcohol consumption demonstrated: in Q1—an increase in consumption after TBI and trepanation (sham vs. naïve and TBI vs. naïve), in Q2–Q3—differences only between TBI vs. naïve, and in Q4—no significant differences were detected. Correlation analysis showed that higher baseline alcohol consumption is associated with an increase after TBI and trepanation. Overall, TBI is associated with an increase in alcohol consumption after injury [97]. Based on these results, it can be concluded that neuroinflammation and neuronal degeneration at the site of injury lead to an increase in alcohol consumption. The rise in alcohol intake after TBI is associated with the brain’s attempt to compensate for neurochemical imbalance and to reduce negative emotional states [119] (Table S6).

### 4.5. The Effect of Perioperative Factors on Sensorimotor Parameters of Animals in a Model of TBI

Sensory cortical areas control movements through feedback. In TBI, their connections with motor areas are disrupted, leading to impaired gait, balance, and an increased risk of falls. Postural stability depends on the processing of visual, vestibular, and somatosensory information [120].

To assess the sensorimotor cortex and thalamus, neuroscore tests are used, including evaluation of reflexes (limb flexion, placing reactions, hindlimb grasp reflex). Normal is scored as 0 points, impairment as 1. In rodents in the sham group, a transient deficit is observed in the first days after surgery, whereas TBI causes persistent motor dysfunction without positive dynamics [34].

To study vestibular system impairments, the beam balance test is used. This test evaluates static balance. Analysis of these parameters allows conclusions about changes in motor coordination in experimental models [121]. Assessment is conducted using a 6-point scale: low scores correspond to normal function, high scores to impaired balance when traversing the beam. In the beam balance test, higher scores in the TBI group indicate impairment of dynamic balance as a result of TBI.

Comparison of sham and naïve groups showed an increase in mean scores in sham during the first days and at 12 months post-surgery [34]. Similar findings in another study confirmed balance impairments in TBI animals. Persistent changes in vestibular system function in TBI rodents are observed due to disrupted balance-maintaining systems and cerebellar damage after injury [122]. In the sham group, difficulties in beam balance are observed in the first days after surgery and after 12 months. Perioperative factors likely cause transient impairments of static balance, partially compensated over time. Later deterioration may be associated with inflammation affecting coordination areas [41].

Beam walk is a test for assessing dynamic balance to predict falls. It is sensitive to impairments of the vestibular system, cerebellum, and motor cortex. The higher the score, the more pronounced the impairment of dynamic balance. High values in TBI indicate severe impairments, whereas the trepanation procedure causes less pronounced changes due to lesser brain damage [34].

The rotarod test is used to assess motor coordination and balance and is sensitive to cerebellar damage [123]. When evaluating the motor coordination parameter, the values of latency to fall were analyzed. As a result, no significant differences were found between the experimental groups. It is possible that, in this study, the injury did not affect the brain regions responsible for coordination and balance [6]. In another study examining the rotarod test, an increase in the rotarod speed parameter was observed on the 14th day after injury in the TBI group compared to the sham group. These results were obtained because the assessment of locomotor function was performed on days 14 and 30 after injury. Over time, recovery of impairments caused by TBI may have occurred [23]. In turn, deterioration in motor function performance is usually detected on the first day after brain injury [124].

To assess motor function, other tests based on gait analysis can also be used. The foot fault test is sensitive to CNS injuries because gait adaptation on complex surfaces involves information processing in the cerebral cortex. Therefore, brain damage leads to impaired ability to adapt walking to challenging environmental conditions [125]. The assessment of motor function using the foot fault test is based on counting paw slips while traversing a metal grid. In sham, more errors of the hind paws are observed compared to naïve, predominantly for the rear right paw—contralateral to the trepanation site. This is related to signal conduction along the corticospinal tract: damage to one hemisphere causes impairments on the opposite side. In TBI, slipping of both hind paws is increased. The presence of errors in the foot fault test in the absence of changes in gait analysis indicates that the impairments are more likely related to coordination rather than a sensorimotor deficit [80].

The ladder rung test is used for gait analysis and allows for simultaneous assessment of forelimb and hindlimb function [126]. The absence of significant differences between the examined groups during this test confirms the lack of a significant impact of injury on sensorimotor parameters sensitive to this test [6].

Neurobehavioral tests represent a set of tests used to assess sensorimotor task, proprioception, and exploratory behavior in the home cage. When comparing the study parameters before TBI and trepanation and after, a temporary impairment of motor activity was revealed as a result of brain edema following TBI [14]. In this study, when analyzing neurobehavioral scores (NBS), the higher the score, the more severe the dysfunction. Significant differences were identified between the TBI vs. naïve and TBI vs. sham groups throughout the entire study period. However, an increase in test scores between sham and naïve was observed only at 24 and 72 h. Therefore, TBI causes long-term impairment of sensorimotor functions, while trepanation leads to temporary consequences [97]. Thus, perioperative factors themselves may serve as a cause of negative outcomes due to their influence on the brain during surgery. As a result of trepanation, inflammatory mediators are released, leading to BBB disruption, which indicates the development of brain edema [127].

Neurodevelopmental tests are used to study the development of the nervous system. For this purpose, tests are conducted to evaluate somatic development, the appearance of neurological reflexes, and coordination [128]. When assessing normal gait, Δ cliff avoidance and hopping, the values are higher in the TBI group compared to naïve and sham (TBI vs. naïve, TBI vs. sham). For head elevation and cessation of falling, an increase is observed only in TBI vs. naïve. TBI significantly impairs coordination and proprioception, without causing pronounced impairments in somatic development [14].

Neurological severity scores (NSS) represent a method for evaluating the neurological status of animals through the assessment of motor, sensory, reflexes, beam walking, and beam balancing [129]. During this test, it was found that both TBI and trepanation cause impairments in sensorimotor parameters. However, head injury results in more severe consequences than surgical intervention [97]. The results in another study revealed an increased NSS over the course of one month in the TBI group compared to sham and naïve. This indicates that the level of neurological deficit was exclusively associated with TBI [85].

The purpose of the grip strength test is to evaluate the motor and muscular functions of the limbs by measuring the grip strength of rodents. Following TBI and trepanation, a weakening of grip strength was observed [6]. This effect may be due to motor cortex damage, which leads to a decrease in muscle control and strength [130].

In mice after TBI, a pronounced increase in fear in response to sensory stimuli is observed [34]. The data demonstrate an association between TBI and impairments of sensorimotor function, including responses to sound and movement as triggers of fear. In prepulse + pulse trials/startle procedure, sensory sensitivity is reduced in TBI, indicating neuronal dysfunction and weakening of stimulus transmission pathways. In the acoustic startle procedure, reduced reactivity is also observed in TBI. No changes are observed in sham, indicating no effect of trepanation on auditory and tactile pathways. Therefore, the reduced reactivity in TBI is associated with damage to neural circuits rather than surgical stress [131] (Table S7).

Taken together, the results show that behavioral phenotypes after TBI reflect the involvement of specific neural networks: damage to the dorsal hippocampus impairs performance in MWM/RWM/BM and NORT; involvement of the amygdala disrupts fear and its extinction; damage to the sensorimotor cortex, cerebellum, and vestibular structures impairs balance and coordination; disturbances of thalamocortical–hypothalamic loops alter sleep; diffuse axonal injury reduces social functions and sensory filtering. Perioperative factors induce limited behavioral changes that are important to distinguish from the effects of TBI. Therefore, comparisons of TBI vs. sham, TBI vs. naïve, and sham vs. naïve are necessary for the correct interpretation of the results.

## 5. The Effect of Craniotomy on Brain Metabolic Parameters in Traumatic Brain Injury Modeling

The use of protein biomarkers in traumatic damage resulting from TBI is determined by the need for early diagnosis, assessment of injury severity, evaluation of complication risks, and outcome prediction. At present, there are various protein biomarkers capable of reflecting the development of secondary injuries as a result of TBI. Changes in the concentration levels of different proteins are caused by various processes: damage to neuronal cell bodies, axons, and dendrites, astrocyte reactivation, myelin sheath disruption, and microglial activation [132]. In a recently conducted bioinformatic study, the main markers associated with TBI were identified in the following order of significance: BDNF > GFAP > APOE > S100B > MBP > AQP4 > UCHL1 > COMT > DRD2> CACNA1A [133]. Most of these markers have been examined, including in comparisons between naïve and sham groups.

### 5.1. Brain-Derived Neurotrophic Factor (BDNF)

Brain-derived neurotrophic factor (BDNF) plays an important role in synaptic plasticity, learning and memory processes, and also participates in axonal growth of neurons [134,135]. Decreased BDNF transcription in the medial prefrontal cortex was observed in both the TBI group (exons 1 and 4) and the sham group (exon 1). This change correlated with impaired long-term extinction in these groups. Changes in the ipsi-parietal lobe were more variable, while they were absent in the contra-parietal lobe [37]. On the other hand, in a recent study on day 3 after injury, no significant differences were found in relative normalized expression for BDNF in the ipsilateral cortex among all three studied groups [5]. Results of the study of BDNF mRNA levels in the hippocampus indicate a long-term decrease in BDNF expression following both traumatic injury and trepanation, and a short-term increase on the 3rd day after injury, apparently serving as a manifestation of the body’s compensatory action in response to injury [136]. A reduction in BDNF levels in the ipsilateral cortex—observed following both trepanation and TBI—indicates a suppression of neurotrophic support and neuroplasticity processes. Notably, the surgical intervention itself contributes to this decline [137]. These results indicate a long-term decrease in BDNF expression due to both traumatic injury and trepanation, while the short-term increase on day 3 after injury apparently served as a compensatory response of the organism to trauma. During neuroinflammation, which occurs in both TBI and trepanation, microglia become activated. As a result, pro-inflammatory cytokines are released [138], which in turn lead to the suppression of BDNF [139]. Therefore, a low level of BDNF mRNA may serve as a marker of developing neuroinflammation resulting from traumatic injury as well as trepanation (Table S8).

Tropomyosin-related kinase B (TrkB) is a protein involved in the BDNF-TrkB system that mediates neuronal survival and plasticity. The downregulation of the *TrkB* gene specifically following TBI—but not trepanation—indicates an impairment of neurotrophic support and neuronal plasticity resulting from the traumatic injury itself rather than the surgical procedure [137] (Table S8).

### 5.2. Glial Fibrillary Acidic Protein (GFAP)

Glial fibrillary acidic protein (GFAP) is the main structural protein of astroglial cells in the white and gray matter. An increase in GFAP levels in biological fluids occurs due to the disruption of glial cell integrity as a result of TBI. This protein serves as an indicator of BBB damage [140]. A pathological increase in GFAP cell bodies in the hippocampus in the TBI group indicates replacement of damaged tissue with connective tissue and is accompanied by a loss of long-term potentiation (LTP). Surgery did not cause sufficient damage to elicit a reactive astrocyte response [48]. GFAP expression in the primary visual cortex (V1) was increased in the TBI group compared to naive and sham. A persistent increase in GFAP 3 months after injury indicates persistent astrogliosis [38]. Elevated serum GFAP levels in the TBI group reflect the acute phase due to physical impact on the brain [23]. Using the RPPM method, it was found that surgery also had no effect on the integrity of neurons and glia, unlike TBI [141]. Gene expression analysis revealed an increase in the Relative Normalized Expression index only in the TBI group compared to the naive group [5,78,79]. These results also confirm that the observed changes in GFAP expression were caused specifically by mechanical damage and not by the surgical procedure itself.

However, in a number of studies, increased GFAP immunoreactivity in the somatosensory cortex was observed in mice undergoing trepanation, indicating the development of gliosis. However, this indicator was higher in the TBI group than in the sham group, suggesting more extensive glial changes following traumatic injury [142]. Astrogliosis is confirmed by an increase in the GFAP-positive area and gene expression in the ipsilateral cortex and thalamus, and it is triggered by the surgical procedure itself. TBI elicits a more pronounced response, and the rise in serum GFAP reflects the systemic nature of the astroglial response [137]. In the ipsilateral CA3 region, increased GFAP cell levels were observed in both the TBI and sham groups. However, the effect of traumatic injury was greater than that of trepanation [6] (Table S8).

### 5.3. S100 Calcium-Binding Protein B (S100B)

S100B is a calcium-binding protein found in astrocytes and other glial cells. This protein participates in the regulation of protein phosphorylation, enzyme activity, cytoskeletal components, and in maintaining calcium homeostasis in the body. As a result of TBI, S100B penetrates into the bloodstream, being released from damaged glial cells. An increase in the concentration of this protein in blood indicates the presence of injury-induced damage [143]. An increase in this indicator was observed in plasma in sham and TBI groups compared to naïve [144]. The elevation of S100 protein levels—observed in both trepanation and TBI, with higher values in TBI—indicates a pronounced inflammatory response that is triggered by the surgical intervention itself and exacerbated by the trauma [137]. Therefore, high S100B levels indicate astrocyte damage and BBB disruption in both TBI and trepanation. This protein is a sensitive marker for even minimal astrocyte damage that occurs during surgical procedures [145]. At the same time, plasma levels of S100B were decreased in sham and TBI compared to naïve on day 2 after injury, but by day 7 its level normalized. Possibly, these results are due to the fact that the experiment was not conducted within the first hours after injury. Therefore, S100B levels on day 2 could have decreased as a result of natural physiological processes [141] (Table S8).

### 5.4. Myelin Basic Protein (MBP)

Myelin basic protein (MBP) is a key structural component of the myelin sheath, which surrounds nerve cell processes to increase the speed of axonal impulse conduction. Following TBI, the level of intracellular calcium increases, which causes the activation of calpains. Under the action of proteases, MBP degradation occurs. This leads to demyelination and axonal destruction. Thus, the concentration of MBP increases in biological fluids after traumatic injury [132]. An increase in serum MBP levels following both TBI and trepanation may indicate an inflammatory process that develops as a result of the surgical intervention itself and is exacerbated by the trauma [137]. MBP density in areas of the hippocampus, thalamus and amygdala was reduced in TBI compared to sham. Consequently, the decrease in MBP density in TBI in the contralateral corpus callosum and retrosplenial cortex indicates the occurrence of cognitive impairments after injury. Low MBP density results in the hippocampus, thalamus and amygdala following TBI also indicate impairments in memory, emotional stability, and motor activity. In turn, craniotomy did not have a significant effect on the state of the myelin sheath of nerve fibers. Accordingly, the MBP density in sham remained within the normal range [37] (Table S8).

### 5.5. The Role of Craniotomy in Protein Misfolding and Aggregation in Modeling TBI

Brain injury triggers pathophysiological processes that can continue after the acute stage. The long-term degeneration characteristic of TBI consequences is often similar to the processes observed in the development of Alzheimer’s disease; in particular, they are associated with the aggregation and accumulation of misfolded proteins, such as beta-amyloid and tau protein [146]. It is known that after injury, accumulation of Aβ peptides occurs. The main cause of beta-amyloid plaque production is DAI. Disruption of axonal transport occurs, leading to the accumulation of amyloid precursor protein (APP), which is cleaved to form Aβ. Elevated levels of beta-amyloid reduce glutamatergic synaptic plasticity [147,148]. Impairment of LTP, dependent on NMDA receptors, is associated with the accumulation of soluble beta-amyloid oligomers in the brain. An increase in β-APP deposition was detected only in the TBI group across all brain structures examined. The lack of differences between the sham and naive groups confirms the low risk of white matter damage and DAI following surgery [35].

As a result of brain injury, hyperphosphorylated tau protein accumulates due to an increase in intracellular Ca^2+^ with the participation of GSK3ß kinase. Hyperphosphorylated tau proteins interact with normal tau proteins and form neurofibrillary tangles, which lead to memory impairment [149,150]. Histological studies showed that the P-tau area/total cell ratio was increased in the sham and TBI groups compared to the naive group [27]. The increased P-tau concentration is likely due to surgical intervention causing neuroinflammation and glial activation, which promotes the deposition of phosphorylated tau in the hippocampus [151]. Immunohistochemistry revealed increased expression of pTAU-Ser199/202-ir in the sham and TBI groups compared to naïve in both ipsilateral and contralateral areas [152]. Meanwhile, the level of pTAU-Thr231 in plasma was, on the contrary, decreased in sham and TBI compared to Naïve on day 2 after injury. On day 7, this indicator was normalized in all groups [141]. Thus, the TAU-protein response, unlike Aβ, occurs already during surgical intervention at the craniotomy stage in the Sham group, whereas the cortical impact itself does not lead to significant changes.

A uniform distribution of APP was found in the gray matter of the naive group. Brain sections from the TBI group revealed accumulation of APP in axonal blebs in the cortex, hippocampus, and nucleus caudatus-putamen [43], indicating impaired axonal transport [153] (Table S9).

### 5.6. Other Protein Biomarkers of TBI Associated with Various Pathophysiological Processes in TBI

Ubiquitin C-terminal hydrolase L1 (UCHL1) is a protein found in high amounts in neurons and is involved in the regulation of intracellular proteins. During traumatic brain injury, UCHL1 levels increase, indicating disruption of neuronal and BBB integrity [154]. Plasma levels of UCHL1 were elevated in the TBI group compared to the sham group only on day 2 post-injury; no differences were found in the other comparisons. Perioperative factors did not result in significant disruption of the BBB [141].

Reactive astrogliosis is accompanied by increased expression of the gene encoding vimentin, a cytoskeletal protein. The vimentin-positive area in both the ipsilateral cortex and the thalamus was elevated in the sham and TBI groups, with the value in the TBI group exceeding that observed after the surgical procedure alone. However, analysis of gene expression and protein levels revealed an increase in the TBI group compared to both the sham and control groups. While vimentin is involved in reactive astrogliosis following surgery, TBI further enhances its expression, confirming a more pronounced astroglial response to the injury [137].

Microtubule-associated protein 2 (MAP2) is a protein found in the cell bodies and dendrites of neurons and is involved in cytoskeletal stabilization, being a major component of neuronal microtubules. In TBI, damage to axonal microtubules leads to the release and proteolysis of MAP2, which is accompanied by a decrease in its expression in damaged brain areas and an increase in expression in the blood. Furthermore, MAP2 is associated with secondary ischemic injury following TBI [155,156]. Decreased MAP2 immunoreactivity was detected in the hippocampus only after TBI, indicating neurocognitive impairment. Trepanation, however, does not cause decreased MAP2 immunoreactivity and does not lead to secondary ischemia or massive neuronal damage [157].

Ionized calcium-binding adapter molecule 1 (IBA1) is a protein widely used as a marker of microglial activation. In the cerebral cortex, the percentage of Iba-1-positive area increased following both trepanation and TBI compared to controls; notably, the level was higher after TBI than after trepanation. In the thymus, an increase in this parameter was observed only after TBI. TBI induces more pronounced microglial activation in the brain than trepanation does. In the thymus, a similar response develops exclusively in response to TBI, indicating the systemic nature of the inflammatory response to the injury [158]. However, the increase in %IBA1 after 0.5 months exclusively in TBI suggests a direct link between microglial activation in the late post-traumatic period and traumatic brain injury, not trepanation [38].

Complement component 3 (C3) is considered a central component of the complement system and an important pro-inflammatory molecule. Expression of the gene encoding it is elevated in the cortex following both trepanation and TBI, though levels are higher in the TBI group. No increase in expression was observed in the thalamus of the sham group. While a C3-mediated inflammatory response is triggered in the cortex by the craniotomy itself, TBI intensifies this response. The thalamus is involved only in cases of TBI, indicating more widespread inflammation associated with the injury [137].

Peroxisome Proliferator-Activated Receptor α (PPAR-α) and Peroxisome Proliferator-Activated Receptor γ (PPAR-γ) are two isoforms of peroxisome proliferator-activated receptors. The reduction in PPAR-α and PPAR-γ levels in the ipsilateral cortex and thalamus following both trepanation and TBI indicates a suppression of anti-inflammatory and neuroprotective functions, with the surgical intervention itself contributing to this decline [137].

Cathepsin B (CatB) is a lysosomal cysteine protease involved in protein degradation, autophagy, and the regulation of apoptosis. One day after surgery, CatB levels in the brain were reduced in the sham group compared to intact controls. This likely reflects a transient response to trepanation, with local suppression of lysosomal activity, redistribution of proteases in response to mechanical tissue damage, and activation of inflammatory signals. The lack of differences between sham and TBI indicates that TBI does not cause additional changes in CatB in the first 24 h beyond those induced by the surgery itself [159].

Dynorphin A (Dyn A) is an endogenous opioid peptide that modulates pain signal transmission, neuroplasticity, and stress responses. Results of a study of Dyn A levels in the brain structures examined showed that both injury and surgery can transiently reduce Dyn A synthesis or release [160].

The lack of differences in Olig2^+^ cell numbers indicates that neither surgery nor TBI in this experiment significantly affected the oligodendrocyte progenitor population in the brain region examined [35].

Hyaluronic acid (HA) acts as a protective scaffold present in the intercellular space of the brain and participates in the processes of cell differentiation, migration, proliferation, and angiogenesis [85]. Disruption of HA metabolism leads to the development of pathological changes. A study was conducted to investigate the effect of TBI on the expression of genes responsible for the synthesis and degradation of HA, as well as HA receptor genes. In TBI, increased expression of HAS1 and RHAMM was observed on day 3, HAS2 and HYAL1 on days 1 and 3, and CD44 at all time points. In the sham group, an increase in HAS1 was noted at 4 h and on day 1 after trepanation relative to naive [161]. The increased expression of hyaluronan synthase genes in animals with brain injury indicates enhanced HA synthesis, which reflects a pathological response to tissue damage. The elevated CD44 expression in the TBI group serves as a marker of inflammatory processes in the lesion area [162]. In the Sham group, the observed increase in HAS1 expression within 24 h after trepanation indicates the activation of inflammatory and reparative processes in response to tissue damage caused by surgical intervention [161].

GLUT4 ensures the transport of glucose into the cell. In TBI, *Glut4* expression is reduced relative to control in both the cerebral cortex and hippocampus. There was a decrease in the expression of the enzyme of the first stage of glycolysis, Hexokinase 1 (*Hk-1*), in the TBI group compared to sham in the cerebral cortex. This indicates a disruption of insulin-dependent glucose transport and may aggravate post-traumatic energy deficit of neurons [78].

Cluster of differentiation 68 (CD68) serves as a marker for macrophages and monocytes. Both trepanation and TBI lead to an increase in CD68-positive macrophages in the cerebral cortex, with levels being higher following TBI than after surgical intervention. In the thymus, an increase in CD68-positive macrophages is observed only in cases of traumatic injury. Notably, *Cd68* expression is elevated in both brain structures following both trepanation and TBI. Surgical intervention alone triggers a macrophage response in the brain, whereas TBI intensifies this response and additionally involves the thymus, indicating the systemic nature of the immune response to trauma. An increase in cluster of differentiation 206 (*Cd206*) expression was observed only in the TBI group compared to the control and sham groups. Specifically, TBI selectively triggers M2 macrophage polarization, whereas surgical intervention alone does not elicit such a response. This is corroborated by similar findings regarding arginase 1 (*Arg-1*) expression, a marker actively expressed by macrophages during M2 polarization. [158].

The intrinsic apoptotic pathway is regulated by the balance between pro- and anti-apoptotic proteins of the B-cell lymphoma 2 (Bcl-2) family. Bcl-2-associated death promoter (Bad) neutralizes the anti-apoptotic protein Bcl-2, thereby allowing Bcl-2-associated X protein (Bax) to oligomerize on mitochondria and trigger the release of cytochrome c, which activates caspase-9. Bcl-2 inhibits apoptosis, Bad initiates it, and caspase-9 executes cell death [163]. Experimental data indicate that the Bcl-2-positive area is increased in the ipsilateral cortex in both sham and TBI groups compared to controls, with levels being higher in the TBI group than in the sham group. In the thalamus, this parameter is elevated only in the TBI group [137]. However, levels of the pro-apoptotic proteins Bax and Bad are also increased in the cortex following both craniotomy and TBI, whereas in the thalamus, this increase occurs only after TBI [158]. Casp9 expression is elevated in the cortex following TBI [78]. Surgical intervention alone can trigger both anti- and pro-apoptotic responses in the cortex, whereas TBI intensifies these processes and extends them to the thalamus, indicating a more extensive and generalized activation of apoptosis following injury.

Secondary injuries after TBI lead to membrane depolarization. Excessive activation of NMDA receptors increases Ca^2+^ influx. High concentrations of Ca^2+^ result in overactivation of calpains. The elevated activation of these cysteine proteases leads to neuronal damage by disrupting cytoskeletal structure [164]. NF-68 and NF-200 are filaments that are part of the neuronal cytoskeleton and can undergo calpain-mediated degradation [153]. Both surgery and traumatic injury are accompanied by an increase in intracellular calcium and calpain activation. This is confirmed by the accumulation of spectrin breakdown products (BDPs) in the SHA and TBI groups. Moreover, TBI causes a more pronounced loss of neurofilament proteins (NF68, NF200), especially in the ipsilateral cortex, reflecting the severity of pathological changes [165].

There is an opinion that protein markers are not sufficiently sensitive to mild TBI. This phenomenon is explained by the fact that, in mild cases, the BBB remains impermeable to large molecules that could indicate tissue damage. In turn, the use of lipids as TBI markers offers several advantages. Lipids are small molecules; therefore, they are capable of crossing the BBB even with minor damage. Additionally, the development of oxidative stress in the brain leads to the formation of lipid peroxidation products (LPO), which can also serve as indicators of TBI. The application of a statistical method made it possible to determine that the observed changes in lipid composition were caused solely by TBI. This allowed the exclusion of surgical intervention as a factor influencing the obtained results [166] (Table S8).

### 5.7. The Effect of Traumatic Injury Due to TBI on the Level of Hormones and Amino Acid Metabolites

In a study of rabbit pups, surgery in the Sham group had no effect on tryptophan metabolism compared to intact animals. In the trauma group, persistent and specific abnormalities were observed. In animals with TBI, decreased serotonin/tryptophan and melatonin/tryptophan ratios were observed on day 21, indicating impaired conversion of tryptophan to serotonin and melatonin. Absolute melatonin levels were also significantly reduced in the trauma group on day 21. Furthermore, shortening of serotonin fibers was observed in the injury zone. This indicates structural damage to the serotonergic system. These data demonstrate that it is the trauma, and not the surgical stress, that leads to a long-term redistribution of tryptophan metabolism with suppression of the serotonin-melatonin pathway and structural damage, which can have serious consequences for neurodevelopment and recovery [7] (Table S8).

### 5.8. The Effect of Traumatic Injury Due to TBI on Mitochondrial Dysfunctions

TBI causes serious mitochondrial dysfunctions, among which the most important are impaired mitochondrial bioenergetics associated with decreased ATP production and hyperproduction of ROS, as well as the triggering of apoptotic processes, which are closely related to mitochondrial functionality [167]. Mitochondrial DNA (mtDNA) is a circular molecule encoding a number of respiratory chain proteins and is involved in the regulation of oxidative phosphorylation. The normalized number of mtDNA copies relative to nuclear DNA (mtDNA/nDNA) serves as a marker of the functional state of mitochondria. A decrease in this indicator reflects damage to the mitochondrial apparatus and disruption of bioenergetic processes in brain cells [168]. In the first hours after TBI, the expression of cytochrome c oxidase I, II, and III, which are mtDNA-encoded subunits of complex IV, decreases. In turn, surgery did not affect the expression of mtDNA-encoded cytochrome c oxidase subunits [169] or the mitochondrial pool. In TBI, mtDNA/nDNA was reduced relative to naive [5]. A decrease in copy number during TBI in the hippocampus relative to the control and in the cortex relative to the control and sham groups indicates inhibition of mitochondrial biogenesis and a decrease in the mitochondrial pool [78]. Damage to mtDNA is also a serious cause of mitochondrial dysfunction, which disrupts the synthesis of respiratory chain proteins and impairs energy metabolism in neurons. Quantitative assessment of the degree of mtDNA damage using long-fragment PCR allows detection of the level of mitochondrial stress in the tissue [170]. It was found that sham and TBI groups had a significantly increased number of mtDNA damages 3 days after injury, while no differences were found between the sham and TBI groups [5]. Mitochondrial damage can also lead to the release of mtDNA from the cell into the blood plasma. It was shown that the level of freely circulating mtDNA was significantly increased after injury compared to both naïve and sham mice [79]. Thus, these studies demonstrate that mtDNA is a sufficiently sensitive component of mitochondria. Damage to mtDNA occurs after craniotomy, whereas more dramatic changes in mitochondrial functionality, expressed as a decrease in mtDNA content and its release into the blood plasma, occur only after the impactor strike.

Translocator protein (TSPO) is located in the outer mitochondrial membrane and is involved in the transport of compounds across it. It is often used as a marker of cellular metabolic depression and neuroinflammation. The sham operation did not lead to a decrease in TSPO, whereas the inflicted injury led to a decrease in its level [14].

Transcription factor A, mitochondrial (TFAM), is a key regulator of mitochondrial biogenesis. In the cerebral cortex, *Tfam* expression is increased in the sham group relative to the control, while in TBI it is decreased compared to sham. In the hippocampus, on the contrary, the level of *Tfam* expression in TBI was lower than control values. TBI is accompanied by region-specific impairment of mitochondrial biogenesis. Surgical exposure induces compensatory-adaptive activation of mitochondrial biogenesis [78].

PTEN-induced kinase 1 (PINK1) is involved in mitochondrial quality control and the initiation of mitophagy. Increased expression in the hippocampus in TBI relative to sham, reflecting compensatory activation of the mitophagic response to traumatic injury [78] (Table S10).

### 5.9. The Role of Craniotomy in TBI-Induced Alterations in Blood microRNA

MicroRNAs (miRNAs) are a class of small non-coding RNAs, typically 19–22 nucleotides in length. It is well known that microRNAs play an important role in maintaining and regulating physiological functions. In recent years, expression studies have shown that certain microRNAs possess diagnostic and prognostic significance in TBI [171]. The expression levels of miR-9a-3p, miR-136-3p, miR-434-3p, miR-323-3p, miR-212-3p, and miR-132-3p in blood plasma increased after trepanation, but to a greater extent after TBI [172,173]. Similar data were obtained for miR-323-3p, miR-212-3p, and miR-132-3p. In contrast, miR-124-3p increased only in the TBI group, without any change in sham [173] (Table S11).

Taken together, these data demonstrate that the markers differ in their specificity to injury and sensitivity to craniotomy. Persistent local GFAP, decreased MAP2 in the hippocampus, increased UCHL1 on the second day, decreased TSPO, systemic extracellular mtDNA, as well as accumulation of APP and Aβ, predominantly reflect traumatic damage. S100B in the early hours, p-tau in several protocols, and tissue damage to mtDNA are sensitive to surgery and therefore require the mandatory inclusion of a naïve group and precise fixation of time windows. The regional specificity and compartmentalization of signals, as well as differences in kinetics during the first hours and days after interventions, dictate the necessity of standardizing measurement and reporting protocols with parallel comparisons of TBI vs. sham, TBI vs. naïve, and sham vs. naïve.

## 6. The Role of Craniotomy in Inflammatory Processes During Traumatic Brain Injury Modeling

As a result of nerve tissue damage in TBI, neuroinflammation develops, beginning with the activation of microglia and their transition into a reactive state. Reactive microglia acquire macrophage-like properties. As a result of microglial polarization, two types of cells are formed. The M1 phenotype produces pro-inflammatory cytokines such as tumor necrosis factor-alpha (TNF-α), interleukin-6 (IL-6), and interleukin-1β (IL-1β). M1-mediated cytokine production contributes to the increased permeability of the BBB. In turn, the M2 phenotype participates in the production of anti-inflammatory cytokines [174]. Measurement of cytokine expression levels released after brain injury serves as a marker of tissue damage severity [156].

### 6.1. Interleukins

IL-1α belongs to the family of pro-inflammatory cytokines, which are important mediators of the innate immune response. Neuronal death resulting from TBI is caused by the transmission of molecular signals through IL-1α from central and peripheral inflammatory processes [175]. The plasma IL-1α level did not change in the experimental groups on the 3rd day after the injury [5].

IL-1β in TBI is produced predominantly by reactive microglia; however, it can also be synthesized by neurons, astrocytes, and oligodendrocytes [176]. Mature IL-1β enhances the expression of adhesion molecules, chemokines, and other cytokines by activating the NF-κB and MAPK cascades. In the central nervous system, IL-1β participates in the regulation of neuroinflammation, where its excessive production after trauma or ischemia may contribute to secondary tissue damage [177]. IL-1β plays an important role in the release of other cytokines during inflammation. The serum IL-1β level usually increases after brain injury [178]. The level of IL-1β mRNA expression did not differ between naïve and sham, whereas in the TBI group its expression in the injury zones was increased at all time points studied [7]. In the blood, on the 3rd day after injury, no changes in IL-1β expression level were observed between all three experimental groups [5]. The increase in IL-1β levels in the cerebral cortex and thalamus following both trepanation and TBI indicates that the surgical intervention itself triggers an inflammatory response [158].

IL-4 is a key anti-inflammatory mediator that regulates the Th1/Th2 response balance and activates anti-inflammatory mechanisms through the JAK1/3–STAT6 signaling pathway. It stimulates macrophage polarization toward the M2 phenotype, promotes the production of antagonists of pro-inflammatory cytokines, and supports reparative processes [179]. IL-4 expression was increased in the TBI group compared with sham only at postoperative day 7 [7].

IL-6 plays an important role in both the formation of inflammation and the process of tissue regeneration. This cytokine is produced by microglia, neurons, and endothelial cells [180]. Clinical studies have demonstrated that IL-6 levels increase in the serum of patients in the early stages after TBI and may serve as a predictor of mortality [181]. An increase in IL-6 levels following trepanation was observed only in the cerebral cortex, whereas in cases of TBI, it occurred in both the cortex and the thalamus. Surgical intervention itself triggers a local inflammatory response, while traumatic injury intensifies this response and extends it to the thalamus [158]. In rabbits, Il-6 expression in the damaged brain region after TBI was consistently higher than in other groups [7]. The protein cardiotrophin-like cytokine factor 1 (CLCF1) belongs to the IL-6 family of cytokines. It exerts a neurotrophic effect and supports neuronal survival. Overexpression of the *Clcf1* gene exclusively in the TBI group indicates the activation of a compensatory neurotrophic response aimed at maintaining neuronal survival following traumatic injury, whereas craniotomy alone does not elicit such a response [137].

IL-10 is a key mediator of the anti-inflammatory response, acting through the activation of JAK1/TYK2 and the transcription factor STAT3, which induces the expression of SOCS3 and other inhibitors of inflammatory cascades. This leads to the suppression of NF-κB-dependent transcription of pro-inflammatory cytokines, including TNF-α, IL-1β, and IL-6 [182]. In the TBI group, a decrease in IL-10 protein was detected at day 7, despite a stable increase in gene expression levels until day 21, which may indicate translational disturbances [7] (Table S12).

### 6.2. Tumor Necrosis Factor A

TNF-α is a multifunctional cytokine belonging to the TNF ligand superfamily. The main sources of TNF-α are microglia, which are activated immediately after injury, as well as astrocytes, neurons, and infiltrating immune cells that penetrate the brain following disruption of the BBB. A positive correlation has been noted between TNF-α expression levels and the degree of neurological impairment resulting from TBI [183]. In addition, TNF-α promoted oxidative stress by activating downstream NF-κB/iNOS signaling, which led to pericyte-mediated disturbance of cerebral microcirculation [184]. The elevation of TNFα levels in the cerebral cortex and thalamus following both trepanation and TBI —with a more pronounced increase in the case of injury—indicates that surgical intervention itself triggers a proinflammatory response that is significantly intensified by traumatic injury. [158]. In another study, perioperative factors caused a transient increase in TNF-α expression in sham mice after 6 h, but these levels returned to normal by 24 h. In TBI, TNF-α gene expression was elevated throughout the entire 21-day period, but protein levels were elevated only on day 7 [7]. Similarly, plasma TNF-α protein levels increased on day 3 only in TBI [79], while TNF-α mRNA levels did not differ significantly between the experimental groups [5]. Thus, unlike interleukins, a transient increase in TNF-α is possible with trepanation, but a sustained increase requires a more severe impact on the brain, such as a cortical stroke (Table S12).

### 6.3. Other Inflammatory Markers

Nuclear factor kappa B (NFκB) plays an important role in regulating the inflammatory response. This transcription factor performs a dual function. On one hand, NFκB contributes to the worsening of the condition following brain injury, while on the other, it exerts a neuroprotective effect [185]. Plasma NFκB concentrations were elevated in the sham and TBI groups compared to the naive group, with no differences between the sham and TBI groups [5]. There was an increase in the expression level in the TBI group compared to the sham group in the cerebral cortex [78]. Therefore, the increase in NFκB reflects an inflammatory response associated not only with TBI but also with prior craniotomy [5].

Prostaglandin-endoperoxide synthase 2 (Ptgs2) is an important enzyme in prostaglandin biosynthesis and serves as a marker of inflammatory processes. Ptgs2 is also involved in the process of ferroptotic cell death, which can occur in response to TBI [186]. Since Ptgs2 expression was increased only between the TBI vs. naïve groups, it can be concluded that its increase is associated with TBI and not with surgical intervention [5].

Cellular and humoral immune responses can be activated as a result of protein alterations that occur following TBI, leading to progressive neuronal death. In B-lymphocytes, inflammatory reactions in the brain activate antibody production. In turn, TBI is expected to cause a reduction in the percentage of T-lymphocytes [187]. Immunohistochemically, IgG antibodies reactive with neurons were detected in the TBI and sham groups. Moreover, their levels were lower after trepanation, and they were not detected at all in the naive group. T cells were not detected in either group. Autoreactive antibodies were present after both TBI and trepanation. It is possible that the presence of anti-brain antibodies could have caused the pathological changes in the brain [13].

Myeloperoxidase activity increased only after TBI, but changed in sham-operated rodents compared to the naïve group. The increase in MPO activity in the brain after injury indicates active neutrophilic inflammation, which contributes to the pathogenesis of secondary brain damage [188] (Table S12).

### 6.4. The Effect of TBI on the Microbiome

Currently, there are data indicating the influence of symbiotic intestinal microbiota on the brain as part of the microbiota–gut–brain (MGB) axis [189]. The microbiome participates in the regulation of important physiological processes such as metabolism, energy homeostasis, maintenance of intestinal epithelial integrity, immune activity, and the development of neurobehavioral functions [190].

Acute TBI disrupts the coordinated functioning of the MGB axis, which includes the brain, gastrointestinal tract, and gut microbiome. Brain injury leads to changes in the composition of the microbiota, which in turn, through bacterial metabolites, influences the severity of TBI, neuroplasticity, and metabolic pathways. Damage to the central nervous system affects the abundance and diversity of bacterial colonies inhabiting the gut [191]. Alteration of gut microbiota composition results in the inability to prevent intestinal inflammation and to restore intestinal barrier permeability via epithelial cell regeneration, thereby contributing to gastrointestinal dysfunction during TBI.

Real-time PCR revealed a trend toward a decrease in the relative abundance of Bacteroidetes in both craniotomy (by 13.1%) and TBI (by 16.9%) compared to naive. However, the differences were not statistically significant. In contrast, the level of Firmicutes increased by 37.9% in the sham group and by 58.7% in the TBI group [5]. It is believed that a shift in the Bacteroidetes/Firmicutes ratio toward Firmicutes may serve as a pro-inflammatory marker. Previously, plasma C-reactive protein levels were shown to negatively correlate with the Bacteroidetes/Firmicutes ratio (rs = −0.41, *p* = 0.03) [192].

Microbiome sequencing revealed that 24 h after surgery, the sham group showed a sharp increase in *Escherichia coli* and *Shigella* spp., to a greater extent than the TBI group. Furthermore, the sham group showed a decrease in the Shannon index, which may indicate a decrease in gut microbiome diversity. After 1.5 months, no significant differences were found in *Lactobacillus* spp., *Bifidobacterium* spp., and *Lachnospiraceae* bacteria. Pseudomonas aeruginosa levels were reduced primarily in the sham group. At 3 months post-injury, the TBI group showed an increase in opportunistic bacteria (*Escherichia coli*, *Streptococcus spp.*, *Shigella* spp., and *Pseudomonas aeruginosa*) and a decrease in *Desulfovibrio* spp., compared with both the naive and sham groups [193]. This effect arises from impaired intestinal barrier function, leading to increased translocation of metabolites and bacteria. This results in dysbiosis, characterized by an elevated concentration of opportunistic microorganisms compared to beneficial bacteria [194]. However, not only TBI but also trepanation led to changes in microbiome composition. Unlike traumatic injury, the surgical procedure caused only a temporary effect on intestinal bacterial levels. It is possible that trepanation disrupted the integrity of the BBB, thereby inducing systemic inflammation. As a result, intestinal epithelial barrier permeability increased, leading to dysbiosis.

Changes in rodent microbiota observed in experimental models are often interpreted as independent factors; however, it is more appropriate to view them as secondary to systemic inflammation, changes in husbandry, stress, and postoperative care protocols. Systemic inflammation alters the composition of the healthy microbial community via immune and metabolic signals; dietary changes provide different substrates for microorganisms; stress affects gastrointestinal motility and the MGB axis; and postoperative care measures—such as the use of antibiotics and analgesics, isolation, and temperature regulation—directly or transiently suppress or shift microbial activity. These factors must be taken into account when interpreting results and planning studies [195,196] (Table S13). While acknowledging the contribution of these secondary factors, one must also consider the direct impact of the trepanation procedure and the TBI itself. The mechanisms underlying these changes differ: trepanation typically causes only a temporary disruption of the BBB and associated systemic inflammation—briefly increasing barrier permeability and leading to transient dysbiosis—whereas TBI triggers a more persistent pathological process characterized by impaired barrier function, enhanced translocation of microbial elements and metabolites, and dysbiosis marked by an overgrowth of opportunistic microflora, particularly in the long term. The mechanisms underlying these changes differ: perioperative factors typically cause only a temporary disruption of the BBB and associated systemic inflammation—briefly increasing barrier permeability and leading to transient dysbiosis—whereas TBI triggers a more persistent pathological process characterized by impaired barrier function, enhanced translocation of microbial elements and metabolites, and dysbiosis marked by an overgrowth of opportunistic microflora, particularly in the long term. These disturbances form the basis of the MGB axis: in the case of trepanation, the signal from the brain to the bone marrow is disrupted, resulting in transient dysbiosis; conversely, in TBI, the initial brain damage triggers a persistent bidirectional cascade—a sustained systemic condition and intestinal barrier dysfunction—which secondarily exacerbates brain pathology, driving a recurring cycle of periodic changes [197].

Thus, trepanation itself is a significant intervention capable of initiating a complex inflammatory response; however, its effects differ qualitatively and quantitatively from the consequences of direct TBI. Perioperative factors may cause a short-term and limited alteration in the cytokine profile, while stable and pronounced neuroinflammation is observed only in groups that have undergone direct injury. An increase in myeloperoxidase activity—a specific marker of neutrophil infiltration—is observed exclusively after TBI, emphasizing its role in triggering acute neutrophilic inflammation, which is not characteristic of trepanation alone. The formation of autoreactive antibodies after both craniotomy and TBI indicates that disruption of the BBB during surgery may already initiate autoimmune mechanisms that could potentially exacerbate long-term outcomes. Both TBI and craniotomy induce dysbiotic changes in the composition of the gut microbiota; however, their nature and dynamics differ. Surgical intervention leads to more abrupt but transient shifts (a surge of conditionally pathogenic bacteria within 24 h), whereas TBI causes persistent long-term alterations. Taken together, these findings suggest that trepanation is not a neutral control but an intervention that elicits its own inflammatory and immune response. This must be taken into account when modeling TBI for proper data interpretation.

## 7. Discussion

The evidence synthesized in this review indicates that the consequences of sham surgery cannot be described by a simple binary distinction between the presence and absence of traumatic brain injury. Across the examined studies, the sham procedure produced an outcome-, region-, and time-dependent biological response. For many structural, electrophysiological, and molecular measures, sham-operated animals remained close to naïve controls, whereas selected neuronal, inflammatory, metabolic, nociceptive, behavioral, and microbiome-related outcomes were measurably altered. These changes were generally more localized, less pronounced, or more transient than those induced by TBI, but they were not uniformly negligible. Importantly, the sham condition should not be interpreted merely as a milder form of TBI: some endpoints changed after both interventions with different magnitudes or kinetics, whereas others were selectively affected either by the surgical procedure or by traumatic impact. The principal implication is therefore that the specificity of an experimental outcome cannot be inferred from the group label alone but must be evaluated in relation to the biological compartment, anatomical region, observation period, injury model, and procedural conditions. This context-dependent interpretation also provides the basis for considering anesthesia as an additional modifier of the observed phenotype and for evaluating the three-group design within a bioethical framework.

Anesthesia is an unavoidable component of most craniotomy-based TBI models and should be considered not only as a potential source of adverse effects but also as a biologically active modifier of secondary brain injury. Experimental evidence indicates that most major classes of general anesthetics, including volatile anesthetics, barbiturates, propofol, and ketamine, can exert neuroprotective effects under specific experimental conditions. These effects have been associated with suppression of excitotoxic signaling, reduction in cerebral metabolic demand and oxidative stress, modulation of inflammatory responses, and alterations in mitochondrial pathways [198,199]. At the same time, anesthetic agents can inhibit mitochondrial respiration, alter redox homeostasis, and induce neuroinflammatory or neurotoxic responses depending on the agent, dose, duration of exposure, developmental stage, and injury context [199,200]. Consequently, anesthesia may confound experimental TBI outcomes in two directions: it may contribute to differences between naïve and sham-operated animals, but its neuroprotective properties may also attenuate secondary injury in the TBI group and thereby reduce the apparent magnitude of TBI-specific effects. Available control data suggest that short-term isoflurane exposure alone does not necessarily alter the examined cognitive and behavioral outcomes [80]. Studies involving anesthesia combined with surgery demonstrated transient microglial activation and increased inflammatory cytokine levels without detectable impairment of spatial reference memory in young adult mice, even after repeated procedures [201,202]. These findings indicate that anesthesia is unlikely to account for the entire pathological signature observed in sham-operated animals; nevertheless, its potentially neuroprotective, metabolic, and immunomodulatory effects should be considered when interpreting comparisons among naïve, sham, and TBI groups. Therefore, the anesthetic agent, concentration, exposure duration, timing relative to injury, physiological monitoring, and perioperative analgesia should be reported in detail, while anesthesia-only controls may be particularly valuable when studying anesthesia-sensitive endpoints.

Following this, the differences arising between the naïve and sham-operated groups cannot be attributed exclusively to the effects exerted by craniotomy. The formation of the measured parameters in rodents that underwent craniotomy is determined by a number of concomitant factors: the type of anesthesia and analgesics used, the degree of brain tissue damage, the technique used to create the cranial opening and its diameter, as well as local overheating and vibration inevitably occurring during bone drilling. Within the framework of a separate study, the effects of using a manual versus an electric drill during craniotomy modeling on the recorded parameters were compared. Animals that underwent craniotomy using an electric drill exhibited more persistent sensorimotor dysfunctions and a higher incidence of seizure manifestations compared with animals in which craniotomy was performed using a manual drill. Thus, the use of an electric drill resulted in more pronounced brain tissue damage, which was reflected in cognitive performance and was accompanied by an increased probability of seizure occurrence [203]. Another study also demonstrated that the use of an electric drill was associated with more pronounced damage than the use of a manual trephine [8]. Another important source of differences between the groups is the selected type of cranial surgery: craniotomy or craniectomy, since these approaches involve different methods for the subsequent closure of the cranial opening. When comparing the effects in groups in which the cranial opening was left open with those in which the integrity of the skull was restored immediately after the intervention, it was found that the presence of an open cranial opening was associated with impaired recovery of motor function [204].

A substantial influence on the phenotype of animals in the sham-operated group may also be exerted by temperature control, which is required to prevent secondary damage developing as a consequence of impaired thermoregulation during the postoperative period. After TBI modeling, the animal’s brain is in an extremely vulnerable state. Therefore, monitoring the animal’s body temperature is of critical importance. TBI can directly damage the hypothalamus and other brain structures responsible for thermoregulation. This may lead to the organism’s reduced ability to maintain its own temperature [205]. After modeling TBI, the animal’s brain is in an extremely vulnerable state. TBI can cause reactive astrogliosis and inflammation in the hypothalamic centers responsible for thermoregulation [206]. As a result, the animal’s body will be poor at maintaining its own temperature. Therefore, monitoring the animal’s body temperature is particularly important. Post-traumatic hyperthermia is a secondary injury that can slow recovery processes. Exposure to high temperatures increases BBB permeability, leading to increased damage to axons and cerebral blood vessels [207]. Preventing the development of post-traumatic hypothermia is also important. In some cases, intentional cooling of the body can have a positive effect on the brain’s condition after injury [208,209]. However, in some cases, anesthesia and hypothermia can reduce the extent of brain damage and enhance the systemic stress response [210]. Therefore, infrared lamps should be used to prevent unwanted temperature reduction. In addition, postoperative stress may also act as a significant factor contributing to the deterioration of the recorded parameters. The persistence of acute pain after surgery under conditions of insufficiently effective analgesia may contribute to behavioral disturbances, thereby affecting the experimental outcomes [211].

To obtain unambiguous conclusions regarding the consequences of craniotomy when comparing data from numerous publications, it is necessary to take into account both the individual characteristics of the animals themselves and the technique and method used to perform the craniotomy and inflict the injury. In different studies, the investigated parameters may vary depending on the following characteristics: species, body weight, age, and sex of the animals. Analysis of general parameters in different rodent species indicates the presence of interspecies differences in the neurological consequences of TBI. Body weight, in turn, serves as an important indicator of the physiological condition of animals after injury. Differences in body weight between individual animals may determine differences in the severity of the resulting injuries [212]. Age of the animal is a factor determining the severity of the consequences following TBI. Aged animals exhibit more pronounced motor and cognitive impairments after traumatic brain injury compared with young animals [213]. The nature of the neurological consequences of TBI may differ substantially depending on the sex of the animals. Following injury, females exhibit behavioral alterations and reduced hippocampal neurogenesis, whereas cognitive changes are less pronounced in males [214].

The analyzed sources also differ with respect to the injury models themselves: the type of model and the severity of TBI, as well as the diameter and location of the cranial opening. The type of injury and the extent of the lesion depend on the TBI model. For example, CCI model is characterized by the development of a localized focal lesion with a confined area of injury. In turn, the FPI model results in diffuse brain injury, leading to widespread impairments [215]. The severity of brain tissue damage is directly correlated with the extent of neurological and motor dysfunction [216]. When it is necessary to investigate functional impairments characteristic of a specific brain region, the cranial opening is positioned directly above that area. Altering the coordinates of the cranial window provides access to other brain structures. This may result in behavioral effects that differ from those observed with the original localization [217].

An additional factor complicating the comparison of parameters across studies is the experimental design itself, since different studies employ different time points and methodologies for assessing the parameters of interest. Therefore, the conclusions formulated on the basis of the present review should be regarded exclusively as established qualitative patterns rather than as a precise quantitative measure of the “effect of craniotomy.” Particular mention should be made of the limited number of studies investigating certain specific parameters, which reduces the statistical validity of the generalizations made. It is also impossible to state unequivocally that the absence of statistically significant differences between the naïve and sham-operated groups constitutes convincing evidence of their complete equivalence. Indeed, the absence of statistically significant differences between groups may be attributable not to the true equivalence of their conditions, but rather to an insufficient sample size or the limited sensitivity of the assessment methods employed.

Beyond their methodological implications, the present findings have a direct bioethical relevance within the 3Rs framework. Reduction cannot be assessed solely by counting the number of animals used in an individual experiment. The sham procedure is itself an invasive intervention and, as shown by the evidence summarized here, may produce pain-related, inflammatory, metabolic, neuronal, and functional alterations. For the question addressed in this review, omission of a naïve reference may therefore leave the biological burden borne by sham-operated and TBI animals only partially interpretable. Under these conditions, inclusion of both control groups does not necessarily represent an unjustified expansion of sample size; rather, it may increase the scientific value of the information obtained from each animal and strengthen the harm–benefit balance of the study. Ethical assessment of craniotomy-based TBI experiments should therefore consider not only animal numbers and procedural severity, but also whether the control-group structure is sufficient to ensure that the imposed harms yield scientifically interpretable information. Within the 3Rs framework, this interpretation links Reduction to an adequately informative experimental design, while Refinement requires explicit recognition and minimization of the welfare burden associated with the sham procedure [218,219,220].

## 8. Materials and Methods

**Figure 5:**
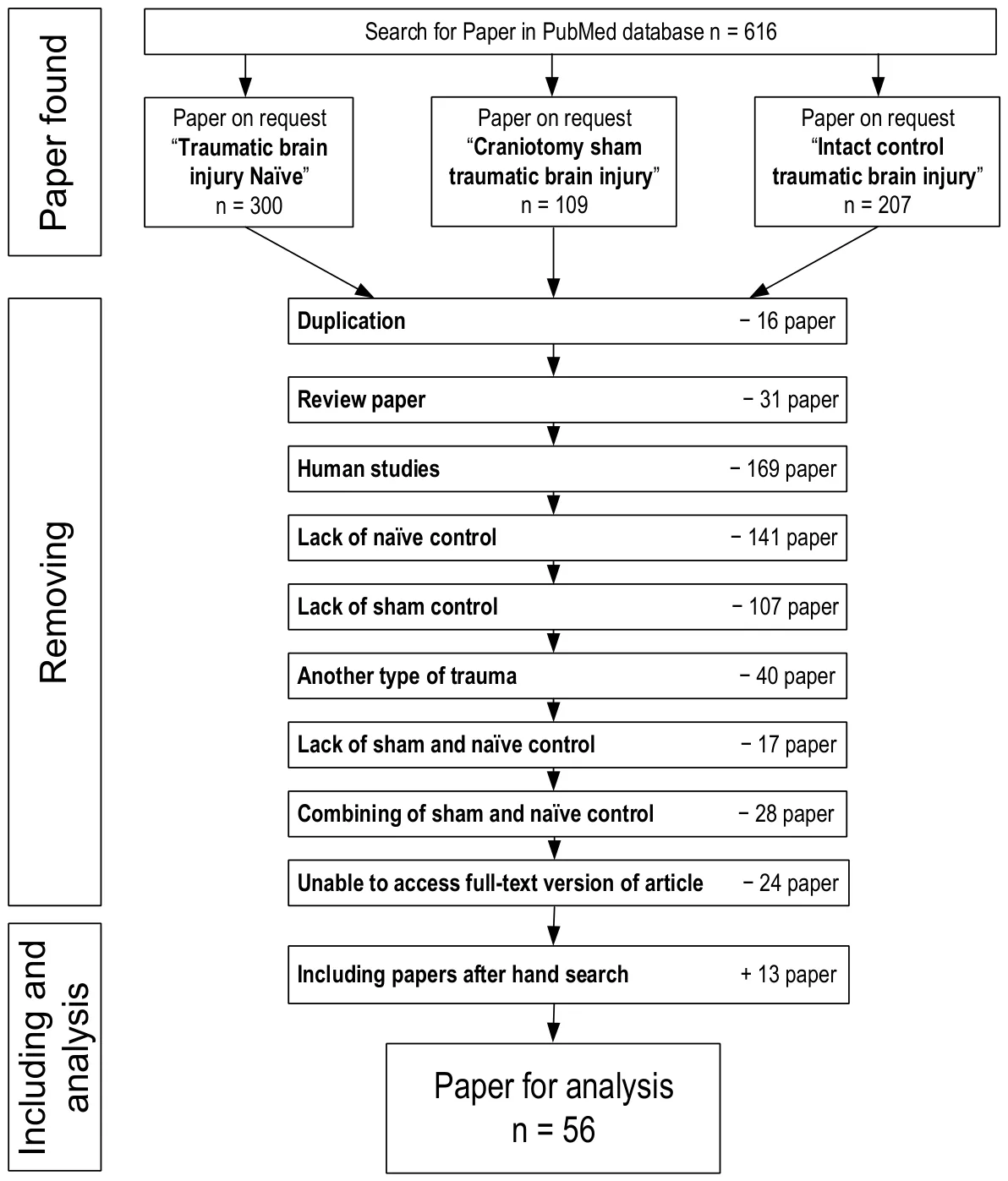
Scheme of the systematic literature search as of 31 March 2025.

A systematic search of scientific literature was conducted through the PubMed database as of 31 March 2025. The search was based on three queries: “Traumatic brain injury naïve,” “Craniotomy sham traumatic brain injury,” and “Intact control traumatic brain injury,” which identified a total of 616 articles. Preliminary analysis showed that these search queries yielded the largest number of studies related to the experimental induction of TBI using a three-group experimental design (naïve/sham/TBI). At the first stage, duplicate articles were excluded (n = 16). Then, review articles (n = 31) and clinical studies involving humans (n = 169) were also excluded from the analysis. A number of studies were excluded because one or more of the groups required for this analysis were missing (either sham, naïve, or both) (n = 265). In 28 articles, the sham and naïve groups were combined into a single group, which made it impossible to perform a comparative analysis between these two experimental groups. In some studies, other types of injuries were investigated; therefore, these were also excluded from the analysis (n = 40). Finally, the authors did not have access to the full-text versions of 24 manuscripts. Although PubMed was used as the primary database for the literature search, the search was supplemented by Web of Science to ensure a more comprehensive identification of relevant studies. Specifically, 13 additional articles were identified through Web of Science, which were subsequently screened and included in the review according to the predefined eligibility criteria. Thus, the literature search was not limited to PubMed, thereby reducing the risk of database-related limitations and improving the comprehensiveness of the literature search. In total, 56 manuscripts meeting the requirement of a three-group experimental design (naïve/sham/TBI) were analyzed (Figure 5). An analysis of the method used to simulate TBI and trepanation was presented (Table S14).

The risk of bias in the majority of the included studies is assessed as predominantly low. Virtually all the studies demonstrate a low risk regarding criteria such as temperature standardization, the craniotomy technique, uniformity of follow-up periods, and issues of funding and conflicts of interest. While an “unclear” assessment appears in some studies regarding specific domains—such as randomization, temperature control, and perioperative care—this does not represent a systemic issue. Overall, the methodological quality of the included studies can be characterized as high, and the overall risk of bias as low, allowing the results to be interpreted with a sufficient degree of confidence (Table S15).

In the analyzed studies, different species and strains of rodents were used, predominantly Sprague–Dawley rats ~41.5% and Wistar rats ~11%, as well as C57BL/6 mice ~28.3%, which also differed in age and body weight. Most studies were conducted on males, whereas females and groups of both sexes were encountered considerably less frequently. Of the 53 selected articles, ~85% used males, while the remaining ~15% consisted of females. At the same time, the CCI model was used in 43.4%, FPI—26.4%, LFPI—13.2%, WDI—7.5%, CHI—3.8%, mFPI—1.9%, PBBI—1.9%, and Ecci—1.9%. Therefore, differences between studies cannot be attributed exclusively to the presence or absence of craniotomy, since animal characteristics and TBI models may also substantially affect the results.

The response to TBI may differ depending on sex. In a direct comparison, females after mild TBI exhibited behavioral changes and reduced hippocampal neurogenesis, whereas cognitive changes in males were less pronounced [214]. Age may also modify the consequences of TBI: older animals may develop more pronounced motor and cognitive impairments compared with young animals [213], and differences are also detected at the level of synaptic and intracellular mechanisms, including processes associated with cAMP [51].

Differences between species and strains may also affect injury outcomes. The same TBI model can cause different anatomical and functional consequences in rats and mice, particularly when there are differences in injury parameters, craniotomy, anesthesia, and assessment time points. In CCI, a relatively localized cortical injury is usually formed, whereas FPI may cause more widespread pathology, the characteristics of which depend on the location of impact. Midline FPI is usually associated with more diffuse injury, whereas parasagittal or lateral FPI is associated with more pronounced focal injury of the ipsilateral cortex and distant effects. Direct comparisons of CCI and FPI revealed differences in behavioral and histological outcomes, and the severity of CCI correlated with neurological and motor impairments [215]. In addition, the location of the craniotomy itself may affect behavioral measures.

The type of injury may also affect the magnitude of the subsequent Sham effect. In CCI, Sham effects were predominantly limited to morphological and cellular measures: the number of dead neurons increased and the amygdala volume decreased compared with naïve animals, whereas most structural changes were significantly more pronounced after CCI [6,37]. In fluid percussion models, Sham effects also affected functional measures. In FPI, they were detected in electrophysiological parameters, including population spike amplitude in CA1, fEPSP slope, and long-term potentiation [48]. At low FPI pressure, craniotomy itself reproduced a number of TBI features, including neuroinflammation, neuronal and structural changes, and reduced grip strength, whereas the additional effect of percussion was small. At low FPI pressure, perioperative factors themselves reproduced a number of TBI features, including neuroinflammation, neuronal and structural changes, and reduced grip strength, whereas the additional effect of percussion was small.

In LFPI, Sham-associated changes in metabolic and molecular measures were also observed, depending on the observation time and the parameter under study [141]. Thus, Sham effects differ between TBI models and may make a particularly substantial contribution to the phenotype of some variants of fluid percussion. However, the absence of standardized effect sizes in most studies does not allow this to be considered formal evidence of differences between models, but only indicates model-dependent heterogeneity of Sham effects. Thus, direct comparisons should be interpreted with caution, giving preference to experimental designs with animals matched for sex, age, and characteristics.

During TBI modeling, the administration of anesthesia is an important parameter that must be strictly adhered to. In TBI research, it is extremely important to use a standardized anesthesia protocol based on the experimental goals of each specific study to control for the confounding effects of anesthesia on outcome parameters. When using animal models to reproduce clinical TBI, anesthetics are usually administered during surgical procedures to minimize pain and discomfort and to ensure immobilization [8].

However, some experimenters prefer to include an additional group of animals that receive anesthesia only, in order to determine the influence of this factor on the results. At present, the issue related to anesthesia remains insufficiently studied. There are numerous inconsistencies and contradictions concerning this topic. Articles devoted to this issue were analyzed; however, most of them indicated no significant differences between the anesthesia and control groups [80,201,202]. Therefore, for our study, it was decided not to include an additional anesthesia-only group in the analysis, due to the lack of reliable evidence confirming the influence of this procedure on outcome parameters, and previous research indicating minimal impact of anesthesia on experimental results. Given the insufficient data regarding the impact of anesthesia during the TBI modeling phase, an “anesthesia-only” group was not included in this review. Its inclusion would have necessitated a fourth experimental group—which is effectively absent from the analyzed literature—thereby rendering a comparison methodologically impossible. Nevertheless, this aspect is analyzed and discussed in detail in a separate chapter.

## 9. Conclusions

These findings indicate that sham surgery should not be considered a biologically neutral control condition. However, the alterations observed in sham-operated animals are generally less pronounced and more localized and are often more transient than those associated with TBI, although their magnitude and direction depend on the biological endpoint, anatomical region, observation period, injury model, and procedural conditions. Importantly, naïve, sham-operated, and TBI animals should not be regarded simply as points along a uniform continuum of increasing injury severity. Rather, they represent distinct experimental states, and some outcomes may be selectively affected by either sham surgery or traumatic impact. Differences between naïve and sham-operated animals should therefore be interpreted as sham procedure-associated rather than attributed exclusively to craniotomy itself, because anesthesia, analgesia, surgical technique, and other perioperative factors may contribute to the observed phenotype. Inclusion of both naïve and sham controls can provide a more informative framework for distinguishing injury-specific effects from sham procedure-associated changes when this distinction is central to the experimental question. In the context of enhancing the methodological rigor of qualitative research, it is advisable to reconsider the practice of excluding a group of intact (“naive”) animals. Despite the increased financial costs, such a control is essential for distinguishing the effects of the experimental intervention from background variations and artifacts. Accordingly, the costs associated with a naive control group should be viewed as an investment in the validity and reproducibility of the results. Such a three-group design should not be regarded as universally required, but may be scientifically and ethically justified when it is necessary for reliable interpretation of experimental outcomes. Within the 3Rs framework, an adequately informative design may help reduce the risk of ambiguous findings and unnecessary replication, while recognition of sham surgery as a biologically active and potentially burdensome intervention emphasizes the importance of Refinement through optimization of anesthesia, analgesia, surgical procedures, and postoperative care.

## Data Availability

No new data were created or analyzed in this study. Data sharing is not applicable to this article.

## Supplementary Materials

The following supporting information can be downloaded at https://www.mdpi.com/article/10.3390/ijms27209186/s1.

## Abbreviations

TBI	Traumatic brain injury
CCI	Controlled cortical impact
FPI	Fluid percussion injury
WDI	Weight drop-impact acceleration injury
I/A	Impact-acceleration model
CHIMERA	Closed-head impact model of engineered rotational acceleration
PTSD	Post-traumatic stress disorder
DAI	Diffuse axonal injury
BBB	Blood–brain barrier
LTP	Long-term potentiation
MWM	Morris water maze
RWM	Radial water maze
BM	Barnes maze
NORT	Novel object recognition test
AST	Attentional set-shifting test
GAD	Generalized anxiety disorder
OF	Open field
EPM	Elevated plus maze
MCSF	Multivariate concentric square field
NBS	Neurobehavioral scores
NSS	Neurological severity scores
BDNF	Brain-derived neurotrophic factor
GFAP	Glial fibrillary acidic protein
S100B	S100 calcium-binding protein B
MBP	Myelin basic protein
APP	Amyloid precursor protein
UCHL1	Ubiquitin C-terminal hydrolase L1
MAP2	Microtubule-associated protein 2
IBA1	Ionized calcium-binding adapter molecule 1
CatB	Cathepsin B
DynA	Dynorphin A
HA	Hyaluronic acid
mtDNA	Mitochondrial DNA
TSPO	Translocator protein
IL	Interleukin
TNF-α	Tumor necrosis factor A
NFκB	Nuclear factor kappa B
MGB	Microbiota–gut–brain
