# Supplementary material for: Disentangling the Effects of Craniotomy and Traumatic Brain Injury in Experimental Models: A Narrative Review
Source: Int J Mol Sci. 2026 Oct 16;27(20):9186. doi: 10.3390/ijms27209186 (PMC13705088; doi:10.3390/ijms27209186)
Supplement: Supplementary file 1 [file ijms-27-09186-s001.zip › Table S1.pdf]

**Table S1.** Effect of craniotomy and TBI on body weight, morphological and neuronal changes				
**Indicator**	**Sham vs Naïve**	**TBI vs Naïve**	**TBI vs Sham**	**Reference**
Body weight	-	↓***	↓***	[32]
Body weight (12 month)	↓*	↓*	-	[34]
Body weight	-	↓*	↓*	[35]
Weight gain	-	↓*	-	[36]
Weight gain	-	↓*	↓*	[14]
Volume of the ipsilateral hemisphere	-	↓***	↓***	[14]
Volume of the dorsal cortex	-	↓***	↓**	[37]
Volume of the cortical tissue	-	-	-	[38]
Thickness of cortex	-	↓*	-	[38]
Volume of the corpus callosum above the lateral amygdala capsule	-	↓**	-	[37]
Volume of the hippocampus	-	-	-	[37]
Volume of the amygdala	↓**	↓**	-	[37]
Volume of the ventricular	-	-	-	[35]
Percentage of lateral ventricle volume (contralateral hemisphere)	-	↑***	↑***	[14]
Brain water content	-	↑*	-	[35]
Number of dead neurons	↑**	↑***	↑*	[6]
Number of dark neurons	-	↑**	↑**	[6]
Proportion of microglial cells on day 7 after TBI	-	↑*	↑*	[45]
Proportion of activated astrocytes on day 7 after TBI 1-H	-	↑*	↑*	[45]

Table S1. Effect of craniotomy and TBI on body weight, morphological and neuronal changes. ↑ - increase in the parameter value in the group, ↓ - decrease in the parameter value in the group, "- "- absence of significant differences between the groups. * - standard level of statistical significance ($p \leq 0.05$), ** - high level of statistical significance ($p \leq 0.01$), *** - high level of statistical significance ($p \leq 0.001$). The presence of arrows without indication of the significance level was noted in cases of absence of statistically significant differences between the groups but corresponded to the results reported by the author in the article for the given parameters.
