# Supplementary material for: Disentangling the Effects of Craniotomy and Traumatic Brain Injury in Experimental Models: A Narrative Review
Source: Int J Mol Sci. 2026 Oct 16;27(20):9186. doi: 10.3390/ijms27209186 (PMC13705088; doi:10.3390/ijms27209186)
Supplement: Supplementary file 1 [file ijms-27-09186-s001.zip › Table S10.pdf]

**Table S10.** Effect of craniotomy and TBI on mitochondrial dysfunctions					
**Indicator**	**Method**	**Sham vs Naïve**	**TBI vs Naïve**	**TBI vs Sham**	**Reference**
mtDNA/nDNA	qPCR	-	↓*	-	[5]
Amount of mtDNA damage	Real-time long-range PCR	↑*	↑**	-	[5]
Amount of mtDNA damage in the ipsilateral region of the cortex	Long-fragment PCR	-	-	-	[78]
Amount of mtDNA damage in the hippocampus		-	-	-	[78]
Normalized mtDNA copy number in the cortex		-	↓***	↓**	[78]
Normalized mtDNA copy number in the hippocampus		-	↓*	-	[78]
Level of cell-free mtDNA in blood plasma		-	-	-	[78]
mtDNA level in the plasma	qPCR		↑*	↑*	[79]
TSPO expression levels	Immunohistochemistry	-	↑	↑	[14]
TSPO mRNA	qRT-PCR	-	↑*	↑*	[14]
Tfam Normalized expression in the mouse cerebral cortex	RT-PCR	↑*	-	↓*	[78]
Tfam Normalized expression in the mouse hippocampus		-	↓*	-	[78]
Pink1 Normalized expression in the mouse cerebral cortex		-	-	↑*	[78]
Pink1 Normalized expression in the mouse hippocampus		-	-	-	[78]
Prkn Normalized expression in the mouse cerebral cortex	RT-PCR	-	-	-	[78]
Prkn Normalized expression in the mouse hippocampus		-	-	-	[78]
Ppargc1a Normalized expression in the mouse cerebral cortex	RT-PCR	-	-	-	[78]
Ppargc1a Normalized expression in the mouse hippocampus		-	-	-	[78]
Nrf1 Normalized expression in the mouse cerebral cortex	RT-PCR	-	-	-	[78]
Nrf1 Normalized expression in the mouse hippocampus		-	-	-	[78]
Nfe2L2 Normalized expression in the mouse cerebral cortex	RT-PCR	-	-	-	[78]

Nfe2L2 Normalized expression in the mouse hippocampus		-	-	-	[78]

Table S10. Effect of craniotomy and TBI on mitochondrial dysfunctions. ↑ - increase in the parameter value in the group, ↓ - decrease in the parameter value in the group, "- "- absence of significant differences between the groups. * - standard level of statistical significance ($p \leq 0.05$), ** - high level of statistical significance ($p \leq 0.01$), *** - high level of statistical significance ($p \leq 0.001$). The presence of arrows without indication of the significance level was noted in cases of absence of statistically significant differences between the groups but corresponded to the results reported by the author in the article for the given parameters. qPCR - quantitative polymerase chain reaction, TSPO - translocator protein, Tfam - transcription factor A, mitochondrial, Pink1 - PTEN induced kinase 1, Prkn - parkin RBR E3 ubiquitin protein ligase, Nrf1 - nuclear respiratory factor 1, Nfe2l2 - Nuclear Factor Erythroid 2-Like 2
