# Supplementary material for: Disentangling the Effects of Craniotomy and Traumatic Brain Injury in Experimental Models: A Narrative Review
Source: Int J Mol Sci. 2026 Oct 16;27(20):9186. doi: 10.3390/ijms27209186 (PMC13705088; doi:10.3390/ijms27209186)
Supplement: Supplementary file 1 [file ijms-27-09186-s001.zip › Table S11.pdf]

**Table S11.** Effect of craniotomy and TBI on alterations in blood microRNA					
**miRNA**	**Method**	**Sham vs Naïve**	**TBI vs Naïve**	**TBI vs Sham**	**Reference**
miR-9a-3p	Quantitative RT-PCR	↑*	↑**	↑**	[172]
		↑*	↑***	↑***	[173]
miR-136-3p		↑*	↑**	↑**	[172]
		↑**	↑***	↑***	[173]
miR-434-3p		↑**	↑**	↑**	[172]
		↑***	↑***	↑***	[173]
miR-323-3p		↑***	↑***	↑***	[173]
miR-124-3p			↑***	↑***	[173]
miR-212-3p		↑*	↑***	↑***	[173]
miR-132-3p		↑*	↑***	↑***	[173]
miR-28-3p		-	-	-	[173]

Table S11. Effect of craniotomy and TBI on alterations in blood microRNA. ↑ - increase in the parameter value in the group, ↓ - decrease in the parameter value in the group, "- "- absence of significant differences between the groups. * - standard level of statistical significance ($p \leq 0.05$), ** - high level of statistical significance ($p \leq 0.01$), *** - high level of statistical significance ($p \leq 0.001$). The presence of arrows without indication of the significance level was noted in cases of absence of statistically significant differences between the groups but corresponded to the results reported by the author in the article for the given parameters. RT-PCR - real-time polymerase chain reaction, miR – microRNA.
