# Supplementary material for: Disentangling the Effects of Craniotomy and Traumatic Brain Injury in Experimental Models: A Narrative Review
Source: Int J Mol Sci. 2026 Oct 16;27(20):9186. doi: 10.3390/ijms27209186 (PMC13705088; doi:10.3390/ijms27209186)
Supplement: Supplementary file 1 [file ijms-27-09186-s001.zip › Table S12.pdf]

Table S12. Effect of craniotomy and TBI in inflammatory processes						
**Marker**	**Method**	**Indicator**	**Sham vs Naïve**	**TBI vs Naïve**	**TBI vs Sham**	**Reference**
IL1α	ELISA	protein levels	-	-	-	[5]
IL-1β	Quantitative RT-PCR	mRNA level	-	-	-	[5]
		mRNA level after 6 h.	-	↑**	↑**	[7]
		mRNA level after 1day	-	↑*	↑*	[7]
		mRNA level after 3 days	-	↑*	↑*	[7]
		mRNA level after 7 days	-	↑*	↑*	[7]
		mRNA level after 21 days	-	↑*	↑*	[7]
	ELISA	protein levels, day 7	-	↑*	↑*	[7]
		protein levels, day 21	-	-	-	[7]
	Western blot analysis	Levels in cerebral cortex	↑*	↑***	-	[158]
		Levels in thalamus	↑***	↑***	-	[158]
IL-4	Quantitative RT-PCR	mRNA level after 6 h.	-	-	-	[7]
		mRNA level after 1day	-	-	-	[7]
		mRNA level after 3 days	-	-	-	[7]
		mRNA level after 7 days	-	-	↑*	[7]
		mRNA level after 21 days	-	-	-	[7]
IL-6	Quantitative RT-PCR	mRNA level	-	-	-	[5]
		mRNA level after 6 h.	-	↑**	↑**	[7]
		mRNA level after 1 day	-	↑*	↑*	[7]
		mRNA level after 3 days	-	↑*	↑*	[7]
		mRNA level after 7 days	-	↑*	↑*	[7]
		mRNA level after 21 days	-	↑*	↑*	[7]
	Western blot analysis	Levels in cerebral cortex	↑***	↑***	↑***	[158]
		Levels in thalamus	-	↑***	↑***	[158]
CLCF1	RT-PCR	Relative expression level in the ipsilateral cortex	-	↑*	↑*	[137]
		Relative expression level in the ipsilateral thalamus	-	↑***	↑**	[137]
IL-10	Quantitative RT-PCR	mRNA level after 6 h	-	↑**	-	[7]
		mRNA level after 1day	-	↑*	-	[7]
		mRNA level after 3 days	-	↑**	-	[7]
		mRNA level after 7 days	-	↑**	↑**	[7]
		mRNA level after 21 days	-	↑**	↑**	[7]
	ELISA	protein levels, day 7	-	↓*	↓*	[7]
		protein levels, day 21	-	-	-	[7]
TNF-α	Quantitative RT-PCR	mRNA level	-	-	-	[5]
		mRNA level after 6 h	↑*	↑**	↑**	[7]
		mRNA level after 1day	-	↑*	-	[7]
		mRNA level after 3 days	-	↑**	-	[7]
		mRNA level after 7 days	-	↑**	↑**	[7]
		mRNA level after 21 days	-	↑**	↑**	[7]
	ELISA	protein level , day 7	-	↑*	↑*	[7]
		protein levels, day 21	-	-	-	[7]
	Western blot analysis	Levels in cerebral cortex	↑***	↑***	↑***	[158]
		Levels in thalamus	↑*	↑***	↑*	[158]
NFkβ	ELISA	protein levels	↑**	↑*	-	[5]

	RT-PCR	Normalized expression in the mouse cerebral cortex	-	-	↑*	[78]
		Normalized expression in the mouse hippocampus	-	-	-	[78]
Ptgs2	Quantitative RT-PCR	mRNA level	-	↑*	-	[5]
MPO	Spectrophotometrically	MPO activity	-	↑*	↑*	[188]

Table S12. Effect of craniotomy and TBI in inflammatory processes. ↑ - increase in the parameter value in the group, ↓ - decrease in the parameter value in the group, "- "- absence of significant differences between the groups. * - standard level of statistical significance ($p \leq 0.05$), ** - high level of statistical significance ($p \leq 0.01$), *** - high level of statistical significance ($p \leq 0.001$). The presence of arrows without indication of the significance level was noted in cases of absence of statistically significant differences between the groups but corresponded to the results reported by the author in the article for the given parameters. CLCF1 - cardiotrophin-like cytokine factor 1, RT-PCR - real-time polymerase chain reaction, ELISA - Enzyme-Linked Immunosorbent Assay, TNF-α - tumor necrosis factor, NFkβ - nuclear factor kappa B, Ptgs2 - prostaglandin-endoperoxide synthase 2, MPO – myeloperoxidase.
