# Supplementary material for: Disentangling the Effects of Craniotomy and Traumatic Brain Injury in Experimental Models: A Narrative Review
Source: Int J Mol Sci. 2026 Oct 16;27(20):9186. doi: 10.3390/ijms27209186 (PMC13705088; doi:10.3390/ijms27209186)
Supplement: Supplementary file 1 [file ijms-27-09186-s001.zip › Table S13.pdf]

Table S13. Comparative analysis of microenvironmental factors and their relationship with the microbiome			
**Diet composition**	**Housing**	**Microclimate**	**Reference**
Unclear	The mice were kept under standard conditions: temperature = 25 °C, a 12 h light cycle, and a relative humidity of at least 40%.		[5]
• Control diet: contained cellulose as a source of fiber (non-fermentable). Manufacturer: TestDiet, catalog number 1817794-203.<br>• Diet with inulin: contained 8% inulin (fermentable fiber). Manufacturer: TestDiet, catalog number 1817795-203.<br>• Feeding regimen: mice had free access to food (ad libitum). The weight of the mice and the remaining feed was measured every two weeks to assess consumption.	The mice were kept individually throughout the entire study. This was done to prevent the exchange of feces, as mice are coprophagic (they eat their own excrement).	The mice were housed in an AAALAC-accredited PHS facility under specific pathogen-free conditions.	[193]
