# Supplementary material for: Disentangling the Effects of Craniotomy and Traumatic Brain Injury in Experimental Models: A Narrative Review
Source: Int J Mol Sci. 2026 Oct 16;27(20):9186. doi: 10.3390/ijms27209186 (PMC13705088; doi:10.3390/ijms27209186)
Supplement: Supplementary file 1 [file ijms-27-09186-s001.zip › Table S14.pdf]

Table S14. Types of TBI and craniotomy simulations in the reviewed articles

Animals used	Weight, age, sex and number of animals	Type of injury	Type of anesthesia	Craniotomy/TBI device	Craniotomy opening diameter	Duration of the postoperative period	Reference
Sprague-Dawley rats	Seventy-three female rats weighed an average 251 g (age 15 weeks)	CCI	5% isoflurane in 22% O2/78% N2; bupivacaine (Marcaine®) 0.25%] buprenorphine (Temgesic®) and carprofen	Surgical drill with a diamond tip of 0.5 mm diameter (Microspeed 317 IN; Silfradent, Forli, Italy)	-	The rats were sacrificed at 1, 3, 7, 14, and 28 days post-injury.	[144]
C57BL6 mice	Thirty-six male mice weighing 26±4 g	CCI	Isoflurane; xylocaine	A CCI device was used (Israelsson et al., 2006), with an impact depth of 0.5 mm and a piston velocity of 3.3 m/sec.	4-mm-diameter	-	[95]
Sprague-Dawley rats	Forty-eight male rats weighing 170–200 g	CCI	Isoflurane	The rats were mounted in the injury device, secured by ear bars and incisor bar and spontaneously anesthetized with a 1–2% isoflurane in O2 via blow-by nose cone connected to a charcoal canister passive isoflurane scavenger.	10-mm craniotomy	Four animals per group were sacrificed at 4 h, 1, 3, and 7 days post-CCI.	[161]
Sprague-Dawley rats	The experiment used 60 male rats	CCI	Isoflurane	Injury was produced using a 3-mm metal impactor tip with a depth of penetration of 1.5 mm, at velocity of 5.5 ± 0.3 m/sec, and a duration of deformation of 50 msec.	3 mm craniotomy	~ 130 days post operation	[80]
C57BL/6 J mice	Mice weighing 28-30 g	CCI	Isoflurane	Impact was delivered by a 3 mm diameter impactor using a pneumatically controlled device (TBI 0310 Impactor, Precision System Instruments).	4 mm craniotomy	Animals were sacrificed after ~ 3 months post-injury.	[37]
C57Bl/6 J mice	Brain injury was performed at P60, and experiments were performed 0.5 or 3 months after TBI	CCI	Isoflurane	A computer-controlled pneumatically driven impactor (TBI-0310, Precision Systems and Instrumentation).	A ~4-5 mm craniotomy	-	[38]
C57BL/6 mice	Male mice, aged 2 months. The experiment used 48 mice	CCI	Isoflurane	Dental burr (JessNail DM206, Hong Kong, China); stereotaxic impactor, the 68099II Precise Impactor (RWD, Nanshan, China)	4 mm diameter hole	Following a 72 h period post operation	[5]

C57B/6 mice	9-10-wk-old male mice. The experiment used 145 mice	CCI AND CHI	Isoflurane	CHI: was completed with a centered 400 g	4 mm craniotomy	Mice were survived to 14 and 30 days	[23]
CD-1 mice	Adult male (~4 months old) mice. The experiment used 62 mice	CCI	Isoflurane	4–5 mm craniotomy was performed in a stereotaxic frame and moderate-to-severe CCI was performed using a Precision Cortical Impactor (Hatteras Instruments, NC).	4-5 mm craniotomy	The animals were sacrificed a month.	[115]
CD-1 mice	Outbred male mice aged of seven weeks	CCI	Isoflurane	Using a micro drill (Dremel® Micro 8050–35, Bosch Power Tools B.V., Breda, The Netherlands) with a round-head bur, a craniotomy was performed over the left fronto-parietal cortex without damage to the underlying dura. CCI was triggered using the control box of the electromagnetic CCI device.	4 mm diameter	-	[100]
Pregnant New Zealand white rabbits	New Zealand white rabbits (n = 166; 48 male and 118 female) were from 28 independent litters	CCI	Dexmedetomidine hydrochloride; isoflurane.	A controlled cortical impact (CCI) device was used to injure the exposed cortex.	8-mm craniotomy	Animals were sacrificed at 6 h and 1, 3, 7, and 21 days post-injury	[7]
Pregnant New Zealand rabbits	New Zealand rabbits (n=98; 31 male and 67 female) were from 19 independent litters	CCI	Dexmedetomidine hydrochloride; isoflurane.	A controlled cortical impact (CCI) device was used to injure the exposed cortex.	8-mm craniotomy	Animals were sacrificed at 1, 3, 7, and 21 days post-injury	[14]
Sprague—Dawley rats	Male rats were obtained on post-natal day (P) 7–10. The experiment used 18-24 rats	CCI	Isoflurane	A craniotomy was performed over the left parietal cortex using a high-speed dental drill. CCI was then delivered (Pittsburgh Precision Instruments, Pittsburgh, PA) to the left parietal cortex using a 5-mm rounded tip to deliver a 2-mm deformation at 4 meters/second velocity and 100 millisecond duration.	6-mm×6-mm craniotomy	Animals were sacrificed at 1, 2, 3, 7 and 14 days post-injury	[136]
Sprague-Dawley rats	On PND 16 - 18 male rats. The experiment used 60 rats	CCI	Isoflurane	The rat was placed into a stereotaxic frame (David Kopf, Tujunga, CA) and a craniotomy was performed over the left parietal cortex using a	6 × 6-mm	The animals were sacrificed 62–69 days after the operation.	[36]

				high-speed dental drill. TBI was then performed by controlled cortical impact (Pittsburgh Precision Instruments, Pittsburgh, PA).			
CF-1 mice	Adult male mice weighing 29–31 g. The experiment used 103 mice	CCI	Tribromoethanol	Using sterile procedures, the skin was retracted, and a 4-mm craniotomy was made lateral to the sagittal suture centered between the bregma and lambda. The exposed somatosensory cortex was injured using a pneumatically-controlled impactor device (TBI 031; Precision Systems & Instrumentation, Fairfax Station, VA).	4-mm craniotomy	Animals were sacrificed at 24 h and 7 days after the operation.	[160]
C57BL/6 mice	Male mice (approximately 8 weeks of age and weighing approximately 22–24 g). The experiment used 84 mice	CCI	Isoflurane	The head was fixed in a stereotaxic frame, and a high-speed drill was used to create a right-sided 4-mm craniotomy positioned at Bregma exposing the somatosensory cortex. TBI was induced using an electromagnetic stereotaxic impactor (Leica Biosystems Richmond formerly MyNeuroLab, Richmond, IL, USA) consisting of a 3-mm diameter, rounded aluminum tip.	4-mm craniotomy	Animals were sacrificed after 7, 14 and 28 days after the operation.	[142]
Sprague-Dawley rats	Adult male rats weighing 300–325 g. The experiment used 47 rats	CCI	Isoflurane	A craniectomy was performed in the right hemisphere with a dental drill. A TBI was then produced by impacting the exposed right parietal cortex to a depth of 2.6, 2.8, and 3.0 mm tissue deformation at 4 m/sec.	-	-	[87]
Sprague–Dawley rats	Male rats weighing 250–300 g. The experiment used 44 rats	CCI	Sodium pentobarbital	A right parietal bone window was made under aseptic conditions with dental drill. Trauma was performed by dropping a steel rod weighing 40 g onto a steel gasket resting on the dura from 25 cm height.	5 mm diameter	Animals were sacrificed at 6 h. and 1, 3, 5, 7 days post-injury	[169]
Wistar-Albino rats	Female rats, weighing 170 to 200 g. The experiment used 56 rats	CCI	Xylazine; ketamine hydrochloride	Right frontoparietal craniectomies were carried out lateral to the sagittal suture by dental drill system. Rats	-	Animals were sacrificed at 2, 8 and 24 h. post-injury	[68]

				were injured by a stainless-steel rod (5-mm diameter, weighing 140 g) weight dropped vertically through a calibrated tube from a height of 10 cm onto the exposed dura.			
C57BL/6 mice	Twelve-week old male mice	CCI	Isoflurane	Craniotomy was performed by a surgeon using a hand-held 5 mm trephine over the motor cortex. Mice were then subjected to CCI using a 3 mm flat-tip.	-	Animals were sacrificed after 1, 2, 3, 7, 10 and 21 days after the operation.	[41]
Sprague-Dawley rats	Male rats weighing 300–400 g. The experiment used 34 rats	CCI	Isoflurane	A pneumatic piston impacted the cortical tissue to a depth of 2 mm, values consistent with a moderate TBI insult.	-	-	[166]
CD-1 mice	Adult male mice (~4 months old). The experiment used 70 mice	CCI	Isoflurane	Utilizing a Precision Cortical Impactor (Hatteras instruments, NC) with a 3-mm-diameter tip, a moderate-to-severe impact was delivered.	-	-	[111]
C57BL/6 mice	Two-month-old male mice. The experiment used 35 mice	CCI	Chloral hydrate	Using the 68099II Precise Impactor (RWD), a trauma in the right hemisphere was inflicted with a 3 mm diameter impactor tip.	diameter of 4 mm	Animals were sacrificed after 24 days post-injury	[79]
CD1 mice	Eight-week-old male mice (~25 g)	CCI	Isoflurane	The CCI injury was similarly induced in all mice using a 3 mm diameter rigid impactor driven by a pneumatic piston (Leica Biosystems, Buffalo Grove, IL, USA)	-	-	[61]
Sprague-Dawley rats	Age-matched female rats weighing 225–250 g. 28 rats in the experiment	FPI	-	Rats were anesthetized and connected to the fluid-percussion injury (FPI) device (Dragonfly Research & Development, Inc.) via the Leur-Loc syringe	-	-	[62]
Sprague-Dawley rats	Male rats weighing 300-350 g	FPI	Sodium pentobarbial anesthesia; isoflurane	The fluid-percussion injury device consisted of a Plexiglas cylinder filled with double distilled H,O. A Plexiglas piston was mounted at one end of the cylinder; at the opposite end, a male Luer-Lok fitting, containing a tube, was attached to a	-	-	[131]

				metal housing with an enclosed pressure transducer.			
Sprague-Dawley rats	Rats weighing 250-350 g	FPI	Isoflurane	Fluid percussion injury device at 2.1 atm	-	Animals were sacrificed at 3 h. post-injury	[157]
Rats	The experiment used 150 rats	FPI	-	-	-		[33]
Wistar rats	Adult male rats weighing 230-260 g. The experiment used 150 rats	FPI	Pentobarbital anesthesia; edaravone	Percussion device (HPD-1700: Dragonfly R & D, Inc., Ridgeley, WV, USA) consisted of a stainless steel cylinder with a 2.7 cm bore and a 7.6 cm piston stroke that was connected to the hub via high-pressure tubing.	-	Animals were sacrificed after 7 days post-injury	[57]
Sprague-Dawley rats	Adult male rats weighing 300-350 g	FPI	Isoflurane	A modified Luer-Lok syringe hub (Becton-Dickinson, Franklin Lakes, NJ) was placed over the exposed and intact dura, bonded in place with cyanoacrylic adhesive, and covered with dental acrylic.	A 4.0 mm diameter	-	[34]
Wistar rats	Used adult male rats (2.5–3 months of age at the time of the intervention) (250–350 g). The experiment used about 15 rats	FPI	Equithesin, a mixture of sodium pentobarbital, chloral hydrate, magnesium sulfate, propylene glycol, and absolute ethanol	A fluid percussion apparatus developed in our laboratory produced the FPI.	3 mm craniotomy	-	[105]
Sprague–Dawley rats	Male rats (24–31 days post-natal)	FPI	Halothane	Saline-filled FP injury device via a Leure-Lock	4-mm midline burr hole	-	[48]
Sprague–Dawley rats	Adult male rats, weighing 350–400 g	FPI	Isoflurane	A modified 20-gauge Luer Lok syringe hub (Becton-Dickinson, Franklin Lakes, NJ) was placed over the exposed dura, bonded in place with cyanoacrylic adhesive, and covered with dental acrylic. Rats were connected to the fluid percussion trauma device (Custom Design and Fabrication, Virginia Commonwealth University, VA.	4.0-mm-diameter	Animals were sacrificed at 24 h., 2 weeks, and 3, 6, 12 months post-injury	[81]

C57Bl/6 mice	Male mice weighing 25 ± 2 g	FPI	Ketamine; xylazine	Craniectomy was performed using a trephine of 2 mm × 2.5 mm inner and outer diameters respectively. Fluid Percussion Device Model FP 302 (Am Scien Instruments, 4408 Hungary Glen Terrace, Richmond, VA 23294 U.S.A.) was used to cause mTBI.	2.5 mm diameter	Animals were sacrificed after 7 days post-injury	[6]
C57BL/6J mice	Adult male mice (5–7 weeks, 20–25 g)	FPI	Sodium pentobarbital	The hub was filled with isotonic sterile saline and a 32 cm piece of high-pressure tubing from the FPI device (Department of Biomedical Engineering, Virginia Commonwealth University, Richmond, VA, USA) was attached to the Luer-loc fitting of the hub.	-	-	[49]
Thy1-YFP-H mice	mTBI was induced at 7–8 weeks of age	FPI	Isoflurane	Fluid percussion injury by releasing a pendulum onto a fluid-filled piston to induce a brief fluid pressure pulse upon the intact dura, as previously described	-	Animals were sacrificed after 1 and 2 days post-injury	[56]
C57BL/6J mice	Experiments were performed on 5- to 7-week old, 20–25 g mice	FPI	-	On the second day, a 20-ms pulse (ranging from 1.4 to 2.1 atm) of saline was delivered to the brain resulting in a mild to moderate injury.	-	Animals were sacrificed after 1 and 7 days post-injury	[50]
Sprague–Dawley rats	Adult male rats (body weight 354–392 g at the time of injury). The experiment used 33 rats	FPI	Isoflurane	A circular craniectomy was performed with a trephine. They were connected to the fluid-percussion device equipped with a straight tip (AmScien Instruments, Richmond, VA, USA) through the male Luer–lock fitting, and the brain injury was induced.	diameter 5 mm	Animals were sacrificed after 1 and 2 days post-injury	[172]
Sprague-Dawley rats	Male rats (350–400 g). The experiment used 90 rats	mFPI	Isoflurane; oxide/oxygen	A 4-mm circular craniotomy was performed over the midline 2 mm behind the bregma suture. The animal was moved to the FPI-device, the piston was released, and TBI was induced.	4-mm circular craniotomy	Animals were sacrificed at 48 h. and 15 days post-injury	[35]

Wistar rats	Male rats weighing between 175 and 200 grams. Average body weight prior to surgery was 486 ± 42 grams. The experiment used 43 rats	LFPI	-	Animals underwent craniotomy prior to TBI by LFP (Fluid Percussion Injury (FPI), Model 01-B. Custom Design and Fabrication, Virginia Commonwealth University)	-	Animals were sacrificed after 19 days post-injury	[97]
Sprague–Dawley rats	Male rats weighing (250–350 g). The experiment used 40 rats	LFPI	Halothane	A controlled cortical impact device was used to induce TBI, as described previously. Injury was induced by impacting the right cortex (ipsilateral cortex) with a 6-mm-diameter tip at a rate of 6 m/s and a 2-mm compression.	-	Animals were sacrificed at 24 h. post-injury	[165]
Sprague Dawley rats	Adult male rats were received between 9 and 10 weeks of age. The experiment used 44 rats	LFPI	Buprenorphine; isoflurane	LFPI and shams underwent with manual trephination. LFPI rats were connected to the fluid percussion injury (FPI) device (AmScien Fluid Percussion Device model FP 302) through the Luer lock and were subjected to a ~3 atm pressure pulse.	5 mm left parietal craniotomy	-	[141]
Sprague Dawley rats	Eleven-week-old male mice	LFPI	Isoflurane; pentobarbital	Rats underwent left parietal craniotomy using trephine (Einstein) or electric drill (Melbourne). After regaining pain response, LFPI rats were connected to the straight connector of the FPI device (AmScien model FP 302, Richmond VA, USA) through the craniotomy hub, and received a pressure pulse	-	Animals were sacrificed after 2 days, 1, 2, 4 and 8 weeks after the operation	[152]
C57BL/6J mice	Sixty-seven male mice weighing 24–26 g. The experiment used 50 male rats	LFPI	Isoflurane	After exposing the skull, a trephine (3 mm outer diameter) was used to remove a bone disc. LFP device (Dragonfly R & D Inc., Ridgeley, WV).	3 mm craniotomy	Animals were sacrificed after 20-25 days post-injury	[98]
C57/Bl6 mice	Eight-week-old male mice were used	LFPI	Chloral hydrate; paraformaldehyde	-	-	Animals were sacrificed at 30 min., 1, 3. 6, 10, 18, 24-48 h., 5-7 days, 2 weeks and 1, 3 months post-injury	[43]

Sprague Dawley rats	Adult male rats weight 356 ± 13 g. The experiment used 257 rats	LFPI	Isoflurane	TBI was induced by LFPI. The impact pressure was adjusted to produce severe TBI with an expected acute post-impact mortality of 20–30% within the first 48 h.	-	Animals were sacrificed after ~ 6 months post-injury	[173]
Sprague–Dawley rats	36 male rats weighing approximately 300 grams	WDI	Hypnorm (fluanisonum, 10 mg=ml, fentanyl citrate, 0.315 mg=ml; XylocainAdrenalin (lidocaine hydrochloride, 5 mg=ml and adrenaline, 5 mg=ml)	A standardized parietal contusion was made by letting a 24 gram weight fall onto a rod with a flat end diameter of 1.8 mm from a height of 7 cm	2 mm craniotomy	-	[13]
Wistar rats	Male rats (older than 2 months, weighing 350-450 g). The experiment used 41 rats	WDI	Halothane	Percussive injury to the right parietal cortex was induced by dropping a 10 g brass rod (3 mm in diameter) from a height of 5 cm through a glass guide tube onto the exposed surface of the brain.	-	Animals were sacrificed at 24 h. post-injury	[188]
Wistar rats	Male rats weighing 160-220 g	WDI	Chloral hydrate solution	To simulate a traumatic injury to the left hemisphere of the brain, a movable Teflon piston was placed in a trepanation hole drilled in the skull above the sensorimotor cortex and then was struck from a height of 10 cm with a free-fall load weighing 50g.	-	Animals were sacrificed after 7 days post-injury	[45]
C57BL/6J mice	Adult male mice. The experiment used 60 mice	WDI	Isoflurane	The weight-drop TBI was inflicted by dropping a weight onto a blunt surface, which accelerates head movement with minimal local impact at the point of application of the traumatic force.	-	-	[85]
C57BL/6 wild-type mice	Male mice at 4 months of age. The experiment used 71 mice	CHI	Isoflurane	An electromagnetic impactor was used to deliver a single controlled mid-line cortical impact at 5.0 ± 0.2 m/s, 100ms dwell time, and 1.0 ± 0.2 mm impact depth.	-	Animals were sacrificed after 3-4 (8 for several mice) months post-injury	[193]
Sprague-Dawley rats	Male rats weighing 250–300 g. The experiment used 18-30 rats	PBBI	Isoflurane	Induction of a 10% unilateral frontal PBBI was performed after conducting a craniotomy followed by stereotaxic insertion of a specially	4-mm-diameter	Animals were sacrificed after 1, 3 and 7 days post-injury	[159]

				designed probe into the right hemisphere of the brain (Mitre Corporation, McLean, VA).			
Canines (Dogs)	All female canines were 1-year-old with an average weight of 11 kg (range, 10-11.8 kg). The experiment used 12 canines	Ecci	Sodium pentobarbital	A miniature hand-held cranial drill (RWD life science company, Shenzhen, China) was used to create a bony window of 3.5 cm in the long axis and 3 cm	-	-	[59]
C57BL/6 mice	Male mice (3 months old, 26–28 g). The experiment used 95 mice	WDI	4.5% isoflurane in 100% oxygen	The model was generated using a specialized IMP-1020 impactor (Shanghai TOW Intelligent Technology, Shanghai, China)	4 mm-diameter	Animals were sacrificed after 7 days post-injury	[158]
C57BL/6 mice	Male mice (3 months old, 26–28 g). The experiment used 75 mice	WDI	4.5% isoflurane in 100% oxygen	A specialized IMP-1020 impactor (Shanghai TOW Intelligent Technology, Shanghai, China) was employed to deliver the injury.	4 mm-diameter	Animals were sacrificed after 7 days post-injury	[137]
C57BL/6 mice	67 male mice with an average body weight of 18 g were used, but 11 mice died during the postoperative period	CCI	Mixture of Zoletil 50	A craniotomy was then performed by drilling a 4 mm-diameter burr hole in the skull using a dental drill (JessNail DM206, China). TBI was induced using a stereotaxic impactor (Model 68099II Precise Impactor, RWD,China).	4 mm-diameter	Animals were sacrificed after 24 days post-injury	[78]
