# Supplementary material for: Disentangling the Effects of Craniotomy and Traumatic Brain Injury in Experimental Models: A Narrative Review
Source: Int J Mol Sci. 2026 Oct 16;27(20):9186. doi: 10.3390/ijms27209186 (PMC13705088; doi:10.3390/ijms27209186)
Supplement: Supplementary file 1 [file ijms-27-09186-s001.zip › Table S15.pdf]

**Table S15.** The risk of bias							
**Randomization**	**Temperature control**	**Standardization of anesthesia**	**The craniotomy method**	**Perioperative care**	**Uniformity of observation periods**	**Funding and conflict of interest**	**Reference**
Unclear	Low risk	Low risk	Low risk	Low risk	Low risk	Low risk	[144]
Low risk	Low risk	Low risk	Low risk	Low risk	Low risk	Low risk	[95]
Low risk	Low risk	Low risk	Low risk	Unclear	Low risk	Low risk	[161]
Low risk	Unclear	Low risk	Low risk	Unclear	Low risk	Low risk	[80]
Low risk	Low risk	Low risk	Low risk	Unclear	Low risk	Low risk	[37]
Low risk	Unclear	Low risk	Low risk	Unclear	Low risk	Low risk	[38]
Unclear	Low risk	Low risk	Low risk	Unclear	Low risk	Low risk	[5]
Unclear	Unclear	Low risk	Low risk	Unclear	Low risk	Low risk	[23]
Unclear	Unclear	Low risk	Low risk	Unclear	Low risk	Low risk	[115]
Low risk	Low risk	Low risk	Low risk	Low risk	Low risk	Low risk	[100]
Low risk	Low risk	Low risk	Low risk	Unclear	Low risk	Unclear	[7]
Low risk	Low risk	Low risk	Low risk	Unclear	Low risk	Low risk	[14]
Low risk	Low risk	Low risk	Low risk	Low risk	Low risk	Low risk	[136]
Low risk	Low risk	Low risk	Low risk	Low risk	Low risk	Low risk	[36]
Unclear	Low risk	Low risk	Low risk	Unclear	Low risk	Low risk	[160]
Low risk	Low risk	Low risk	Low risk	Low risk	Low risk	Unclear	[142]
Low risk	Low risk	Low risk	Low risk	Unclear	Low risk	Low risk	[87]
Low risk	Unclear	Low risk	Low risk	Unclear	Low risk	Low risk	[169]
Low risk	Unclear	Low risk	Low risk	Unclear	Low risk	Low risk	[68]
Unclear	Unclear	Low risk	Low risk	Unclear	Low risk	Low risk	[41]
Low risk	Low risk	Low risk	Low risk	Low risk	Low risk	Low risk	[166]
Unclear	Low risk	Low risk	Low risk	Low risk	Low risk	Low risk	[111]
Unclear	Low risk	Low risk	Low risk	Low risk	Low risk	Low risk	[79]
Low risk	Low risk	Low risk	Low risk	Unclear	Low risk	Low risk	[61]
Unclear	Unclear	Low risk	Unclear	Unclear	Low risk	Low risk	[62]
Unclear	Unclear	Low risk	Unclear	Unclear	Low risk	Low risk	[131]
Unclear	Unclear	Low risk	Unclear	Unclear	Low risk	Low risk	[157]
Low risk	Unclear	Unclear	Unclear	Unclear	Low risk	Low risk	[33]
Unclear	Low risk	Low risk	Low risk	Unclear	Low risk	Low risk	[57]
Unclear	Low risk	Low risk	Low risk	Low risk	Low risk	Low risk	[34]
Low risk	Unclear	Low risk	Low risk	Unclear	Low risk	Unclear	[105]
Low risk	Low risk	Low risk	Low risk	Low risk	Low risk	Low risk	[48]
Unclear	Unclear	Low risk	Low risk	Low risk	Low risk	Low risk	[81]
Unclear	Unclear	Low risk	Low risk	Unclear	Low risk	Low risk	[6]
Low risk	Low risk	Low risk	Low risk	Low risk	Low risk	Low risk	[49]

Unclear	Unclear	Low risk	Low risk	Unclear	Low risk	Low risk	[56]
Unclear	Unclear	Unclear	Low risk	Unclear	Low risk	Unclear	[50]
Unclear	Low risk	Low risk	Low risk	Low risk	Low risk	Low risk	[172]
Low risk	Low risk	Low risk	Low risk	Low risk	Low risk	Low risk	[35]
Low risk	Unclear	Unclear	Low risk	Unclear	Low risk	Low risk	[97]
Unclear	Low risk	Low risk	Low risk	Unclear	Low risk	Low risk	[165]
Low risk	Low risk	Low risk	Low risk	Low risk	Low risk	Low risk	[141]
Low risk	Unclear	Low risk	Low risk	Low risk	Low risk	Low risk	[152]
Low risk	Unclear	Low risk	Low risk	Low risk	Low risk	Low risk	[98]
Unclear	Unclear	Low risk	Low risk	Unclear	Low risk	Low risk	[43]
Low risk	Low risk	Low risk	Low risk	Unclear	Low risk	Low risk	[173]
Unclear	Unclear	Low risk	Low risk	Unclear	Low risk	Unclear	[13]
Unclear	Low risk	Low risk	Low risk	Low risk	Low risk	Unclear	[188]
Unclear	Unclear	Low risk	Low risk	Unclear	Low risk	Low risk	[45]
Unclear	Unclear	Low risk	Low risk	Unclear	Low risk	Low risk	[85]
Low risk	Low risk	Low risk	Low risk	Unclear	Low risk	Low risk	[193]
Unclear	Low risk	Low risk	Low risk	Unclear	Low risk	Low risk	[159]
Unclear	Low risk	Low risk	Low risk	Low risk	Low risk	Low risk	[59]
Unclear	Low risk	Low risk	Low risk	Low risk	Low risk	Low risk	[158]
Unclear	Low risk	Low risk	Low risk	Low risk	Low risk	Low risk	[137]
Unclear	Low risk	Low risk	Low risk	Unclear	Low risk	Low risk	[78]
