# Supplementary material for: Disentangling the Effects of Craniotomy and Traumatic Brain Injury in Experimental Models: A Narrative Review
Source: Int J Mol Sci. 2026 Oct 16;27(20):9186. doi: 10.3390/ijms27209186 (PMC13705088; doi:10.3390/ijms27209186)
Supplement: Supplementary file 1 [file ijms-27-09186-s001.zip › Table S2.pdf]

**Table S2.** Effect of craniotomy and TBI on electrophysiological parameters				
**Indicator**	**Sham vs Naïve**	**TBI vs Naïve**	**TBI vs Sham**	**Reference**
CA1 Population Spike Threshold	–	↑**	↑*	[48]
CA1 fEPSP Threshold	–	–	–	[48]
CA1 Population Spike Amplitude	–	↓***	↓**	[48]
CA1 fEPSP Slope	–	↓**	↓**	[48]
CA1 Long-Term Potentiation of fEPSP, HFS protocol	↓*	↓***	↓*	[48]
CA1 Long-Term Depression of fEPSP, LFS protocol	–	–	–	[48]
CA1 Long-Term Potentiation of fEPSP, HFS protocol	–	↓*	↓*	[48]
DG Long-Term Potentiation of Population Spike Amplitude	–	↓**	↓**	[57]
CA1 pyramids spontaneous miniature Inhibitory Postsynaptic Currents amplitude and frequency, mIPSCs	–	↓*	↓*	[49]
DG granule cells spontaneous miniature Inhibitory Postsynaptic Currents amplitude, mIPSCs	–	↑*	↑*	[49]
RMP	–	–	–	[56]
Input Resistance, R_in	–	–	–	[56]
Time Constant, τ	–	–	–	[56]
Action Potential Amplitude	–	↑*	↑*	[56]
Afterhyperpolarization Duration	–	↓*	↓*	[56]
Slope of the Frequency-Current Plot	–	↑*	↑*	[56]
Rheobase	–	↑*	↑*	[56]
Percentage of Intrinsically Bursting Neurons	–	↓*	↓*	[56]
MEP amplitude	–	↓**	↓**	[59]
MEP latency	–	↑**	↑**	[59]
Daily number of spikes and sharp waves at perilesional electrodes in ECoG	–	↑*	↑*	[61]
Daily number of spikes and sharp waves at contralateral anterior/posterior electrodes in ECoG	–	↑*	↑*	[61]
Estimated Embedding Dimension of ECoG signals	↓**	↓**	↓**	[61]

Table S2. Effect of craniotomy and TBI on electrophysiological parameters. ↑ - increase in the parameter value in the group, ↓ - decrease in the parameter value in the group, "- "- absence of significant differences between the groups. * - standard level of statistical significance ($p \leq 0.05$), ** - high level of statistical significance ($p \leq 0.01$), *** - high level of statistical significance ($p \leq 0.001$). The presence of arrows without indication of the significance level was noted in cases of absence of statistically significant differences between the groups but corresponded to the results reported by the author in the article for the given parameters. EPSP – excitatory postsynaptic potential, fEPSP – field excitatory postsynaptic potential, HFS - high frequency stimulation, LFS – low frequency stimulation, DG - dentate gyrus, mIPSC - miniature inhibitory postsynaptic currents, RMP - resting membrane potential, MEP - motor evoked potential, ECoG – electrocorticography.
