# Supplementary material for: Disentangling the Effects of Craniotomy and Traumatic Brain Injury in Experimental Models: A Narrative Review
Source: Int J Mol Sci. 2026 Oct 16;27(20):9186. doi: 10.3390/ijms27209186 (PMC13705088; doi:10.3390/ijms27209186)
Supplement: Supplementary file 1 [file ijms-27-09186-s001.zip › Table S3.pdf]

**Table S3.** Effect of craniotomy and TBI on peripheral targets				
**Indicator**	**Sham vs Naïve**	**TBI vs Naïve**	**TBI vs Sham**	**Reference**
Amplitude of baseline EUS EMG 1 Day	↑	↓	↓*	[62]
Amplitude of baseline EUS EMG 1 Week	↑*	↓	↓*	[62]
Amplitude of bursting EUS EMG 1 Day	↓	↓*	↓*	[62]
Amplitude of bursting EUS EMG 1 Week	↓*	↓*	-	[62]
Amplitude of bursting EUS EMG 1 Month	↓*	↓	↓*	[62]
Firing rate of baseline EUS EMG 1 Day	↓	↓*	↓*	[62]
Firing rate of baseline EUS EMG 1 Week	↑	↓	↓*	[62]
Firing rate of bursting EUS EMG 1 Day	↓	↓*	↓*	[62]
Firing rate of bursting EUS EMG 1 Week	↓*	↓*	-	[62]
Firing rate of bursting EUS EMG 2 Weeks	↓*	↓*	↑	[62]
Firing rate of bursting EUS EMG 1 Month	↓	↓*	↓	[62]
Peak voiding pressure 1 Day	↓*	↓*	↑	[62]
Peak voiding pressure 1 Week	↓	↓*	↓	[62]
Voiding frequency 1 Day	↑*	↑*	↑*	[62]
Voiding duration 1 Day	↓*	↓*	↓	[62]
Voiding duration 1 Month	↓*	↓*	↑	[62]
Voiding interval 1 Day	↓*	↓*	↑	[62]
Voiding interval 1 Week	↓*	↓	↑	[62]
Voiding interval 2 Weeks	↓*	↓*	-	[62]
Non-voiding contractions Duration 1 Day	↑*	↓	↓*	[62]
Non-voiding contractions Duration 1 Month	↑	↑*	↑*	[62]
Non-voiding contractions pressure increase 1 Day	↑*	↓	↓*	[62]
Non-voiding contractions pressure increase 1 Month	↓	↑*	↑*	[62]
Non-voiding contractions frequency 1 Day	↑*	↓	↓*	[62]
Non-voiding contractions frequency 1 Week	↑*	↑	↓	[62]
Non-voiding contractions frequency 1 Month	↑	↑*	↑	[62]
Tracheobronchial Scores	-	↑*	-	[68]

Table S3. Effect of craniotomy and TBI on peripheral targets. ↑ - increase in the parameter value in the group, ↓ - decrease in the parameter value in the group, "- "- absence of significant differences between the groups. * - standard level of statistical significance ($p \leq 0.05$), ** - high level of statistical significance ($p \leq 0.01$), *** - high level of statistical significance ($p \leq 0.001$). The presence of arrows without indication of the significance level was noted in cases of absence of statistically significant differences between the groups but corresponded to the results reported by the author in the article for the given parameters. EUS - external urethral sphincter, EMG – electromyographic.
