# Supplementary material for: Disentangling the Effects of Craniotomy and Traumatic Brain Injury in Experimental Models: A Narrative Review
Source: Int J Mol Sci. 2026 Oct 16;27(20):9186. doi: 10.3390/ijms27209186 (PMC13705088; doi:10.3390/ijms27209186)
Supplement: Supplementary file 1 [file ijms-27-09186-s001.zip › Table S4.pdf]

**Table S4.** Effect of craniotomy and TBI on cognitive functions					
**Test**	**Indicator**	**Sham vs Naïve**	**TBI vs Naïve**	**TBI vs Sham**	**Reference**
MWM (working memory)	Latency to find platform	-	↑***	↑***	[34]
	Latency to find platform	-	↑*	-	[79]
MWM (spatial learning and memory)	PC scores 6 months post-TBI (higher values indicate worse impairment)	-	↑	↑	[81]
MWM (spatial learning and memory)	Latency to platform area at 48 h post-injury	-	-	-	[35]
	Time spent in the platform quadrant at 48 h post-injury	-	-	-	[35]
	Latency to platform area at 15 d post-injury	-	↑*	↑*	[35]
	Passes through platform area at 15 d post-injury	↓*	↓*	-	[35]
	Time spent in the platform quadrant at 15 d post-injury	-	↓*	↓*	[35]
MWM (spatial reference memory)	Latency to find platform	-	↑**	↑*	[80]
	Time spent in the areas located symmetrically in the maze	↓***	↓***	-	[80]
MWM (spatial working memory)	Time spent searching for the platform during the test trial	-	↑***	↑***	[78]
	Number of points	-	↓**	-	[78]
MWM (episodic-like memory)	Latency to find platform	-	-	↑*	[80]
	Time spent in the area around the new position of the platform	↓*	↓*	-	[80]
RWM (spatial learning and memory)	Latency to find platform	-	↑***	↑*	[80]
	Number of errors	-	↑*	↑***	[80]
	Number of errors to find cued or hidden platform over 2 days of training	↑*	↑*	-	[80]
	Latency to the cued or hidden platform averaged over 2 days of training	↑*	↑*	-	[80]
	Decision speed	↑*	↑*	-	[80]
	Number of errors to find the previous trial's platform	-	↑**	↑**	[80]
BM	Errors prior to target (learning)	-	↑***	-	[37]
	Errors prior to target (reversal learning)	-	↑***	-	[37]
T-maze spontaneous alternation (spatial working memory)	Correct alternation	-	↓***	↓***	[14]
NORT (retention or intact memory)	Discrimination index	-	↓***	↓***	[14]
NORT (short-term recognition memory)	Discriminatory ratio	-	-	-	[6]
	Time spent exploring the novel object	-	-	-	[6]
NORT (non-spatial hippocampal-mediated short-term memory)	Discriminatory ratio	-	↓*	↓*	[85]
Conditioned passive avoidance reflex	Latent time of transition training	-	↑*	↑*	[85]
	Latent time of transition 10 day	-	-	-	[85]
	Latent time of transition 30 day	-	↓*	-	[85]
The Attentional Set-Shifting Test	Trials to criterion odor	-	-	-	[87]
	Trials to criterion medium	-	-	-	[87]
	Trials to criterion SD	-	-	-	[87]

	Trials to criterion CD	-	-	-	[87]
	Trials to criterion R1	-	-	-	[87]
	Trials to criterion ID	-	-	-	[87]
	Trials to criterion R2	-	-	-	[87]
	Trials to criterion ED	-	↑*	↑*	[87]
	Trials to criterion R3	-	↑*	↑*	[87]
	Total errors SD	-	-	-	[87]
	Total errors CD	-	-	-	[87]
	Total errors R1	-	↑*	-	[87]
	Total errors ID	-	-	-	[87]
	Total errors R2	-	-	-	[87]
	Total errors ED	-	-	-	[87]
	Total errors R3	-	-	-	[87]

Table S4. Effect of craniotomy and TBI on cognitive functions. ↑ - increase in the parameter value in the group, ↓ - decrease in the parameter value in the group, "- "- absence of significant differences between the groups. * - standard level of statistical significance ($p \leq 0.05$), ** - high level of statistical significance ($p \leq 0.01$), *** - high level of statistical significance ($p \leq 0.001$). The presence of arrows without indication of the significance level was noted in cases of absence of statistically significant differences between the groups but corresponded to the results reported by the author in the article for the given parameters. MWM – morris water maze, PC - principal components, RWM - radial water maze, BM - barnes maze, NORT - novel object recognition test, SD - simple discriminations, CD – compound discrimination, R1 – first reversal, ID – intradimensional, R2 – second reversal, ED – extradimensional, R3 - third reversal.
