# Supplementary material for: Disentangling the Effects of Craniotomy and Traumatic Brain Injury in Experimental Models: A Narrative Review
Source: Int J Mol Sci. 2026 Oct 16;27(20):9186. doi: 10.3390/ijms27209186 (PMC13705088; doi:10.3390/ijms27209186)
Supplement: Supplementary file 1 [file ijms-27-09186-s001.zip › Table S5.pdf]

Table S5. Effect of craniotomy and TBI on stress-related and exploratory behavior

**Test**	**Indicator**	**Sham vs Naïve**	**TBI vs Naïve**	**TBI vs Sham**	**Reference**
OF test (risk and anxiety)	Ambulation center	-	↑**	-	[37]
OF test (locomotor activity)	Locomotion	-	-	-	[14]
	Ravel distance	-	-	-	[14]
	Rest time	-	-	-	[14]
	Speed	-	-	-	[14]
	Vertical movements	-	-	-	[14]
	Ravel distance	-	-	-	[14]
OF test (motor and exploratory behavior)	Speed of motor activity 10 day	-	↓*	↓*	[85]
	Speed of motor activity 30 day	-	↓*	↓*	[85]
	Distance travelled	-	↑*	-	[6]
	Number of wall rearing	-	↑*	-	[6]
	Time grooming activity	-	-	-	[6]
	Latency to enter the center of test-arena	-	-	-	[6]
	Time spent in center	-	-	-	[6]
	Time spent in center per entry	↓**	↓**	-	[6]
EPM (avoidance behavior)	Entries into open arms	-	↑*	-	[37]
MCSF (assessment of risk and anxiety)	Being in the upper-right quadrant	↓			[95]
	Being in the lower quadrants	↑			[95]
	Distance covered in the arena during the entire test			-	[95]
	Locomotion			-	[95]
	Exploratory behavior			↑*	[95]
	Risk taking			↑**	[95]
	Shelter seeking			↓*	[95]
	Risk assessment			-	[95]
	DCR (Latency (sec))			↑*	[95]
	Hurdle (Latency (sec))			↓*	[95]
	Slope entrance (Latency (sec))			↓**	[95]
	Rearing (Latency (sec))			↓*	[95]
	Slope (Frequency)			↓* ↑*	[95]
	Central circle (Frequency)			↑*	[95]
Light-dark box test (anxiety-like behavior)	Time in light box	↓	↓	↓	[97]
	General anxiety-like behavior	-	-	-	[98]
	Freezing during Tone (%)	-	-	-	[98]
	Extinction trial number (within session)	-	-	↑	[98]
Nest building (health and welfare)	Scores for 24 hours after injury	↓*	↓**	-	[100]
	Scores for 7 days after injury	-	-	-	[100]
	Scores for 14 days after injury	-	-	-	[100]
Fear conditioning and fear extinction	Fear response, behaviorally expressed as "freezing" Three weeks-post surgery, at the end of the sixth CS-US association	-	-	-	[37]
	At the seventh CS	-	↓	↓	[37]
	After six weeks	↓	↓***	↓***	[37]
Periorbital von Frey sensory test	50% withdrawal threshold ipsi	-	-	-	[105]
	50% withdrawal threshold contra	-	-	-	[105]
	50% withdrawal threshold ipsi 1-day post-injury	↓*	↓*	-	[105]
	50% withdrawal threshold ipsi 3-day post-injury	↓*	↓*	-	[105]

	50% withdrawal threshold ipsi 7-day post-injury	↓*	↓*	-	[105]
	50% withdrawal threshold contra 1-day post-injury	↓*	↓*	-	[105]
	50% withdrawal threshold contra 3-day post-injury	↓*	↓*	-	[105]
	50% withdrawal threshold contra 7-day post-injury	↓*	↓*	-	[105]
	50% withdrawal threshold ipsi 14-day post-injury	↓*	↓*	-	[105]
	50% withdrawal threshold ipsi 21-day post-injury	↓*	↓*	-	[105]
	50% withdrawal threshold ipsi 28-day post-injury	↓*	↓*	-	[105]
	50% withdrawal threshold contra 14-day post-injury	-	↓*	-	[105]
	50% withdrawal threshold contra 21-day post-injury	↓*	↓*	-	[105]
	50% withdrawal threshold contra 28-day post-injury	↓*	↓*	-	[105]
	50% withdrawal threshold ipsi 35-day post-injury	↓*	↓*	-	[105]
	50% withdrawal threshold ipsi 45-day post-injury	-	↓*	-	[105]
	50% withdrawal threshold ipsi 60-day post-injury	-	↓*	-	[105]
	50% withdrawal threshold contra 35-day post-injury	↓*	↓*	-	[105]
	50% withdrawal threshold contra 45-day post-injury	-	↓*	-	[105]

Table S5. Effect of craniotomy and TBI on stress-related and exploratory behavior. ↑ - increase in the parameter value in the group, ↓ - decrease in the parameter value in the group, "- "- absence of significant differences between the groups. * - standard level of statistical significance ($p \leq 0.05$), ** - high level of statistical significance ($p \leq 0.01$), *** - high level of statistical significance ($p \leq 0.001$). The presence of arrows without indication of the significance level was noted in cases of absence of statistically significant differences between the groups but corresponded to the results reported by the author in the article for the given parameters. OF – open field, EPM - elevated plus maze, MCSF - multivariate concentric square field, DCR - dark corner room, CS - conditioned stimulus, US - unconditioned stimulus.
