# Supplementary material for: Disentangling the Effects of Craniotomy and Traumatic Brain Injury in Experimental Models: A Narrative Review
Source: Int J Mol Sci. 2026 Oct 16;27(20):9186. doi: 10.3390/ijms27209186 (PMC13705088; doi:10.3390/ijms27209186)
Supplement: Supplementary file 1 [file ijms-27-09186-s001.zip › Table S6.pdf]

Table S6. Effect of craniotomy and TBI on social behavior and activity cycles

Test	Indicator	Sham vs Naïve	TBI vs Naïve	TBI vs Sham	Reference
Three-chamber test (social motivation)	Preferences in enclosure investigation	-	-	-	[80]
Social recognition task (social recognition memory)	Orientation trial distance	-	-	-	[80]
	Latency to 1st social encounter	-	↑*	-	[80]
	Duration of social interactions	-	-	↓*	[80]
EEG (vigilance state characteristics)	Mean time spent in different vigilance states (Min) – 1 week:<br>Wake	-	-	-	[111]
	Non-REM	-	-	-	[111]
	REM	↑*	-	↓*	[111]
	Mean time spent in different vigilance states (Min) – 1-2 month:<br>Wake	↓*	-	↑*	[111]
	NREM	-	-	↓*	[111]
	REM	-	-	-	[111]
	Average Wake Duration - 1 week	↓*	↓*	-	[111]
	Average NREM Duration - 1 week	-	↑*	↑*	[111]
	Average REM Duration - 1 week	↑*	-	↓*	[111]
	Average Wake Duration - 1-2 month	↓*	↓*	↓*	[111]
	Average NREM Duration - 1-2 month	↑*	-	↓*	[111]
	Average REM Duration - 1-2 month	-	↓*	↓*	[111]
	Spindle dynamics across 24 hours	-	-	-	[111]
	Spindle density in NREM – 1 week	-	-	-	[111]
	Spindle density in NREM – 1-2 month	-	-	-	[111]
	Spindle frequency – 1 week	-	-	-	[111]
	Spindle frequency – 1-2 month	-	-	-	[111]
	Average spindle duration – 1 week	↓*	↓*	-	[111]
	Average spindle duration – 1-2 month	↓*	↓*	-	[111]
	Spindle amplitude – 1 week	↑*	-	↓*	[111]
	Spindle amplitude – 1-2 month	-	-	-	[111]
	NREM spindle power – 1 week	↓*	↓*	↓*	[111]
	NREM spindle power – 1-2 month	↓*	↓*	↓*	[111]

EEG (delta-power)	Normalized delta power – 1 week	↑*	↑*	-	[111]
	Normalized delta power – 1-2 month	↑*	↑*	-	[111]
Post-TBI Operant Drinking	Mean alcohol intake: for 1 day after injury	-	-	-	[97]
	for 2 days post-TBI	-	-	-	[97]
	for 3 days post-TBI	-	-	-	[97]
	for 4 days post-TBI	-	-	-	[97]
	for 5 days post-TBI	-	-	-	[97]
	for 6 days post-TBI	-	-	-	[97]
	for 7 days post-TBI	-	-	-	[97]
	for 8 days post-TBI	-	-	-	[97]
	for 9 days post-TBI	-	-	-	[97]
	Sum change from baseline after TBI	-	↑*	-	[97]
	Sum change from baseline before the TBI: Quartiles 1	↑*	↑*	-	[97]
	Quartiles 2	-	↑*	-	[97]
	Quartiles 3	-	↑*	-	[97]
	Quartiles 4	-	-	-	[97]

Table S6. Effect of craniotomy and TBI on social behavior and activity cycles. ↑ - increase in the parameter value in the group, ↓ - decrease in the parameter value in the group, "- "- absence of significant differences between the groups. * - standard level of statistical significance ($p \leq 0.05$), ** - high level of statistical significance ($p \leq 0.01$), *** - high level of statistical significance ($p \leq 0.001$). The presence of arrows without indication of the significance level was noted in cases of absence of statistically significant differences between the groups but corresponded to the results reported by the author in the article for the given parameters. EEG - epidural electroencephalography, NREM - nonrapid eye movement.
