# Supplementary material for: Disentangling the Effects of Craniotomy and Traumatic Brain Injury in Experimental Models: A Narrative Review
Source: Int J Mol Sci. 2026 Oct 16;27(20):9186. doi: 10.3390/ijms27209186 (PMC13705088; doi:10.3390/ijms27209186)
Supplement: Supplementary file 1 [file ijms-27-09186-s001.zip › Table S7.pdf]

**Table S7.** Effect of craniotomy and TBI on sensorimotor parameters					
**Test**	**Indicator**	**Sham vs Naïve**	**TBI vs Naïve**	**TBI vs Sham**	**Reference**
Neuroscore (reflex testing tasks)	Mean score for days 1–3 post-injury	↑***	↑***	↑***	[34]
	Mean score for 3 months after injury	-	↑***	↑***	[34]
	Mean score for 6 months after injury	-	↑***	-	[34]
	Mean score for 12 months after injury	-	-	-	[34]
Beam Balance (static balance)	Mean score for days 0–3 post-injury	↑*	↑*	↑*	[34]
	Mean score for 3 months after injury	-	↑*	↑*	[34]
	Mean score for 6 months after injury	-	-	-	[34]
	Mean score for 12 months after injury	↑*	↑*	-	[34]
Beam walk (dynamic balance)	Mean score for days 0–3 post-injury	↑*	↑*	↑*	[34]
	Mean score for 3 months after injury	-	-	-	[34]
	Mean score for 6 months after injury	-	-	-	[34]
	Mean score for 12 months after injury	-	-	-	[34]
Rotarod test (physical activity and coordination)	Rotarod Speed 14 day	-	-	↑*	[23]
	Rotarod Speed 30 day	-	-	-	[23]
	Rotarod Speed 14 day	-	-	↑*	[23]
	Rotarod Speed 30 day	-	-	-	[23]
	Rotarod Speed 14 day	-	-	↑*	[23]
	Motor coordination	-	-	-	[6]
Foot Fault test (gait defects)	Front right paw errors	-	-	-	[34]
	Front left paw errors	-	-	-	[34]
	Rear right paw errors	↑**	↑**	-	[34]
	Rear left paw errors	-	↑*	-	[34]
Gait analysis (step base)	Front paw errors	-	-	-	[80]
	Rear paw errors	-	-	-	[80]
Gait analysis (step length)	Right paw errors	-	-	-	[80]
	Left paw errors	-	-	-	[80]
Ladder rung test (gait analysis)	Total score	-	-	-	[6]
Neurobehavioral tests	Suck and swallowing 0 hours after injury	-	-	-	[14]
	Suck and swallowing 1 day after injury	-	↓***	↓***	[14]
	Suck and swallowing 5 days after injury	-	-	-	[14]
	Head movement 0 hours after injury	-	-	-	[14]
	Head movement 1 day after injury	-	↓***	↓***	[14]
	Head movement 5 days after injury	-	-	-	[14]
	Hops 0 hours after injury	-	-	-	[14]
	Hops 1 day after injury	-	↓*	↓*	[14]
	Hops 5 days after injury	-	-	-	[14]
NBS	Test scores for 24 hours after injury	↑*	↑*	↑*	[97]
	Test scores for 72 hours after injury	↑*	↑*	↑*	[97]
	Test scores on 7 day after the injury	-	↑*	↑*	[97]
Neurodevelopmental tests	Eye opening	-	-	-	[14]
	Body elevation	-	-	-	[14]
	Righting	-	-	-	[14]
	Normal gait	-	↑**	↑**	[14]
	Δ Cliff avoidance	-	↑*	↑*	[14]
	Head elevation	-	↑*	-	[14]
	Hopping	-	↑***	↑***	[14]
	Cessation of falling	-	↑**	-	[14]
NSS	Test scores for 24 hours after injury	↑*	↑*	↑*	[97]

	Test scores for 72 hours after injury	↑*	↑*	-	[97]
	Test scores on 7 day after the injury	↑*	↑*		[97]
	Neurological status, points 1 day	-	↑*	↑*	[85]
	Neurological status, points 10 day	-	↑*	↑*	[85]
	Neurological status, points 20 day	-	↑*	↑*	[85]
	Neurological status, points 30 day	-	↑*	↑*	[85]
Grip strength test	Physical strength	↓*	↓*	-	[6]
Prepulse + pulse trials	Startle amplitudes	-	-	-	[131]
An acoustic startle procedure	Sensorimotor reactivity	-	-	↓	[131]
	Ensorimotor reactivity in acoustic and tactile startle procedures	-	↓	-	[131]

Table S7. Effect of craniotomy and TBI on sensorimotor parameters. ↑ - increase in the parameter value in the group, ↓ - decrease in the parameter value in the group, "- "- absence of significant differences between the groups. * - standard level of statistical significance ($p \leq 0.05$), ** - high level of statistical significance ($p \leq 0.01$), *** - high level of statistical significance ($p \leq 0.001$). The presence of arrows without indication of the significance level was noted in cases of absence of statistically significant differences between the groups but corresponded to the results reported by the author in the article for the given parameters. NBS - neurobehavioral scores, NSS - neurological severity scores.
