# Supplementary material for: Disentangling the Effects of Craniotomy and Traumatic Brain Injury in Experimental Models: A Narrative Review
Source: Int J Mol Sci. 2026 Oct 16;27(20):9186. doi: 10.3390/ijms27209186 (PMC13705088; doi:10.3390/ijms27209186)
Supplement: Supplementary file 1 [file ijms-27-09186-s001.zip › Table S8.pdf]

**Table S8.** Effect of craniotomy and TBI on brain metabolic parameters						
**Protein**	**Method**	**Indicator**	**Sham vs Naïve**	**TBI vs Naïve**	**TBI vs Sham**	**Reference**
BDNF	Quantitative RT-PCR	mRNA levels at the parietal lobe ipsi exon 1	↓*	↓**	-	[37]
		mRNA levels at the parietal lobe ipsi exon 4	-	↓*	↓*	[37]
		mRNA levels at the parietal lobe ipsi total	-	↓*	-	[37]
		mRNA levels at the parietal lobe contra	-	-	-	[37]
		mRNA levels at the dorsal hippocampus	-	-	-	[37]
		mRNA levels at the amygdala	-	-	-	[37]
		mRNA levels at the median prefrontal cortex exon 1	↓**	↓*	-	[37]
		mRNA levels at the median prefrontal cortex xon 4	-	↓*	-	[37]
		mRNA levels at the median prefrontal cortex total	-	-	-	[37]
		mRNA levels	-	-	-	[5]
		mRNA levels. 1st day after injury	↓*	↓*	-	[136]
		mRNA levels 2nd days after injury	-	-	-	[136]
		mRNA levels 3rd days after injury	↓*	↑*	↑*	[136]
		mRNA levels 7th days after injury	↓*	↓*	-	[136]
		mRNA levels 14th days after injury	↓*	↓*	-	[136]
		Relative expression level in the ipsilateral cortex	-	-	-	[137]
		Relative expression level in the ipsilateral thalamus	-	-	-	[137]
	Western blot analysis	Relative quantity in the ipsilateral cortex	↓**	↓**	-	[137]
		Relative quantity in the ipsilateral thalamus	--	-	-	[137]
TrkB	RT-PCR	Relative expression level in the ipsilateral cortex	-	↓**	↓**	[137]
		Relative expression level in the ipsilateral thalamus	-	-	-	[137]
GFAP	Immunohistochemistry	Immunoreactivity 7 day	↑***	↑***	↑***	[141]

		Immunoreactivity 14 day	↑*	-	-	[141]
		Immunoreactivity 28 day	-	-	-	[141]
		GFAP cells ipsi CA3	↑**	↑***	↑**	[6]
		GFAP cells contra CA3	-	-	-	[6]
		GFAP-positive area, % in the ipsilateral cortex	↑***	↑***	↑***	[137]
		GFAP-positive area, % in the ipsilateral thalamus	↑**	↑***	↑***	[137]
	Immunostaining (define the lesion location)	GFAP area Contra 0,5 month	-	-	-	[38]
		GFAP area Ipsi 0,5 month	-	↑**	↑**	[38]
		GFAP area Contra 3 month	-	-	-	[38]
		GFAP area Ipsi 3 month	-	↑**	↑**	[38]
	ELISA	Serum GFAP levels 1 hour	-	↑*	↑*	[23]
		Serum GFAP levels 4 hour	-	↑*	↑*	[23]
		Serum GFAP levels 6 hour	-	↑*	↑*	[23]
		Serum GFAP levels 24 hour	-	-	-	[23]
	RPPM	Plasma levels. 2nd day	-	-	↑*	[141]
		Plasma levels. 7th day	-	-	-	[141]
	Quantitative RT-PCR	mRNA levels	-	-	↑*	[141]
		mRNA levels	-	↑*	-	[6]
		Relative expression level in the ipsilateral cortex	↑**	↑***	↑***	[137]
		Relative expression level in the ipsilateral thalamus	-	↑***	↑***	[137]
		Normalized expression in the mouse cerebral cortex	-	↑**	-	[158]
		Normalized expression in the mouse hippocampus	-	↑*	-	[158]
	Western blot analysis	Relative quantity in the serum	↑**	↑***	-	[137]
S100B	ELISA	Serum levels	↑	↑	-	[144]
	RPPM	Plasma levels 2 day	↓*	↓*	-	[141]
		Plasma levels 7 day	-	-	-	[141]
	Western blot analysis	Relative quantity in the serum	↑**	↑***	↑*	[137]

S100a10	RT-PCR	Relative expression level in the ipsilateral cortex	-	-	-	[137]
		Relative expression level in the ipsilateral thalamus	-	-	-	[137]
MBP	Immunohistochemistry	MBP density: corpus callosum	-	↓*	-	[37]
		MBP density: retrosplenial cortex	-	-	↓*	[37]
		MBP density: frontal cortex	-	-	-	[37]
		MBP density: cingulate cortex	-	-	-	[37]
		MBP density: septum	-	-	-	[37]
		MBP density: hippocampus	-	-	↓*	[37]
		MBP density: thalamus	-	-	↓*	[37]
		MBP density: amygdala	-	-	↓*	[37]
		Cortex cytosolic	-	-	-	[157]
	Western blot analysis	Relative quantity in the serum	↑**	↑***	↑***	[137]
UCHL1	RPPM	Plasma levels. 2nd day	-	-	↑*	[141]
		Plasma levels. 7th day	-	-	-	[141]
Vimentin	Immunohistochemistry	Vimentin positive area, % in the ipsilateral cortex	↑***	↑***	↑***	[137]
	RT-PCR	Vimentin positive area, % in the ipsilateral thalamus	-	↑***	↑***	[137]
		Relative expression level in the ipsilateral cortex	-	↑***	↑***	[137]
		Relative expression level in the ipsilateral thalamus	-	↑***	↑***	[137]
	Western blot analysis	Relative quantity in the ipsilateral cortex	-	↑***	↑***	[137]
		Relative quantity in the ipsilateral thalamus	↑*	↑*	-	[137]
		Relative quantity in the serum	↑**	↑***	↑***	[137]
IBA1	Immunostaining analysis	Expression in V1 Contra at 0.5 months post-injury	-	-	-	[38]
		Expression in V1 Ipsi at 3 months post-injury	-	-	-	[38]
		Expression in V1 Ipsi at 0.5 months post-injury	-	↑**	↑**	[38]
		Expression in V1 Contra at 3 months post-injury	-	-	-	[38]

	Immunohistochemistry	Iba-1-positive area, % in the cerebral cortex	↑**	↑***	↑***	[158]
		Iba-1-positive area, % in the thalamus	-	↑***	↑***	[158]
C3	RT-PCR	Relative expression level in the ipsilateral cortex	↑*	↑***	↑***	[137]
		Relative expression level in the ipsilateral thalamus	-	↑***	↑***	[137]
PPAR-α	Western blot analysis	Relative quantity in the ipsilateral cortex	↓*	↓**	-	[137]
		Relative quantity in the ipsilateral thalamus	↓***	↓***	-	[137]
PPAR-γ	Western blot analysis	Relative quantity in the ipsilateral cortex	↓***	↓***	-	[137]
		Relative quantity in the ipsilateral thalamus	↓***	↓***	-	[137]
CatB	Immunohistochemistry	Protein levels in the Frontal Cortex	-	-	↑*	[159]
		Protein levels in the Hippocampus	-	-	-	[159]
	Gel-zymography	Cysteine protease activity. Frontal Cortex 1st day	-	-	-	[159]
		Cysteine protease activity. Frontal Cortex 3rd day	-	-	↑*	[159]
	ELISA	MatCatB Levels. 1st day	-	-	-	[159]
		MatCatB Levels. 3rd day	-	-	↑*	[159]
		MatCatB Levels. 7th day	-	-	-	[159]
Dyn A	Immunohistochemistry	Frontal cortex levels. 24 h	-	-	-	[160]
		Frontal cortex levels. 7 day	↑***	-	↓**	[160]
		Hippocampus levels. 24 h	-	-	-	[160]
		Hippocampus levels. 7 day	↑**	-	↓*	[160]
		Striatum levels. 24 h	↓**	↓*	↓**	[160]
		Striatum levels. 7 day	-	-	↓*	[160]
Oligo2-positive cells	Immunohistochemistry	Number of Olig2-positive cells	-	-	-	[35]
HA	Quantitative RT-PCR	Relative HAS1 mRNA expression 4 hour	↑**	-	-	[161]
		Relative HAS1 mRNA expression 1 day	↑*	-	-	[161]
		Relative HAS1 mRNA expression 3 day	-	↑**	-	[161]

		Relative HAS1 mRNA expression 7 day	-	-	-	[161]
		Relative HAS2 mRNA expression 4 hour	-	-	-	[161]
		Relative HAS2 mRNA expression 1 day	-	↑**	-	[161]
		Relative HAS2 mRNA expression 3 day	-	↑**	-	[161]
		Relative HAS2 mRNA expression 7 day	-	-	-	[161]
		Relative HYAL1 mRNA expression 4 hour	-	-	-	[161]
		Relative HYAL1 mRNA expression 1 day	-	↑*	-	[161]
		Relative HYAL1 mRNA expression 3 day	-	↑*	-	[161]
		Relative HYAL1 mRNA expression 7 day	-	-	-	[161]
		Relative CD44 mRNA expression 4 hour	-	↑*	-	[161]
		Relative CD44 mRNA expression 1 day	-	↑*	-	[161]
		Relative CD44 mRNA expression 3 day	-	↑*	-	[161]
		Relative CD44 mRNA expression 7 day	-	↑*	-	[161]
		Relative RHAMM mRNA expression 4 hour	-	-	-	[161]
		Relative RHAMM mRNA expression 1 day	-	-	-	[161]
		Relative RHAMM mRNA expression 3 day	-	↑*	-	[161]
		Relative RHAMM mRNA expression 7 day	-	-	-	[161]
GLUT4	RT-PCR	Normalized expression in the mouse cerebral cortex	-	↓*	-	[78]
		Normalized expression in the mouse hippocampus	-	↓**	-	[78]
GSK3B	RT-PCR	Normalized expression in the mouse cerebral cortex	-	-	-	[78]
		Normalized expression in the mouse hippocampus	-	-	-	[78]

HK-1	RT-PCR	Normalized expression in the mouse cerebral cortex	-	-	↓*	[78]
		Normalized expression in the mouse hippocampus	-	-	-	[78]
PDHA1	RT-PCR	Normalized expression in the mouse cerebral cortex	-	-	-	[78]
		Normalized expression in the mouse hippocampus	-	-	-	[78]
ENG	RT-PCR	Normalized expression in the mouse cerebral cortex	-	-	-	[78]
		Normalized expression in the mouse hippocampus	-	-	-	[78]
PECAM1	RT-PCR	Normalized expression in the mouse cerebral cortex	-	-	-	[78]
		Normalized expression in the mouse hippocampus	-	-	-	[78]
TGFB1	RT-PCR	Normalized expression in the mouse cerebral cortex	-	-	-	[78]
		Normalized expression in the mouse hippocampus	-	-	-	[78]
CD34	RT-PCR	Normalized expression in the mouse cerebral cortex	-	-	-	[78]
		Normalized expression in the mouse hippocampus	-	-	-	[78]
CD68	Immunohistochemistry	CD68-positive area, % in the cerebral cortex	↑***	↑***	↑***	[158]
		CD68-positive area, % in the thalamus	-	↑***	↑**	[158]
	RT-PCR	Expression of CD68 in cerebral cortex	↑**	↑***	↑***	[158]
		Expression of CD68 in thalamus	↑*	↑***	-	[158]
CD206	RT-PCR	Expression of CD206 in cerebral cortex	-	↑*	↑**	[158]
		Expression of CD206 in thalamus	-	↑***	↑*	[158]
ARG-1	RT-PCR	Expression of Arg-1 in cerebral cortex	-	↑***	↑***	[158]
		Expression of Arg-1 in thalamus	-	↑***	↑***	[158]
BCL-2	Immunohistochemistry	Bcl-2-positive area, % in the ipsilateral cortex	↑***	↑***	↑*	[137]

		Bcl-2-positive area, % in the ipsilateral thalamus	-	↑***	↑***	[158]
	Western blot analysis	Relative quantity in the ipsilateral cortex	↑*	↑***	↑***	[158]
		Relative quantity in the ipsilateral thalamus	-	-	-	[158]
	RT-PCR	Normalized expression in the mouse cerebral cortex	-	-	-	[78]
		Normalized expression in the mouse hippocampus	-	-	-	[78]
BAD	Western blot analysis	Levels in cerebral cortex	↑**	↑***	↑***	[158]
		Levels in thalamus	-	↑***	↑***	[158]
	RT-PCR	mRNA expression in cerebral cortex	-	↑**	↑*	[158]
		mRNA expression in thalamus	-	↑**	↑*	[158]
BAX	Western blot analysis	Levels in cerebral cortex	↑***	↑***	↑***	[158]
		Levels in thalamus	-	↑***	↑***	[158]
	RT-PCR	mRNA expression in cerebral cortex	-	↑*	↑**	[158]
		mRNA expression in thalamus	-	↑***	↑***	[158]
	RT-PCR	Normalized expression in the mouse cerebral cortex	-	-	-	[78]
		Normalized expression in the mouse hippocampus	-	-	-	[78]
CASP9	RT-PCR	Normalized expression in the mouse cerebral cortex	-	↑*	↑*	[78]
		Normalized expression in the mouse hippocampus	-	-	-	[78]
TRP	LC-MS/MS	Level. 7th day	-	-	-	[7]
		Level. 21st day	-	-	-	[7]
KYN	LC-MS/MS	Level. 7th day	-	↑***	↑***	[7]
		Level. 21st day	-	-	-	[7]
	Quantitative RT-PCR	KMO mRNA. 7th day	-	-	-	[7]
		KMO mRNA. 21st day	-	-	-	[7]
Serotonin	LC-MS/MS	Level. 7th day	-	-	-	[7]
		Level. 21st day	-	-	-	[7]
Melatonin	LC-MS/MS	Level. 7th day	-	-	-	[7]

		Level. 21st day	-	↓*	↓*	[7]

Table S8. Effect of craniotomy and TBI on brain metabolic parameters. ↑ - increase in the parameter value in the group, ↓ - decrease in the parameter value in the group, "- "- absence of significant differences between the groups. * - standard level of statistical significance ($p \leq 0.05$), ** - high level of statistical significance ($p \leq 0.01$), *** - high level of statistical significance ($p \leq 0.001$). The presence of arrows without indication of the significance level was noted in cases of absence of statistically significant differences between the groups but corresponded to the results reported by the author in the article for the given parameters. BDNF - brain-derived neurotrophic factor, RT-PCR - real-time polymerase chain reaction, TrkB - tropomyosin-related kinase B, GFAP - glial fibrillary acidic protein, ELISA - enzyme-linked immunosorbent assay, RPPM - reverse phase protein microarray, MBP - myelin basic protein, MAP2 - microtubule associated protein 2, IBA1 - ionized calcium-binding adapter molecule 1, C3 - complement component 3, PPAR-α - peroxisome proliferator-activated receptor α, PPAR-γ - peroxisome proliferator-activated receptor γ, UCHL1 - ubiquitin carboxy-terminal hydrolase L1, Cat-B - cathepsin B, MatCat-B – mature cathepsin B, Dyn A - dynorphin A, HA - hyaluronic acid, HAS - hyaluronan synthases, HYAL – hyaluronidases, RHAMM - receptor of HA mediated motility, GLUT4 - glucose transporter type 4, GSK3B - glycogen synthase kinase 3 beta, HK-1 - hexokinase 1, PDHA1 - pyruvate dehydrogenase E1 alpha subunit, ENG – endoglin, PECAM1 - platelet/endothelial cell adhesion molecule 1, TGFB1 - transforming growth factor beta, CD34 - cluster of differentiation 34, CD68 - cluster of differentiation 68, CD206 - cluster of differentiation 206, ARG-1 - arginase 1, BCL-2 - B-cell lymphoma 2, BAD - BCL2-associated agonist of cell death, BAX - BCL2-associated X protein, CASP9 - Caspase 9, TRP – tryptophan, KYN – kynurenine, KMO – kynurenine-3-monooxygenase, LS-MS/MS – liquid chromatography–tandem mass spectrometry
