# Supplementary material for: Disentangling the Effects of Craniotomy and Traumatic Brain Injury in Experimental Models: A Narrative Review
Source: Int J Mol Sci. 2026 Oct 16;27(20):9186. doi: 10.3390/ijms27209186 (PMC13705088; doi:10.3390/ijms27209186)
Supplement: Supplementary file 1 [file ijms-27-09186-s001.zip › Table S9.pdf]

Table S9. Effect of craniotomy and TBI in protein misfolding and aggregation						
**Protein**	**Method**	**Indicator**	**Sham vs Naïve**	**TBI vs Naïve**	**TBI vs Sham**	**Reference**
β-APP	Immunohistochemistry	Number of β-APP-deposition Total	-	↑*	↑*	[35]
		Number of β-APP-deposition striatum	-	↑*	↑*	[35]
		Number of β-APP-deposition external capsule	-	↑*	↑*	[35]
		Number of β-APP-deposition corpus callosum	-	↑*	↑*	[35]
		Number of β-APP-deposition cingulate gyrus	-	↑*	↑*	[35]
pTau	Histological analysis	P-tau area/total cells at 14 days	↑****	↑***	↑***	[23]
		P-tau area/total cells at 30 days	↑****	↑****	-	[23]
	IHC	Expression of pTAU-Ser199/202-ir Ipsi	↑*	↑*	-	[152]
		Expression of pTAU-Ser199/202-ir Contra	↑*	↑*	-	[152]
	RPPM	Plasma levels of the biomarkers expressed pTAU-Thr231 2 day	↓*	↓*	-	[141]
		Plasma levels of the biomarkers expressed pTAU-Thr231 7 day	-	-	-	[141]
		Plasma levels of the biomarkers expressed pTAU-Thr231/TAU 2 day	-	↓*	-	[141]
		Plasma levels of the biomarkers expressed pTAU-Thr231/TAU 7 day	-	-	↓*	[141]

Table S9. Effect of craniotomy and TBI in protein misfolding and aggregation. ↑ - increase in the parameter value in the group, ↓ - decrease in the parameter value in the group, "- "- absence of significant differences between the groups. * - standard level of statistical significance ($p \leq 0.05$), ** - high level of statistical significance ($p \leq 0.01$), *** - high level of statistical significance ($p \leq 0.001$). The presence of arrows without indication of the significance level was noted in cases of absence of statistically significant differences between the groups but corresponded to the results reported by the author in the article for the given parameters. β-APP - amyloid beta peptide, pTau - tau proteins, IHC – immunohistochemistry, RPPM - reverse phase protein microarray.
